# Actionable loss of SLF2 drives B‐cell lymphomagenesis and impairs the DNA damage response

**DOI:** 10.15252/emmm.202216431

**Published:** 2023-07-24

**Authors:** Le Zhang, Matthias Wirth, Upayan Patra, Jacob Stroh, Konstandina Isaakidis, Leonie Rieger, Susanne Kossatz, Maja Milanovic, Chuanbing Zang, Uta Demel, Jan Keiten‐Schmitz, Kristina Wagner, Katja Steiger, Roland Rad, Florian Bassermann, Stefan Müller, Ulrich Keller, Markus Schick

**Affiliations:** ^1^ Department of Hematology, Oncology and Cancer Immunology, Campus Benjamin Franklin Charité ‐ Universitätsmedizin Berlin, Corporate Member of Freie Universität Berlin and Humboldt‐Universität zu Berlin Berlin Germany; ^2^ German Cancer Consortium (DKTK), German Cancer Research Center (DKFZ) Heidelberg Germany; ^3^ Max‐Delbrück‐Center for Molecular Medicine Berlin Germany; ^4^ Institute of Biochemistry II Goethe University Frankfurt, Medical School Frankfurt Germany; ^5^ Department of Medicine III, Klinikum rechts der Isar Technical University of Munich Munich Germany; ^6^ Center for Translational Cancer Research (TranslaTUM) Technische Universität München Munich Germany; ^7^ Nuclear Medicine, Klinikum rechts der Isar Technical University of Munich Munich Germany; ^8^ Clinician Scientist Program Berlin Institute of Health (BIH) Berlin Germany; ^9^ Comparative Experimental Pathology, Institute of Pathology Technical University of Munich Munich Germany; ^10^ Institute of Molecular Oncology and Functional Genomics, TUM School of Medicine Technische Universität München Munich Germany

**Keywords:** CHK1, DNA damage response, lymphoma, SLF2, SUMO, Cancer, DNA Replication, Recombination & Repair

## Abstract

The DNA damage response (DDR) acts as a barrier to malignant transformation and is often impaired during tumorigenesis. Exploiting the impaired DDR can be a promising therapeutic strategy; however, the mechanisms of inactivation and corresponding biomarkers are incompletely understood. Starting from an unbiased screening approach, we identified the SMC5‐SMC6 Complex Localization Factor 2 (SLF2) as a regulator of the DDR and biomarker for a B‐cell lymphoma (BCL) patient subgroup with an adverse prognosis. SLF2‐deficiency leads to loss of DDR factors including Claspin (CLSPN) and consequently impairs CHK1 activation. In line with this mechanism, genetic deletion of *Slf2* drives lymphomagenesis *in vivo*. Tumor cells lacking SLF2 are characterized by a high level of DNA damage, which leads to alterations of the post‐translational SUMOylation pathway as a safeguard. The resulting co‐dependency confers synthetic lethality to a clinically applicable SUMOylation inhibitor (SUMOi), and inhibitors of the DDR pathway act highly synergistic with SUMOi. Together, our results identify SLF2 as a DDR regulator and reveal co‐targeting of the DDR and SUMOylation as a promising strategy for treating aggressive lymphoma.

## Graphical abstract

SLF2 was identified as a tumor suppressor of B‐cell lymphomagenesis and a crucial regulator of CHK1, and its deficiency was associated with defective DNA damage response and synthetic lethality to SUMOylation inhibition.

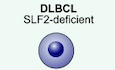

**The paper explained**

**Problem**

Diffuse‐large B cell lymphoma (DLBCL) is a genetically heterogeneous malignancy with poor outcome in about one‐third of patients. Recent large‐scale genomic studies highlighted multiple potential oncogenes and tumor suppressor genes. Pinpointing functionally relevant driver alterations and thus potential actionable biomarkers remains challenging. While altered activity of the DNA damage response (DDR) pathway is frequently involved in lymphomagenesis, specific pharmacological interventions are not established.

**Results**

Starting from an unbiased approach we identified a previously unknown cancer gene, SLF2, which controls a key cancer signaling pathway. Low SLF2 expression was linked to defects in the response to DNA damage and created an actionable molecular vulnerability to the inhibition of SUMOylation, a form of protein modification associated with cellular stress. Loss of SLF2 accelerated tumor onset in a preclinical lymphoma mode, and analysis of SLF2 in samples from DLBCL patients revealed its key role as a biomarker for adverse prognosis. Moreover, defects in the DNA damage response could also be therapeutically introduced by pharmacological inhibition of the DDR driving synergistic cell killing with SUMOylation inhibition.

**Impact**

We show that SLF2 is a clinically relevant tumor suppressor in human aggressive B cell lymphomas and is associated with defective DNA damage response. We propose that loss of SLF2 induces genomic instability and sensitizes cells to SUMOlyation inhibitors thus revealing a therapeutic strategy for a subgroup of B cell lymphoma patients.

## Introduction

The genomic integrity of B‐cells is constantly challenged by physiological activity of the recombination‐activating gene (RAG) complex during V(D)J recombination, by activity of activation‐induced deaminase (AID) during somatic hypermutation (SHM), and by class switch recombination (CSR) (Jung *et al*, 2006). Additionally, activation of the oncogenic transcription factor MYC is a common feature of diffuse large B‐cell lymphoma (DLBCL) (Nguyen *et al*, 2017). Like other oncogenes, MYC induces various intracellular stress pathways including replicative stress and transcriptional stress (Bowry *et al*, 2021), activating the DNA damage response (DDR). DNA repair mechanisms are therefore fundamental for faultless B‐cell development; their failure is postulated to promote genomic instability and malignant transformation into B‐cell lymphomas (BCLs) (Kuppers, 2005; Ciccia & Elledge, 2010). Systematic analysis of coding regions of key DDR and repair genes associated with immunoglobulin (*IG*) gene diversification processes in mature BCLs correspondingly revealed defects in a subset of DDR and repair genes (de Miranda *et al*, 2013).

Diffuse‐large B cell lymphoma is the most frequent BCL accounting for approximately one‐third of all lymph node cancers. Despite increased diagnostic precision (Swerdlow *et al*, 2016) and improved molecular characterization (Reddy *et al*, 2017; Chapuy *et al*, 2018; Pasqualucci & Dalla‐Favera, 2018; Schmitz *et al*, 2018), the clinical perspectives of DLBCL patients remain basically unchanged, with approximately one‐third of DLBCL patients failing first‐line Rituximab‐CHOP‐based immunochemotherapy (Miao *et al*, 2019; Sehn & Salles, 2021). In particular, the high degree of molecular and biological complexity renders it challenging to identify and target functionally relevant drivers of B‐cell lymphomagenesis. Therefore, recent studies have proposed to qualitatively exploit the information from large‐scale OMICs studies by unbiased functional *in vivo* screening for cancer drivers (Weber *et al*, 2019, 2020).

The DNA damage occurring during tumorigenesis typically triggers a cascade of post‐translational modifications (PTMs), among them phosphorylation and de‐phosphorylation, referred to as the DNA damage response (DDR) (Zhou & Elledge, 2000). Proper DDR signaling relies on an intertwined network of PTMs, such as phosphorylation, ubiquitylation, or SUMOylation. The DDR senses DNA damage and transduces the damage signal to downstream cellular pathways, including cell cycle progression, checkpoint activation, apoptosis, and DNA damage repair (Zhou & Elledge, 2000). DNA damage activates the two key DNA damage signaling‐related protein kinases ataxia telangiectasia mutated (ATM) and ATM and Rad3‐related (ATR), contingent on the type of damage and the affected cell cycle phase. While ATM primarily senses double strand breaks (DSBs), ATR primarily senses single strand DNA (ssDNA). Many common substrates of these two kinases, including the checkpoint kinases CHK1 and CHK2, are subsequently phosphorylated to delay cell cycle progression and allow DNA repair (Matsuoka *et al*, 2007; Knittel *et al*, 2018). Constitutive activation of DDR pathways is often observed at pre‐invasive stages of several human cancers and is considered a barrier to tumor progression (Knittel *et al*, 2018). Genetic or non‐genetic alterations affecting these pathways often act as tumor drivers and enable cell proliferation, survival, increased genomic instability, and tumor progression (Bartkova *et al*, 2005). Moreover, specific DDR signaling pathways are frequently impaired during tumorigenesis, creating additional actionable molecular vulnerabilities that can provide tumor‐specific cell death. This clinically established concept implies that BRCA1/2‐mutated cancer cells are highly sensitive to DNA damaging agents, and poly‐(ADP)‐ribose polymerase inhibitors (PARPis) represent a remarkable example of this dependency (Byrum *et al*, 2019). Importantly, the biology leading to this vulnerability can also be driven other than gene mutation‐driven mechanisms of dysregulation. However, biomarkers for an impaired DDR remain poorly understood.

Here, we identify SLF2 as a tumor suppressor of B‐cell lymphomagenesis and a crucial regulator of the CHK1 axis. We link SLF2 deficiency to a defective DDR and establish co‐targeting of the DDR and SUMOylation as a treatment strategy for various aggressive human cancers, including DLBCL.

## Results

### Identification of unappreciated candidate BCL driver genes involved in the DDR

**Figure 1:**
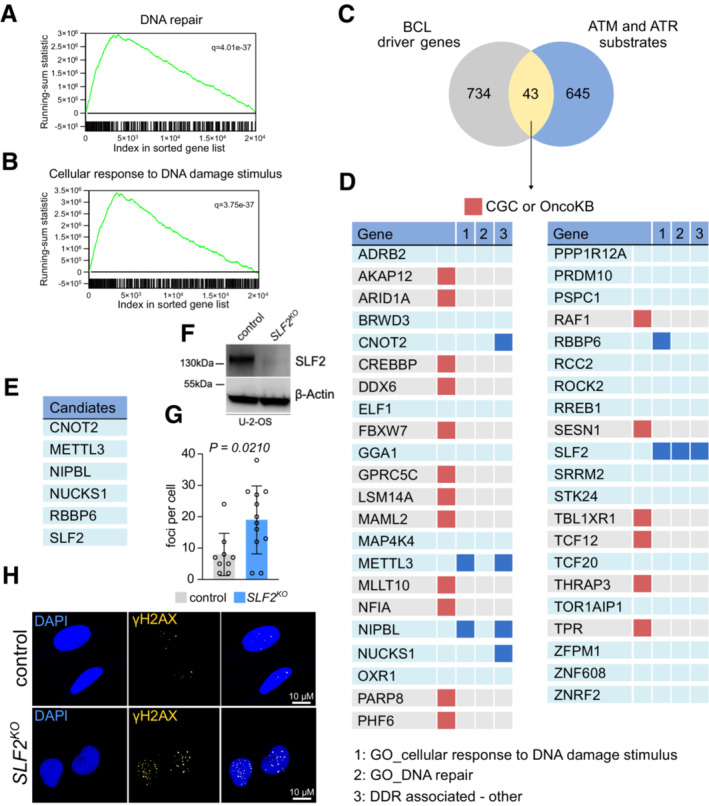
SLF2 is a functionally relevant cancer gene involved in the DNA damage response A, BGSEA of transcriptome data derived from *Eμ‐myc* lymphomas (*n* = 50) and wildtype B‐cells (*n* = 10) with the indicated gene sets. Assessed was GSE7897.CVenn diagram showing the overlap between functionally relevant B‐cell lymphoma cancer genes identified in a recently described transposon mutagenesis screening (Schick *et al*, 2022) and proteins identified as ATR and ATM substrates described in Matsuoka *et al* (2007).DThe 43 overlapping candidate genes are described in (C). Known driver genes listed in the Cancer Gene Census (CGC) or Oncology Knowledge Base (OncoKB) were removed from further analysis. Genes were assessed for a known association with the DDR pathway.EDepiction of the six candidate genes identified in (D).FImmunoblot analysis of SLF2 expression in human U‐2‐OS cell line following CRISPR/Cas9 mediated *SLF2* knockout (KO).GQuantification of immunofluorescence staining from (H). γ.H2.AX foci per cell (control, *n* = 9; *SLF2* ^*KO*^, *n* = 12) were quantified. Data are presented as mean ± SD. *P*‐value was determined by Mann–Whitney test.HImmunofluorescence staining of γ.H2.AX expression of cells described in (G). Source data are available online for this figure.

The activation of oncogenes like MYC typically enhances cell cycle progression and leads to oncogene‐induced replication stress, which results in persistent activation of the DDR indicated by activated forms of DDR proteins like ATR/CHK1 and ATM/CHK2 (Dominguez‐Sola & Gautier, 2014). Tumor cells frequently inactivate specific DDR pathways during the process of malignant transformation (Dominguez‐Sola & Gautier, 2014; Knittel *et al*, 2018). Accordingly, MYC‐driven BCLs showed a striking enrichment of gene sets reflecting activated DDR signaling when compared to control B‐cells (Fig 1A and B, Appendix Figs S1 and S2). To identify previously unknown tumor‐suppressive pathways involved in the DDR, we interrogated a catalog of BCL driver genes (Schick *et al*, 2022) and a dataset of ATM and ATR substrates (Matsuoka *et al*, 2007). Intersecting the two datasets identified 43 candidate cancer genes with potential function in the DDR (Fig 1C). We then removed all known driver genes listed in either the Cancer Gene Census (Sondka *et al*, 2018) or OncoKB (Chakravarty *et al*, 2017) to focus on previously undescribed cancer gene candidates. The remaining genes were scrutinized for a known function in DDR or DNA repair (Fig 1D). Based on this search algorithm, we identified 6 candidates (*CNOT2*, *METTL3*, *NIPBL*, *NUCKS1*, *RBBP6*, and *SLF2*) (Fig 1E), with only SLF2 scoring in all categories (Fig 1D and E).

SLF2 has no known function in cancer yet has been identified as a DNA repair factor that acts as a localization factor of the SMC5/6 complex and other DNA damage and DNA repair factors at DNA damage sites (Raschle *et al*, 2015; Scott *et al*, 2021). To validate the function of SLF2 in the DDR, we generated CRISPR/Cas9‐mediated knockouts (KO) of *SLF2* in U‐2‐OS cells. SLF2 protein expression was substantially reduced in *SLF2* ^*KO*^ cells (Fig 1F), and even without exogenous DNA damage stimulus, we detected a significant accumulation of DSBs indicated by increased γH2AX foci formation (Fig 1G and H), pointing to a functional role of SLF2 in the DDR.

In summary, we here identified SLF2 as a previously unappreciated cancer gene involved in the DDR.

### SLF2 is a tumor suppressor of B‐cell lymphomagenesis

**Figure 2:**
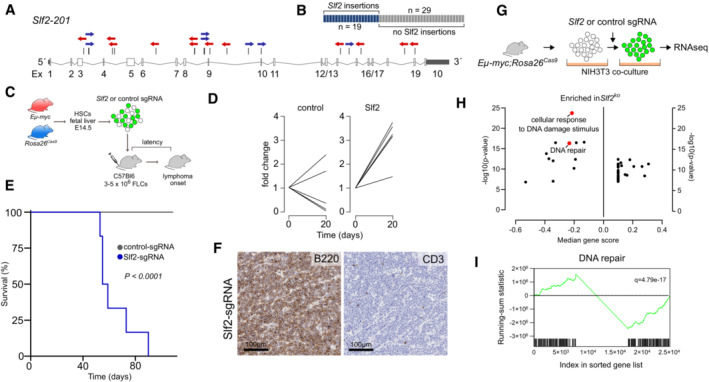
SLF2 restricts MYC‐driven B‐cell lymphomagenesis Transposon insertion pattern in *Slf2* indicating tumor suppressor function. Only the dominant insertions per tumor is shown.Number of B‐cell lymphomas affected by *Slf2* transposon insertions.Experimental transduction/transplantation strategy for *in vivo* validation of the tumor suppressor function of *Slf2*. HSPCs, hematopoietic stem and progenitor cells.Enrichment of control‐sgRNA (*n* = 5) and Slf2‐sgRNA (*n* = 6) transduced HSPCs over time.Kaplan–Meier curves of overall survival of mice transplanted with *Eμ‐myc;Rosa26* ^*Cas9*^ HSPCs transduced with control‐sgRNA (*n* = 15) or sgRNA targeting *Slf2* (*n* = 6). *P* < 0.0001, log‐rank (Mantel‐Cox) test.Immunohistochemical analysis of Slf2‐sgRNA lymphomas described in (E). Representative example of three analyzed Slf2‐sgRNA lymphomas.Experimental workflow for targeting *Slf2* in primary *Eμ‐Myc;Rosa26* ^*Cas9*^ lymphomas and subsequent RNAseq analysis. Primary B‐cell lymphoma cells were transduced with either control‐sgRNA or Slf2‐sgRNA.Summary of GSEA of expression data derived from transcriptome profiling of control‐sgRNA (*n* = 3) and Slf2‐sgRNA transduced (*n* = 3) *Eμ‐Myc;Rosa26* ^*Cas9*^ lymphoma cells with the indicated gene sets.GSEA of expression data derived from transcriptome profiling of the cell lines described in (H) with the indicated gene set.

The recently published *in vivo* screening approach defining the catalog of BCL driver genes (Fig 1C) allows the qualitative assessment of virtually all murine genes in tumorigenesis (Rad *et al*, 2010; Weber *et al*, 2020; Schick *et al*, 2022). Such functional screening provides a first level of *in vivo* evidence. In line with the commonly accepted note that an activated DDR is considered a barrier for malignant transformation, the transposon insertion pattern of the candidate *Slf2* was characterized by scattered and bi‐directional insertions (Fig 2A), revealing that *Slf2* may act as a tumor suppressor gene. Of note, transposon insertions affecting *Slf2* were identified in nearly 40% (19 of 48) of the analyzed tumors, thus suggesting a crucial role of SLF2 in restricting B‐cell lymphomagenesis (Fig 2B). To validate the functional role of SLF2 loss for restricting B‐cell lymphomagenesis *in vivo*, we next applied a CRISPR/Cas9 *in vivo* platform for the validation of tumor suppressor genes (Weber *et al*, 2019). To this end, we generated fetal liver‐derived hematopoietic stem and progenitor cell (FL‐HSPC) grafts from *Eμ‐myc;Rosa26* ^*Cas9*^ mice. The FL‐HSPC pools were transduced with lentivirus encoding a sgRNA targeting *Slf2* or a non‐targeting control sgRNA. We then transplanted these FL‐HSPC grafts into syngeneic wild type mice and assessed for engraftment and tumor onset (Fig 2C). Twenty days after FL‐HSPC transplantation, the fold change of GFP^+^ cells in the peripheral blood of these recipient mice indicated that the *Slf2*‐sgRNA showed a striking enrichment in the blood, whereas the representation of the non‐targeting control sgRNA was not altered, suggesting a direct effect of SLF2 loss in combination with activated MYC signaling in pre‐malignant *Eμ‐myc* B‐cells (Fig 2D, Appendix Fig S3A). Consequently, loss of SLF2 led to a striking acceleration and penetrance of B‐cell lymphomagenesis *in vivo* (Fig 2E), thus validating SLF2 as a functionally relevant tumor suppressor gene. Histology and immunohistochemistry for B220 and CD3 on the tumors arising from the validation experiment verified the B‐cell lymphoma phenotype (Fig 2F). Moreover, we detected insertions and deletions (InDels) in *Slf2*‐sgRNA lymphomas, indicating efficient on‐target gene editing (Appendix Fig S3B). To capture the consequences of SLF2 loss in primary BCLs, we cultured primary control (non‐*PB*) murine *Eμ‐myc;Rosa26* ^*Cas9*^ lymphoma cells and subsequently transduced the B‐cell lymphoma cells with either a non‐targeting control sgRNA or the *Slf2*‐sgRNA used in the *in vivo* validation experiment to deplete SLF2 (Fig 2G). Transduced (GFP^+^) SLF2 KO lymphoma cells were transcriptome profiled. Subsequent gene set enrichment analysis (GSEA) proved that SLF2 deficiency was associated with a defective DDR as indicated by depletion of the gene sets “DNA repair” and “cellular response to DNA damage stimulus” (Fig 2H and I). Thus, we here conclude that SLF2 is a tumor suppressor gene in mice, and link SLF2 loss to an impaired DDR in BCLs.

**Figure 3:**
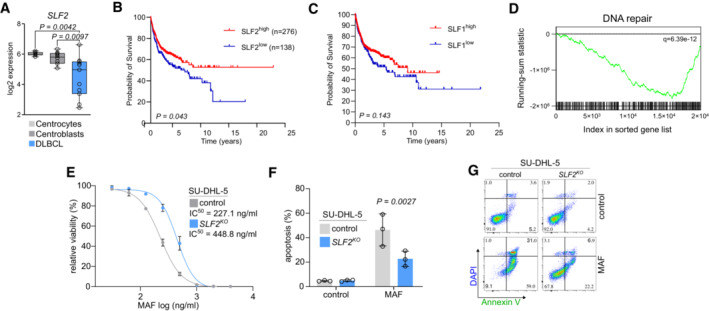
*SLF2* is suppressed in human DLBCL and associated with adverse prognosis A
*SLF2* expression in control B‐cell (centroblasts, *n* = 10; centrocytes, *n* = 7) and in primary DLBCL samples (*n* = 11) in the GSE2350 dataset (Basso *et al*, 2005). The centerline of the box plot is the median. The box extends from the 25^th^ to 75^th^ percentiles. Whisker length is from minimum to maximum. *P*‐value was determined by ANOVA; Tukey's *post hoc* test.B, CKaplan–Meier overall survival curves of the indicated cohorts of DLBCL patients (Lenz, NEJM). DLBCL patients were classified according to *SLF2* or *SLF1* mRNA expression. *P*‐value was determined by log‐rank (Mantel‐Cox) test.DGSEA analysis of DLBCL patients described in (B) with indicated gene set.EMafosphamide dose–response curves of *SLF2* ^*KO*^ and control SU‐DHL‐5 cells. Cells were treated with mafosphamide (MAF; 0, 31.25, 62.5, 125, 250, 500, 1,000, 2,000, 4,000 ng/ml) for 48 h and viability was determined by DAPI staining and flow cytometry measurement. Error bars represent SD from three independent experiments.FQuantification of flow cytometry results for DAPI and Annexin V staining in human *SLF2* ^*KO*^ and control SU‐DHL‐5 cells after MAF treatment (250 ng/ml) for 48 h. *P*‐value determined by two‐way ANOVA; Šídák's multiple comparisons test. Error bars represent SD from three independent experiments.GRepresentative plots of the flow cytometry experiment described in (F).

To stress the relevance of these findings for human BCL, we analyzed a representative human DLBCL dataset for *SLF2* mRNA expression (Basso *et al*, 2005). *SLF2* expression was significantly reduced in primary human DLBCL patient samples when compared to germinal center B‐cells (Fig 3A). Moreover, low SLF2 expression was associated with the adverse prognosis of DLBCL patients (Fig 3B, Appendix Fig S4), which was not observed for SMC5‐SMC6 Complex Localization Factor 1 (SLF1; Fig 3C). Importantly, in line with the biology associated with *SLF2* deficiency, GSEA revealed a deficiency of the DDR in SLF2^low^ DLBCL patients (Fig 3D). To investigate an adverse association of SLF2 expression and susceptibility to cyclophosphamide, a standard drug for DLBCL first‐line therapy, we treated human SU‐DHL‐5 DLBCL cells lacking SLF2 with increasing doses of mafosfamide (MAF, active form of cyclophosphamide). Of note, SU‐DHL‐5 *SLF2* ^*KO*^ cells showed a significantly impaired sensitivity to MAF/cyclophosphamide as compared to SU‐DHL‐5 control cells (Fig 3E–G).

Together, these data identify SLF2 as a functionally relevant tumor suppressor in murine and human BCL. Moreover, low *SLF2* expression is associated with adverse prognosis in DLBCL patients and reduced sensitivity to a standard lymphoma drug.

### SLF2 deficiency impairs the ATR‐CLSPN‐CHK1 axis

**Figure 4:**
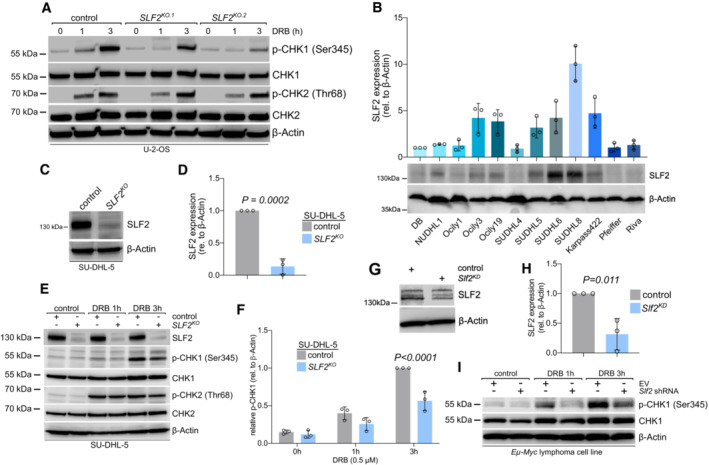
SLF2 deficiency leads to impaired CHK1 axis Immunoblot analysis of U‐2‐OS cells following CRISPR/Cas9 mediated knockout of *SLF2* upon DRB treatment (0.5 μM) for indicated times with the indicated antibodies.Immunoblot analysis of a panel of human DLBCL cell lines with a specific SLF2 antibody and quantification of the SLF2 western blots (middle panel). Protein expression of SLF2 was normalized to β‐Actin expression. The expression level of SLF2 in DB cells was set as 1. Data are presented as mean ± SD of *n* = 3 independent experiments.Immunoblot analysis of SU‐DHL‐5 cells following CRISPR/Cas9 mediated *SLF2* knockout (KO) with the indicated antibodies.Quantification of the SLF2 western blots in (C). Protein expression of SLF2 was normalized to β‐Actin expression. Data are presented as mean ± SD of *n* = 3 independent experiments. *P*‐value determined by unpaired *t*‐test.Immunoblot analysis of human SU‐DHL‐5 control and *SLF2* ^*KO*^ cell lines upon DRB treatment (0.5 μM) for indicated time points with the indicated antibodies.Quantification of the p‐CHK1 western blots from Fig 5E. Protein expression of p‐CHK1 was normalized to β‐Actin protein expression. p‐CHK1 expression in *SU‐DHL‐5* control cells at 3 h doxorubicin treatment was arbitrarily set to 1. Data are presented as mean ± SD of *n* = 3 independent experiments. *P*‐value determined by two‐way ANOVA; Šídák's multiple comparisons test.Immunoblot analysis of *Eμ‐Myc* lymphoma cell line transduced with either a control vector or an shRNA targeting *Slf2*.Quantification of the SLF2 western blots in (G). Protein expression of SLF2 was normalized to β‐Actin expression. Data are presented as mean ± SD of *n* = 3 independent experiments. *P*‐value determined by unpaired *t*‐test.Immunoblot analysis of murine *Eμ‐myc* lymphoma cell line after transduction with either empty vector or specific *Slf2* shRNA with the indicated antibodies. Cells were treated with DRB (0.5 μM) for indicated time points. Source data are available online for this figure.

Ataxia telangiectasia mutated and ATR are among the key regulators/effectors of the DDR and initiate a cascade of phosphorylation and de‐phosphorylation events to control cell cycle arrest and DNA repair (Matsuoka *et al*, 2007; Smith *et al*, 2020). Following activation, ATR and ATM phosphorylate and thus activate the downstream kinases CHK1 and CHK2 respectively (Smith *et al*, 2020). Considering that SLF2 was previously identified as a substrate of the ATR and ATM kinases (Matsuoka *et al*, 2007), we hypothesized that SLF2 might be involved in regulating the activation of the downstream kinases CHK1 and CHK2. To test this concept, we treated *SLF2* ^*KO*^ U‐2‐OS cells with doxorubicin (DRB) to promote checkpoint activation. Depletion of SLF2 led to a striking reduction of CHK1 activation, whereas activation of CHK2 was not affected (Fig 4A). CHK1 is instrumental for the ATR‐mediated response to replicative stress and delays cell cycle progression to allow DNA repair and maintenance of genomic integrity (Michelena *et al*, 2019). To stress the function of SLF2 in limiting CHK1 activation in human BCL, we analyzed SLF2 protein expression in a panel of DLBCL cell lines (Fig 4B). In line with the broad spectrum of mRNA expression observed in primary DLBCL patient samples (Fig 3A), we detected a broad spectrum of SLF2 protein levels in patient‐derived DLBCL cell lines (Fig 4B). Based on this analysis, we selected the cell line SU‐DHL‐5, which showed intermediate SLF2 protein expression, for further loss‐of‐function experiments. We generated a CRISPR/Cas9‐mediated *SLF2* KO in SU‐DHL‐5 cells resulting in reduced SLF2 protein expression in the *SLF2* ^*KO*^ cells as compared to control cells (Fig 4C and D). We then treated the *SLF2* ^*KO*^ and control cells with DRB to promote checkpoint activation. In line with our previous finding from U‐2‐OS cells, phosphorylation of CHK1 was significantly reduced, whereas the CHK2 axis was not substantially affected (Fig 4E and F, Appendix Fig S5A–F). Of note, we also observed an inverse correlation between *SLF2* mRNA expression and the number of mutations in DLBCL patient samples further corroborating the role of SLF2 in the maintenance of genome integrity (Appendix Fig S5G).

In addition, we observed the same effect of SLF2 loss on the CHK1 axis following hydroxyurea and aphidicolin stimulus (Appendix Fig S6). Moreover, transcriptome profiling and subsequent GSEA indicated impaired DDR in SU‐DHL‐5 *SLF2* ^*KO*^ cells, further underscoring the critical function of SLF2 in the DDR (Dataset EV1). Depletion of SLF2 in murine lymphoma cell lines derived from primary *Eμ‐myc* lymphomas resulted in the compromised activation of CHK1 (Fig 4G–I), thus revealing a highly conserved mechanism regulating the CHK1 axis by SLF2.

**Figure 5:**
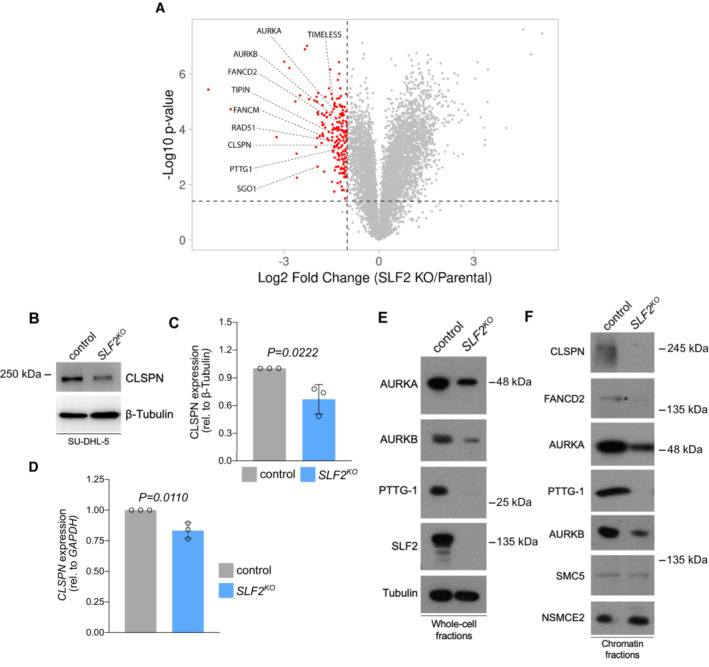
SLF2 deficiency leads to loss of CLSPN and key DDR and cell cycle regulators TMT‐based MS results of chromatin fractions from SU‐DHL‐5 control and SU‐DHL‐5 *SLF2* ^*KO*^ cells. Volcano plot depicting depleted chromatin‐associated proteins. Significant hits are shown by red dots (depleted in *SLF2* ^*KO*^ cells). Proteins belonging to the DDR and cell cycle regulation are labeled. Experiments were performed in triplicates.Immunoblot analysis of human SU‐DHL‐5 control and *SLF2* ^*KO*^ cell lines with the indicated antibodies.Quantification of the CLSPN western blots in (B). Protein expression of CLSPN was normalized to β‐Tubulin expression. Data are presented as mean ± SD of *n* = 3 independent experiments. *P*‐value determined by unpaired *t*‐test.qPCR analysis of CLSPN mRNA expression of SU‐DHL‐5 control and *SLF2* ^*KO*^ cell lines. Data are presented as mean ± SD of *n* = 3 independent experiments. *P*‐value determined by unpaired *t*‐test.Immunoblot analysis of human SU‐DHL‐5 control and *SLF2* ^*KO*^ whole cell fractions with the indicated antibodies.Immunoblot analysis of human chromatin fractions of SU‐DHL‐5 control and *SLF2* ^*KO*^ cell lines with the indicated antibodies. Source data are available online for this figure.

To unravel the molecular mechanism which leads to impaired CHK1 activation following SLF2 loss, we undertook an unbiased mass spectrometry‐based approach on SU‐DHL‐5 parental and *SLF2* ^*KO*^ cells. Considering that SLF2 is best characterized as a chromatin recruitment factor for DNA repair and chromatin remodeling factor (Raschle *et al*, 2015; Scott *et al*, 2021), we aimed to understand how loss of SLF2 affects the chromatin landscape. To this end, we studied the chromatin proteome of the parental and SLF2‐deficient SU‐DHL‐5 cells. The data revealed 189 proteins with at least 2‐fold reduction in chromatin association in cells lacking SLF2 (Dataset EV2, Fig 5A). Pathway enrichment analysis demonstrated that many factors which exhibited reduced chromatin association in SLF2‐deficient cells were involved in mitotic cell cycle progression and sister chromatin segregation (Table EV1). These factors included Aurora kinases A and B as well as the cohesin regulator shugoshin1 (Fig 5A). Another group of proteins, whose chromatin association was affected upon SLF2 loss belongs to regulators of DDR, such as components of the Fanconi anemia repair pathway and the RAD51‐mediated HR machinery (Fig 5A). Intriguingly, chromatin residency of Claspin, which facilitates the ATR‐dependent phosphorylation of CHK1, was also strongly reduced in absence of SLF2 (Fig 5A). Of note, analysis of the whole cell proteome confirmed depleted levels of DDR factors including CLSPN, and regulators of mitotic cell cycle progression as well as sister chromatin segregation (Appendix Fig S7, Dataset EV3), partially explaining the dysregulated chromatin proteome landscape. Importantly, in line with the pathway analysis and the crucial role of the DDR for cell cycle progression, SU‐DHL‐5 *SLF2* ^*KO*^ cells showed an altered cell cycle and failed to arrest in G2/M following DRB pulse (Appendix Fig S8). We validated CLSPN deficiency as a result of SLF2 loss by immunoblotting of whole cell lysates isolated from parental and SU‐DHL‐5 *SLF2* ^*KO*^ (Fig 5B and C). While inhibition of ubiquitylation and proteasomal degradation did not affect the depletion of CLSPN (Appendix Fig S9), *CLSPN* mRNA was depleted to a similar extent as CLSPN protein expression, thus revealing an effect of SLF2‐deficiency on the transcriptional or post‐transcriptional regulation of CLSPN (Fig 5D). Moreover, we validated the loss of expression of AURKA, AURKB, PTTG‐1, FANCD2 and CHK1 (Fig 5E and F). Altogether, these data demonstrate that loss of SLF2 affects the expression and chromatin presence of a subset of proteins involved in the control of genome integrity and DDR, including the critical CHK1 regulator CLSPN.

Importantly, CLSPN promotes ATR‐dependent phosphorylation of CHK1 by bringing CHK1 into proximity with ATR (Liu *et al*, 2006; Saldivar *et al*, 2017). Therefore, the loss of CLSPN that is associated with SLF2 deficiency is in agreement with impaired CHK1 activation in *SLF2* ^*KO*^ cells. Of note, a recent study provided genetic evidence that CLSPN‐deficient mice developed spontaneous B‐cell lymphomas (preprint: Hunter *et al*, 2018). Together, these findings indicate a mechanism by which CLSPN loss subsequent to SLF2 deficiency may explain the tumor suppressor function of SLF2, while SLF2 also acts as a regulator of several factors involved in cell cycle progression and DNA repair.

In summary, our data identify SLF2 as an evolutionarily conserved regulator of the DDR and as a crucial factor for the maintenance of genomic integrity.

### SLF2 loss drives alteration of the SUMO pathway and confers synthetic lethality to SUMOylation inhibition

**Figure 6:**
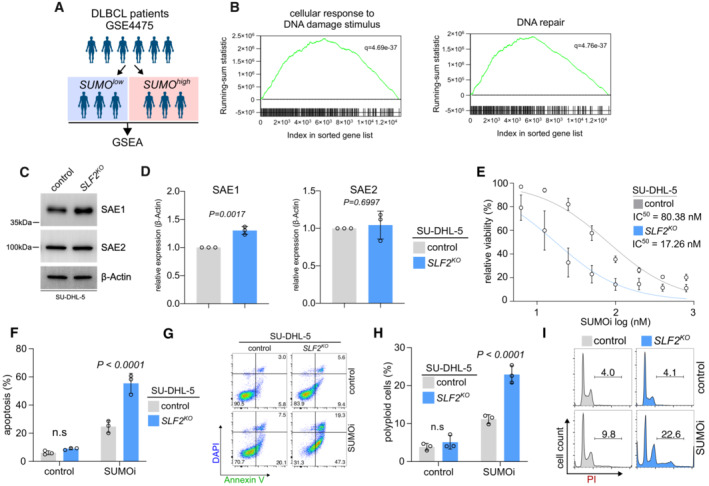
SLF2 loss drives activation of the SUMO pathway and confers synthetic lethality to SUMOi A, BDLBCL patients were classified as SUMO^high^ and SUMO^low^ group in the GSE4475 dataset. GSEA of expression data of the SUMO^high^ cohort with the indicated gene sets.CImmunoblot analysis of human SU‐DHL‐5 control and *SLF2* ^*KO*^ cell lines with the indicated antibodies.DQuantification of the SAE1 and SAE2 western blots from (C). Protein expression of SAE1 and SAE2 was normalized to β‐Actin protein expression. Data are presented as mean ± SD of *n* = 3 independent experiments. *P*‐value determined by unpaired *t*‐test.ESUMO inhibitor (SUMOi, TAK981; 0, 6.25, 12.5, 25, 50, 100, 200, 400, 800 nM) dose response curves of *SLF2* ^*KO*^ and control SU‐DHL‐5 cells. Cells were treated with increasing concentrations of SUMOi for 72 h and viability was determined by negative DAPI staining and flow cytometry measurement. Error bars represent SD from three independent experiments.FQuantification of flow cytometry results for DAPI and Annexin V staining in human *SLF2* ^*KO*^ and control SU‐DHL‐5 cells after 12.5 nM of SUMOi treatment for 72 h. *P*‐value determined by unpaired *t*‐test. Error bars represent SD from three independent experiments.GRepresentative plots of the flow cytometry experiment described in (F).HQuantification of flow cytometry results for PI cell cycle analysis in human *SLF2* ^*KO*^ and control SU‐DHL‐5 cells after 12.5 nM of SUMOi treatment for 72 h. *P*‐value determined by unpaired *t*‐test. Error bars represent SD from three independent experiments.IRepresentative histograms of the flow cytometry experiment described in (H). Source data are available online for this figure.

Pathways mediating the DDR are often impaired during tumorigenesis and we identified altered DDR in SLF2‐deficient lymphomas. Loss of these pathways typically leads to hyperactivity of compensating pathways and thereby creates a window for synthetic lethality (Burdak‐Rothkamm & Rothkamm, 2021). To specifically identify actionable pathways in SLF2‐deficient cells, we performed pathway analysis based on the mass‐spec characterization of SU‐DHL‐5 *SLF2* ^*KO*^ cells (Table EV1). Among all altered pathways identified, one strong candidate pathway potentially activated in response to DNA damage triggered by SLF2 loss was the post‐translational SUMOylation pathway (Table EV1), which has previously been described as a stress response safeguard (Cremona *et al*, 2012; Psakhye & Jentsch, 2012; Seeler & Dejean, 2017; Kroonen & Vertegaal, 2020). To further corroborate the association of SUMOylation and the DDR, we classified DLBCL patients into SUMO^high^ and SUMO^low^ based on transcriptome profiles and performed GSEA based on expression of the SUMO core machinery (Demel *et al*, 2022) genes UBE2I, SAE1, SAE2, SUMO1, SUMO2 and SUMO3 (Fig 6A). Indeed, we identified enrichment of the gene sets “cellular response to DNA damage stimulus” and “DNA repair” (Fig 6B) in human SUMO^high^ DLBCL, thus linking the DDR and the SUMOylation pathway. SUMOylation is a crucial post‐translational modification that regulates several cellular processes such as chromosome segregation, cell cycle progression, and DNA repair (Seeler & Dejean, 2017). Importantly, SUMOylation is a highly dynamic and fully reversible process. Regulation of the SUMO state involves SUMO activation/conjugation/ligation by activity of E1 (SAE1/SAE2), E2 (UBE2I) and E3 enzymes, while SUMO iso‐peptidases (SENPs) cleave SUMO modifications from target proteins (Seeler & Dejean, 2017). To investigate specifically the SUMO conjugation pathway in the context of the SLF2 status, we analyzed the expression of the key SUMO E1 enzyme components SAE1/SAE2 in SU‐DHL‐5 cells. Of note, SAE1 protein expression was significantly higher in SLF2‐deficient cells when compared to control cells (Fig 6C and D), possibly indicating SUMOylation activation and/or dependency.

Based on the altered SUMOylation pathway associated with SLF2 loss, we hypothesized that SLF2 loss could create an actionable molecular vulnerability towards inhibition of SUMOylation. To test this putative vulnerability caused by SLF2 deficiency and altered SUMOylation, we investigated the efficacy of a first‐in‐class small‐molecule inhibitor of SUMOylation (SUMOi), TAK‐981/subasumstat (Lightcap *et al*, 2021), which is tested in clinical phase I/II trials in lymphoma patients (NCT03648372). Of note, SLF2 loss in SU‐DHL‐5 cells (SU‐DHL‐5 *SLF2* ^*KO*^) conferred synthetic lethality to SUMOi (Fig 6E) and led to significantly higher induction of apoptotic cell death compared to control cells (Fig 6F and G). The effects of SUMOi were previously linked to cell cycle defects and polyploidy in cancer cells (Hoellein *et al*, 2014; Biederstadt *et al*, 2020). Fully in line with this position, SUMOi treatment potentiated polyploidy in the SU‐DHL‐5 *SLF2* ^*KO*^ cell line (Fig 6H and I). While depletion of SLF2 did not affect the sensitivity to SUMOi in non‐cancer cell lines (Appendix Fig S10), we could validate the synthetic lethality driven by SLF2 loss in DLBCL in OCI‐Ly19 *SLF2* ^*KO*^ lymphoma cells (Appendix Fig S11A and B, Dataset EV1). Moreover, SLF2‐deficient lymphoma cells did not show any sensitization to a ubiquitin E1 inhibitor (Appendix Fig S11C) further underscoring the specificity of the synthetic lethality to SUMOi.

Taken together, we conclude that SLF2 loss leads to altered SUMO pathway activity and confers synthetic lethality to SUMOi.

### CLSPN loss results in effects similar to the effects of SLF2 loss

**Figure 7:**
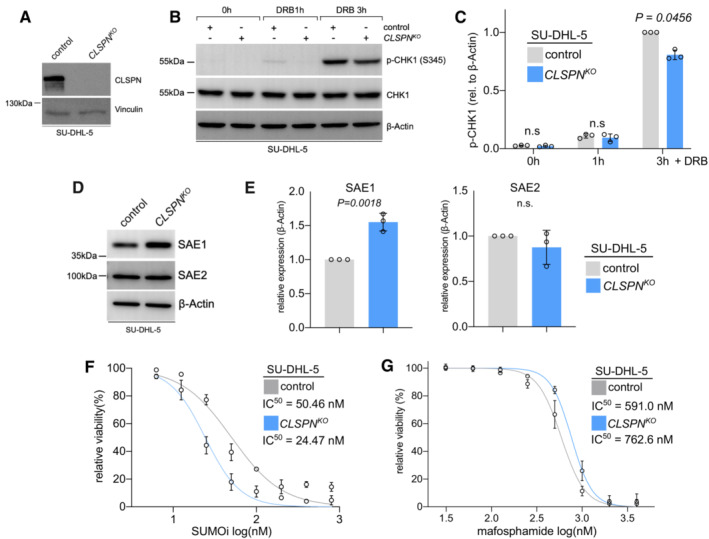
CLSPN loss results in effects similar to the effects of SLF2 loss Immunoblot analysis of SU‐DHL‐5 cells following CRISPR/Cas9 mediated *CLSPN* knockout (KO) with the indicated antibodies.Immunoblot analysis of human SU‐DHL‐5 control and *CLSPN* ^*KO*^ cell lines upon DRB treatment (0.5 μM) for indicated time points with the indicated antibodies.Quantification of the p‐CHK1 western blots from (B). Protein expression of p‐CHK1 was normalized to β‐Actin protein expression. p‐CHK1 expression in control cells at 3 h of doxorubicin treatment was arbitrarily set to 1. Data are presented as mean ± SD of *n* = 3 independent experiments. *P*‐value determined by two‐way ANOVA; Šídák's multiple comparisons test.Immunoblot analysis of human SU‐DHL‐5 control and *CLSPN* ^*KO*^ cell lines with the indicated antibodies.Quantification of the SAE1 and SAE2 western blots from (E). Protein expression of SAE1 and SAE2 was normalized to β‐Actin protein expression. SAE1 and SAE2 expression in control cells were arbitrarily set to 1. Data are presented as mean ± SD of *n* = 3 independent experiments. *P*‐value determined by unpaired *t*‐test.SUMO inhibitor (SUMOi, TAK981; 0, 6.25, 12.5, 25, 50, 100, 200, 400, 800 nM) dose–response curves of *CLSPN* ^*KO*^ and control SU‐DHL‐5 cells. Cells were treated with increasing concentrations of SUMOi for 72 h and viability was determined by negative of DAPI staining and flow cytometry measurement. Error bars represent SD from three independent experiments.Mafosphamide dose–response curves of *CLSPN* ^*KO*^ and control SU‐DHL‐5 cells. Cells were treated with mafosphamide (MAF; 0, 31.25, 62.5, 125, 250, 500, 1,000, 2,000, 4,000 ng/ml) for 48 h and viability was determined by negative of DAPI staining and flow cytometry measurement. Error bars represent SD from three independent experiments. Source data are available online for this figure.

So far, our data suggest that SLF2 loss creates a dependency on the SUMOylation pathway. Of note, we identified SLF2 as a critical factor for a functional DDR and a regulator of CLSPN, a key regulator of the CHK1 axis (Smits *et al*, 2019). To further corroborate the molecular basis for this SUMOi vulnerability, we investigated the effect of the CLSPN status on the sensitivity to SUMOi. First, we generated a CRISPR/Cas9‐mediated *CLSPN* ^*KO*^ SU‐DHL‐5 cell line. CLSPN protein expression was reduced to background levels (Fig 7A), and, as expected, we observed compromised CHK1 phosphorylation in *CLSPN* ^*KO*^ cells (Fig 7B and C). Similar to SLF2‐deficient cells, we found significantly increased SAE1 expression in CLSPN‐deficient cells (Fig 7D and E) and CLSPN loss conferred sensitivity to SUMOi (Fig 7F). Moreover, *CLSPN* ^*KO*^ cells were also more resistant to mafosphamide (Fig 7G), to an extent similar as upon SLF2 loss (Fig 3E).

In summary, our data reveal that CLSPN loss and the consequent impairment of CHK1 activation compromised the DDR and resulted in effects similar to SLF2 loss and were also sufficient to drive sensitivity to SUMOi.

### SUMOi and CHK1i act synergistic *in vivo*

**Figure 8:**
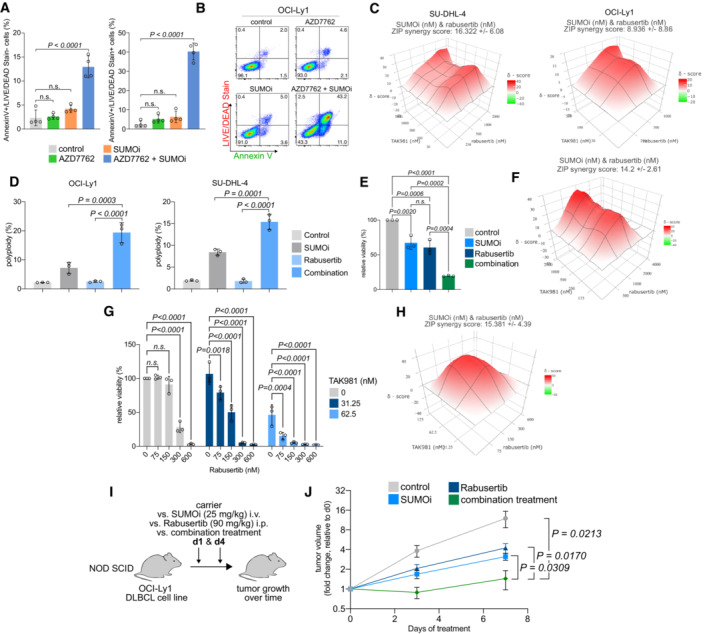
SUMOylation inhibition and CHK1 inhibition Quantification of flow cytometry results for LIVE/DEAD® Fixable Aqua Dead Cell Stain and Annexin V staining in human OCI‐Ly1 cells after SUMOi (15 nM) and AZD7762 (110 nM) co‐treatment for 72 h. *P*‐value was determined by ANOVA; Tukey's *post hoc* test. Error bars represent SD from three independent experiments.Representative histograms of the flow cytometry experiment described in (A).ZIP synergy score plot of human SU‐DHL‐4 and OCI‐Ly1 cells co‐treated with increasing concentrations of SUMOi and Rabusertib. The presented ZIP synergy scores are the average of *n* = 3 independent experiments.Quantification of flow cytometry results for PI cell cycle analysis in human OCI‐Ly1 (SUMOi, 15 nM; Rabusertib, 200 nM) and SU‐DHL‐4 (SUMOi, 30 nM; Rabusertib, 500 nM) cells after co‐treatment with SUMOi and rabusertib treatment for 72 h. *P*‐value was determined by ANOVA; Tukey's *post hoc* test. Error bars represent SD from three independent experiments.Quantification of cell viability measured by celltiterGlo from murine *Eμ‐Myc* lymphoma‐derived cell line co‐treated with SUMOi (500 nM) and Rabusertib (1,000 nM) for 72 h. Bar plots represent cell viability relative to the DMSO‐treated control cells. Error bars represent SD from three independent experiments. *P*‐value was determined by ANOVA; Tukey's *post hoc* test.ZIP synergy score plot of primary murine *Eμ‐Myc* lymphoma cell line co‐treated with increasing concentrations of SUMOi and Rabusertib for 72 h. The presented ZIP synergy scores are the average of *n* = 3 independent experiments.Quantification of cell viability measured by celltiterGlo from primary murine *Eμ‐Myc* lymphoma‐derived cells co‐treated with indicated concentrations of SUMOi and Rabusertib for 72 h. Bar plots represent cell viability relative to the DMSO‐treated control cells. Error bars represent SD from three independent experiments. *P*‐value was determined by ANOVA test; Tukey's *post hoc* test.ZIP synergy score plot of primary murine *Eμ‐Myc* lymphoma cells co‐treated with increasing concentrations of SUMOi and Rabusertib for 72 h. The presented ZIP synergy scores are the average of *n* = 3 independent experiments.The human DLBCL cell line OCI‐Ly1 was used to generate murine xenograft models in NOD SCID mice. Mice were treated with vehicle, 25 mg/kg SUMOi (TAK‐981), 90 mg/kg Rabusertib or the combination of SUMOi and Rabusertib on days 1 and 4.Tumor size was measured over time (*n* = 4 mice in each group). Error bars represent SEM. *P*‐value determined by unpaired *t*‐test.

The finding that CLSPN loss alone was sufficient to confer synthetic lethality to SUMOi indicated that interference with the CHK1 axis/DDR could create an actionable molecular vulnerability towards the SUMOylation pathway. To test whether co‐targeting of the DDR and the SUMOylation pathway could be advanced towards a potential novel therapeutic strategy beyond SLF2 loss, we co‐treated the intrinsically SUMOi‐resistant DLBCL cell line OCI‐Ly1 with a combination of SUMOi (subasumstat) and the CHK1/2 inhibitor AZD7762. Whereas each inhibitor alone did not induce relevant cell death or apoptosis combination treatment acted highly synergistic and significantly potentiated apoptotic cell death (Fig 8A and B). Moreover, considering the impaired activation of CHK1 but not CHK2 that we observed following SLF2 loss, the synergism of SUMOi with the highly specific CHK1 inhibitor rabusertib (King *et al*, 2014) was even more pronounced in OCI‐Ly1 and SU‐DHL‐4 cells, another intrinsically SUMOi‐resistant DLBCL cell line (Fig 8C). To interrogate the potential applicability of the synergism of SUMOi and CHK1i as a more general concept for cancer treatment, we tested the combination in a cell line panel reflecting various aggressive cancers. Notably, pharmacological SUMOi with subasumstat and CHK1i (rabusertib or prexasertib) acted highly synergistic in all tested cell lines (Appendix Fig S12), thus proposing a combination treatment strategy of SUMOi and CHK1i as a novel concept for the treatment of aggressive human cancers. We could further validate this combination treatment strategy in a murine *Eμ‐Myc* lymphoma‐derived cell line (Fig 8E and F) and primary murine *Eμ‐Myc* lymphoma cells (Fig 8G and H). The combination of SUMOi and CHK1i resulted in similar cell cycle aberrations like we observed for SLF2‐deficient cells following SUMOi treatment (Fig 8D, Appendix Fig S13), also indicating that the compromised CHK1 axis following SLF2 loss drives synthetic lethality to SUMOi. Indeed, inhibition of SUMOylation substantially compromised the DDR (Appendix Fig S14A–C).

To further support the combination of SUMOi and CHK1i as a therapeutic concept for future clinical translation, we generated xenografts of the OCI‐Ly1 DLBCL cell line. Upon tumor formation, we treated the xenograft‐bearing mice with either carrier control, subasumstat (SUMOi), rabusertib (CHK1i), or the combination (SUMOi + CHK1i) and monitored tumor growth (Fig 8I). SUMOi + CHK1i combination treatment with subasumstat and rabusertib led to significantly reduced growth of OCI‐Ly1 xenograft tumors compared to SUMOi or rabusertib treatment alone (Fig 8J). Importantly, we also observed efficacy of this combination on tumor growth in a syngeneic lymphoma mouse model (Appendix Fig S14D).

In summary, we show that DDR deficiency, e.g., caused by SLF2 loss or inhibition of the DDR, confers synthetic lethality to SUMOi. As further conceptual advancement, we suggest co‐targeting of the SUMO pathway and the CHK1 axis (Appendix Fig S11D).

## Discussion

We here combined the results from a recently published genome‐scale cancer gene discovery screen (Schick *et al*, 2022) and a comprehensive description of ATM and ATR targets (Matsuoka *et al*, 2007) to identify novel regulators of the DDR with a functional role in B‐cell lymphomagenesis. As a result, we identified SLF2 loss as a biomarker for a subgroup of human DLBCL patients with adverse prognosis and provided direct experimental evidence that SLF2 acts as tumor suppressor. Beyond this, we show that SLF2 loss resulted in loss of several DDR and cell cycle regulators, among them CLSPN. Consequently, we observed an impaired CHK1 axis in the response to DNA damage stress. We propose that SLF2 loss leads to an aberrant SUMOylation pathway, which is in line with the synthetic lethality to a clinically applicable SUMOylation inhibitor we observed.

Functional *in vivo* screening has evolved as a powerful tool to understand and interpret the thousands of genes altered by genetic and non‐genetic mechanisms like transcriptional dysregulation in cancer (Rad *et al*, 2010; Weber *et al*, 2019). We here used a catalog of BCL drivers and show that it is feasible to deploy these data for biologically and clinically relevant aspects in subgroups of cancer patients. We exemplified this approach with the identification of drivers involved in the regulation of the DDR, which is typically activated in response to oncogene activation and is considered a common mechanism to prevent progression of pre‐neoplastic lesions (Bartkova *et al*, 2005). We intentionally used a catalog derived from MYC‐driven B‐cell lymphomas due to the exceptional load of DNA damage caused by activated MYC signaling in these lymphomas (Rohban & Campaner, 2015). By this approach, we identified SLF2 as a regulator of the CHK1 axis. This finding establishes the understanding of the mechanistic impact of SLF2 and is in line with previous studies that described SLF2 as a player of the DDR (Raschle *et al*, 2015; Scott *et al*, 2021). Importantly, our work establishes SLF2 as a highly conserved and actionable tumor suppressor and we propose a treatment strategy for a subgroup of aggressive lymphoma patients with adverse prognosis defined by a low SLF2 state.

Functional forward *in vivo* screenings provide a first level of *in vivo* evidence on the function of candidate genes in tumorigenesis. Considering the tumor‐relevant molecular mechanism downstream of SLF2 loss, we identified loss of CLSPN. Since CHK1 activation by ATR critically depends on the adaptor protein CLSPN, our finding explains the impaired CHK1 activation that we observed in SLF2 deficient cells. We identified a critical role for CLSPN in the phosphorylation of CHK1 in the SLF2 context. At this point, we cannot exclude the possibility that other factors may also contribute to CHK1 phosphorylation. Future work will need to reveal the signaling events that connect SLF2 loss to CLSPN and potentially other factors. CLSPN protein levels are tightly regulated by both transcriptional and post‐translational mechanisms (Smits *et al*, 2019). Our data showing transcriptional or post‐transcriptional regulation of CLSPN suggest that SLF2 is involved in these regulatory circuits.

The ATR‐CHK1 pathway has both pro‐ and anti‐tumoral activities depending on the cellular context and the level of CHK1 activation is critical for malignant transformation (Menoyo *et al*, 2001). Whereas a subtle reduction of CHK1 activity is beneficial for the accumulation of tumor‐promoting mutations and drives tumorigenesis, full loss of CHK1 activity is toxic even for tumor cells due to the excessive and irrepressible level of DNA damage (Bric *et al*, 2009). This concept is empowered by a recent report revealing that deficiency for CLSPN, which act as fine‐tuner of CHK1 activity, resulted in spontaneous B‐cell lymphomagenesis (preprint: Hunter *et al*, 2018). Other than CHK1, the complete loss of the ATM/CHK2 axis is considered tumor‐promoting and the *ATM* locus is frequently affected by deletions of the long arm of chromosome 11 (Choi *et al*, 2016). Importantly, CHK1 is also located on chromosome 11q and this deletion occurs frequently in breast cancer, chronic lymphocytic leukemia, and other lymphoid malignancies (Monni & Knuutila, 2001). It could thereby contribute to tumorigenesis and represent an actionable molecular vulnerability, e.g., for SUMOi treatment. Of note, we also show experimentally that SLF2 loss leads to impaired CHK1 activation in response to DNA damage stress in non‐hematopoietic cancer cells and thus provide a mechanistic framework and identify a molecular vulnerability for a highly conserved feature of aggressive cancers.

Earlier studies established that activation of DNA damage signaling is tightly associated with an alteration of the post‐translation SUMOylation pathway and a subsequent wave of SUMOylation (Cremona *et al*, 2012; Psakhye & Jentsch, 2012). In this cascade, DNA damage‐induced SUMOylation is an integral part of the DDR, and these studies proposed that ablating DNA damage‐induced SUMOylation in checkpoint‐deficient cells might lead to more effective cancer treatment strategies (Cremona *et al*, 2012). This is particularly relevant given the accumulation of DSBs in SLF2‐deficient cells and the increased expression of the SUMO E1 enzyme SAE1 in both SLF2‐ and CLSPN‐deficient cells. We here show that this molecular vulnerability is actionable with pharmacological SUMOi. Importantly, the SUMOylation pathway is regulated on various layers from SUMO conjugation controlled by E1 (SAE1/SAE2), E2 (UBE2I) and E3 ligases to de‐SUMOylation, which is tightly controlled by SUMO isopeptidases (Seeler & Dejean, 2017; Kunz *et al*, 2018). The increase in SAE1 expression and potentially SUMO pathway activity may not automatically result in a high level of SUMOylated target proteins as this pathway is heavily counteracted by SUMO isopeptidases (Kunz *et al*, 2018; Schick *et al*, 2022). Of note, the highly effective SUMOi subasumstat/TAK‐981 is currently undergoing early clinical phase testing in lymphoma and further cancer entities. Several studies have already shown the successful application of SUMOi in a variety of cancers, such as pancreatic cancer and BCL (Hoellein *et al*, 2014; Biederstadt *et al*, 2020) and that subasumstat can act via activation of the immune system (Lightcap *et al*, 2021; Demel *et al*, 2022; Kumar *et al*, 2022) or direct killing of tumor cells (Biederstadt *et al*, 2020; Demel *et al*, 2023). Importantly, we here report SLF2 loss as an actionable biomarker for BCL patients. This work can thus directly inform clinical testing of subasumstat/TAK‐981 and we propose SUMOi as a treatment strategy for a subgroup of DLBCL patients with adverse prognosis.

In addition, we here expand this concept beyond tumors with SLF2 loss, specifically to a with deficient DDR upon pharmacological inhibition of CHK1. Such impairment creates an actionable molecular dependency towards the SUMOylation pathway. Based on our findings we derived a co‐treatment strategy for aggressive human cancers combining SUMOi with inhibitors of the DDR. Previous work demonstrated that checkpoint inhibition strongly alters replication stress‐induced SUMOylation, thus indicating that SUMOylation is particularly critical to limit DSBs under these conditions. Targeting SUMOylation could thus generally sensitize cells for checkpoint inhibitors. This is particularly relevant as many inhibitors of the DDR failed in clinical trials due to severe toxicity (Dent, 2019). The striking synergism of DDR inhibitors and SUMOi could allow lowering the effective dose of DDR inhibitors, thus possibly circumventing or reducing unacceptable side effects of DDR inhibition. In essence, the concept of co‐targeting the evolutionary and across entities highly conserved dependency of SUMOylation and the DDR could be applied to a broad spectrum of aggressive human cancers.

## Materials and Methods

### Animal experiments

All the animal experiments were performed in accordance with Federation of European Laboratory Animal Science Associations (FELASA) guidelines and with the permission of local authority (District Government of Upper Bavaria, Munich, Germany). Mice were fed normal diet, anesthetized by isoflurane administration and euthanized by cervical dislocation. The night/light cycle was adjusted to 14 h lights on and 10 h lights off (dark period set between 8.00 pm and 6.00 am). The Animal Core Facility has regularly tested the health status of mice. *Eμ‐myc* mice (002728) were purchased from Jackson Laboratory and crossed with *Rosa26* ^*Cas9*^ mice to generate *Eμ‐myc; Rosa26* ^*Cas9*^ hematopoietic stem and progenitor cells (HSPCs) derived from fetal livers at E14.5. Single guide RNA (sgRNA) sequence was designed and selected with CHOPCHOP (http://chopchop.cbu.uib.no/) sgRNA design resource, and cloned into the pLKO5.sgRNA.EFS.GFP (Addgene #57822) backbone. Transduction‐transplantation experiments have been performed as described before (Weber *et al*, 2019; Schick *et al*, 2022). The transduction efficacy was between 15 and 25% and 2.5 × 10^5^ eGFP‐positive HSPCs and 2 × 10^5^ CD45.1 bone marrow helper cells were transplanted into lethally irradiated (8.5 Gy) recipient mice. Female C57Bl6/J mice aged 6–8 weeks, which were purchased from Charles River, were used for the transplantation experiments. Mice were examined twice a week and sacrificed as soon as lymph nodes were well palpable (5 mm diameter) or any of the approved thresholds were reached.

### *In vivo* co‐treatment experiments

Animal experiments were conducted as previously described (Stroh *et al*, 2022). Therefore, 1.5 × 10^7^ DLBCL cells were resuspended in serum‐free media, mixed with Matrigel Basement Membrane Matrix (Corning) at 1:1 ratio and injected subcutaneously into the upper right flanks of female NOD.CB17/PrkdcSCID/Rj mice 10 weeks of age (Janvier labs). Once mice showed tumor engraftment, they were randomly assigned to receive subasumstat (25 mg/kg) i.v., rabusertib (90 mg/kg) i.p., a combination of both or vehicle control twice a week. The tumor growth was assessed by caliper measurements. Mice were housed under specific pathogen‐free conditions. The animal experiments were conducted in accordance with the local ethical guidelines and approved by the regional authorities (District Government of Upper Bavaria, application no.: ROB‐55.2‐2532.Vet_02‐17‐230/ROB‐55.2‐2532.Vet_02‐20‐46; Government of Berlin, application no.: G0006‐21). For the syngeneic mouse model, the female CD45.1 mice (6 weeks old) were purchased from the Charles River Laboratories (Sulzfeld, Germany), and maintained in the animal facility at Charité ‐ Universitätsmedizin Berlin. 4 × 10^6^ of primary *Eμ‐Myc* lymphoma cells, which were harvested from the enlarged lymph nodes of sick *Eμ‐Myc* mice, were injected into each of the CD45.1 mice and watching for tumor onset. Animal husbandry and care at Charité ‐ Universitätsmedizin Berlin were performed under the same conditions as indicated in the Animal Experiments section (diet; day/night cycle).

### Histology

Mouse lymph nodes were fixed in 10% neutral‐buffered formalin solution for 48 h, dehydrated under standard conditions (Leica ASP300S, Wetzlar, Germany), and embedded in paraffin. Serial 2 μm sections prepared with a rotary microtome (HM355S, ThermoFisher Scientific, Waltham, USA) were collected and subjected to histological and immunohistochemical analysis with antibodies detecting B220 and CD3 (BD Biosciences).

### Analysis of CRISPR/Cas9 target regions

Genomic DNA from the infiltrated lymph nodes was isolated using the Qiagen Blood and Tissue kit. PCR amplification of targeted loci was carried out with Q5® Hot Start High Fidelity 2× Master Mix. The PCR products were analyzed using Engen T7 Endonuclease I according to the manufactory's protocol.

### Cell culture

NIH‐3T3, HEK293T, U‐2‐OS, MiaPaCa2, A549, and Phoenix‐Eco cells were purchased from LGC Standards/American Type Culture Collection (Manassas, VA, USA) were cultured in DMEM with 10% FCS. Human DLBCL cell lines were cultured in RPMI‐1640, IMDM, or alpha MED medium supplemented with 10% FCS. *Eμ‐myc* lymphoma‐derived cell lines were cultured in RPMI‐1640 medium with 20% FCS, 2 mM L‐Glutamine, 1% of non‐essential amino acid and 0.1% of ß‐Mercaptoethanol. SU‐DHL‐5, SU‐DHL‐6, OCI‐Ly1, and OCI‐Ly19 cells were purchased from DSMZ (Leibniz institute DMSZ‐German Collection of Microorganisms and Cell Culture). *Eμ‐myc* lymphoma cell lines were established from single‐cell tumor suspension by our lab as described (Hoellein *et al*, 2014). Primary murine lymphoma cells were harvested either from *Eμ‐myc;Rosa26* ^*cas9*^ or *Eμ‐myc* mice lymphoma and co‐cultured with irradiated NIH‐3 T3 cells in BCM medium (45% of DMEM, 45% of IMDM, 10% FCS, 4 mM of L‐Glutamine, 2.5 × 10^−5^ M 2‐mercaptoethanol, 100 U/ml penicillin, 100 μg/ml streptomycin). All the cell lines were recently authenticated and tested for mycoplasma contamination regularly.

### Plasmids and viral infection

Specific short hairpin RNA (shRNA) sequences targeting murine *Slf2* were extracted from the Sigma MISSION Library and modified to fit the miR30 hairpin expression system (TRCN0000241755_Modified sequence: TGCTGTTGACAGTGAGCGCTCACCTTATAGTCCAGTATTTTAGTGAAGCCACAGATGTAAAATACTGGACTATAAGGTGAATGCCTACTGCCTCGGA; TRCN0000241756_Modified sequence: TGCTGTTGACAGTGAGCGCTAGATACGAAGAGCTATATTTTAGTGAAGCCACAGATGTAAAATATAGCTCTTCGTATCTATTGCCTACTGCCTCGGA). The sequences were synthesized by Eurofins and cloned into the MSCV‐LTRmiR30‐SV40GFP‐PURO (LMP) vector. For the generation of ecotropic retroviral particles, Phoenix‐Eco cells were transfected with the indicated retroviral plasmids. Virus supernatants were collected 48 h after transfection and used to transduce the indicated cell lines in the presence of 1 μg/ml polybrene (Millipore). Suspension cells were transduced using spin‐transduction at 216 *g* for 1 h at 32°C.

### Small interfering RNAs (siRNAs)‐based generation of human and murine SLF2 depletion

For depletion of human SLF2 in HEK293T cells and murine *SLF2* in NIH3T3 cells, the pre‐designed siRNA sequences (siRNA ID#: s31333, n433558) were purchased from ThermoFisher Scientific. Thirty picomole of the targeting and non‐targeting sequences were transfected by using Invitrogen Lipofectamine RNAiMAX Transfection Reagent (ThermoFisher Scientific, 13778150‐1.5 ml) and Opti‐MEM™, Reduced Serum Medium (Gibco™, 31985062) for 72 h. The knockdown efficiency was validated by Western blotting.

### CRISPR/Cas9‐based generation of SLF2 and CLSPN cell lines

For depletion of SLF2 in U‐2‐OS and SU‐DHL‐5 cell lines, a fragment ranging from exon 3 (sgRNA sequence: TAATACGACTCACTATAGGAGTAGATTGTCTATCACTGTTTTAGAGCTAGAAATAGC) to exon 5 (sgRNA sequence: TAATACGACTCACTATAGGACCTTTGCGCTCAGAATAGTTTTAGAGCTAGAAATAGC) of the SLF2 open reading frame was removed by CRISPR/Cas9 gene editing. For depletion of CLSPN in SU‐DHL‐5 cells, two sgRNAs flanking exon1 were applied (sgRNA1 sequence: taatacgactcactataGGCCAGAGGCGCTGCGTGATgttttagagctagaaatagc; sgRNA2 sequence: taatacgactcactataGGGCCACGGAGCCCGAAGCGgttttagagctagaaatagc). For this purpose, 150.000 cells were transfected with 500 ng of each of the sgRNAs and 1 μg of Cas9 protein (PNA Bio) with a Neon Transfection System (Thermo Fisher/Invitrogen) (parameters: 1,450 V; 10 ms; 4 pulses). The cleavage efficacy was tested 72 h following transfection with Terra™ PCR Direct Card Kit. Cells were then seeded to single cells by serial dilution. Cell clones were screened for efficient gene editing and selected clones were analyzed for SLF2 protein expression by immunoblot analysis.

### Chemicals

Puromycin Dihydrochloride (A1113803) was purchased from ThermoFisher Scientific. A concentration range from 0.5 to 5 μg/ml was used for cell selection. Hydroxyurea (HU, H8627‐5G) and aphidicolin from Nigrospora sphaerica (A0781‐1MG) were purchased from SIGMA. Doxorubicin, AZD7762, prexasertib, and rabusertib were purchased from Selleck, USA. The larger batch of rabusertib for the *in vivo* usage was purchased from TargetMol Chemicals Inc., USA. MG132 (474790) was purchased from Merck Sigma‐Aldrich. TAK‐243 (S8341) was purchased from Selleck. The concentration of doxorubicin used to induce checkpoint activation is 0.5 μM. Mafosphamide (sc‐211761) was purchased from Santa Cruz. SUMO inhibitors (TAK‐981 and ML‐093) were purchased from MedChemExpress or were provided by Millennium Pharmaceuticals, Inc., a wholly‐owned subsidiary of Takeda Pharmaceutical Company Limited. All the concentrations of the inhibitors mentioned above used in the experiments are indicated in the figures.

### Immunocytochemistry

U‐2‐OS cells were cultured overnight on the slides coated with collagen type I (Sigma, C‐9791), and fixed with ice‐cold methanol (Carl Roth). Afterwards, the cells were incubated with 0.3% Triton X‐100 (Sigma, T‐8787) and blocked with 5% goat serum (Invitrogen, 31872) in PBS. The cells were stained with primary anti‐gamma H2A.X (phosphor S139) antibody (Abcam, ab11174) and secondary Goat anti‐Rabbit IgG (H+L) Highly Cross‐Absorbed Secondary Antibody, Alexa Fluor Plus 594 (Invitrogen, A32740). The slides were mounted with Immunoselect Antifading Mounting Medium DAPI (Dianova, SCR‐038448), and imaged on a Zeiss LSM 780 confocal microscope (Carl Zeiss).

### BrdU assay

Cells were seeded and cultured for 2–3 h before adding 10 μl of 1 mM BrdU per ml to culture medium for labeling. After harvesting, the cells were fixed and permeabilized according to the manufacturer's instructions of the BD Pharmingen™ APC BrdU Flow Kit (Component of 552598 or 557892). Moreover, the cells were stained by following the manufacturer's instructions of the same kit and the cell cycle were analyzed by flow cytometry.

### Gene set enrichment analysis and pathway enrichment analysis/bioinformatic analysis

Gene expression data from human DLBCL samples and murine *Eμ‐Myc* lymphomas were obtained from the publicly available gene expression omnibus (GEO) database with accession numbers: GSE7897 (mouse *Eμ‐Myc* lymphoma; Affymetrix Mouse Genome 430 2.0 Array), GSE4475 (human DLBCL, Affymetrix Human Genome U133A Array), GSE2350 (human DLBCL, Affymetrix Human Genome U95A/U95 Version 2 Array) (Basso *et al*, 2005; Hummel *et al*, 2006; Mori *et al*, 2008). Affymetrix array CEL files were processed using Expression Console software (Affymetrix). Data were normalized via the robust multi‐array algorithm (RMA), transformed via log_2_ and the probes collapsed. Indicated groups from the used datasets were analyzed using GeneTrail3.0 software (Gerstner *et al*, 2020) and indicated signatures from the Molecular Signature Database (MSigDb) (Liberzon *et al*, 2015). Results were illustrated using volcano‐ or GSEA plots (GraphPad Prism v9).

### RNA isolation and RNA‐sequencing

Total RNA was isolated from murine lymphoma cells and SU‐DHL‐5 cells by using RNeasy Mini Kit (Qiagen) according to the manufacturer's protocol. RNA‐sequencing was performed as recently described (Doffo *et al*, 2022). Briefly, after library generation quality (fragment size) and quantity were analyzed (Tape Station, Agilent). Sublibraries were pooled equimolarily and were sequenced by Illumina HiSeq2500 for 150 bp in paired end fashion. Quality of raw reads was checked and adapters trimmed using Trimmomatic (Bolger *et al*, 2014). Trimmed read files were aligned to GRCh38 using HISAT2 (Kim *et al*, 2015). Differential gene expression analysis was carried out with DEseq2 (Love *et al*, 2014). Normalized logarithmic data were used for subsequent gene set enrichment analysis.

### Flow cytometry

Cells were stained with 4′,6‐diamidino‐2‐phenylindole (DAPI) to assess cell viability and propidium iodide (PI) for cell‐cycle analysis. To analyze apoptosis, cells were stained with DAPI or LIVE/DEAD™ Fixable Aqua Dead Cell Stain (Invitrogen, L34957)/Annexin‐V staining (Alexa Fluor^®^ 647 Annexin V, BioLegend, 640912). Data were acquired using a CytoFLEX S Flow Cytometer (Beckman Coulter) and analyzed with FlowJo™ Version 10.6.0 software.

### Cell viability assay

Cells from different cancer entities were treated with increasing concentrations of SUMOi combined either with prexasertib or rabusertib and cell viability was measured by CellTiter‐Glo^®^ 2.0 Cell Viability Assay (Promega, G9243) according to the manufacturer's instructions. The luminescence signal was collected by CentroPRO LB 962 Microplate Luminometer (Berthold Technologies GmbH & Co. KG). The ZIP synergy scores were calculated by using SynergyFinder (Version 2.0 or version 3.0) software (website link: https://synergyfinder.fimm.fi/synergy/20210914145134133998/).

### Quantitative RT–PCR

RNA isolation was implemented by using RNeasy Mini Kit (QIAGEN, 74106). qPCR was performed using Luna Universal One‐Step RT‐qPCR Kit (NEB, E3005X) and Ct values were measured by a TaqMan cycler (Applied Biosystems). The gene expression level was analyzed by using the ΔΔCt method with control samples set as 1. Primer sequences: *CLSPN* (fw: AAGACAGTGATTCCGAAACAGAG, rv: TGCGCTTCAAGATTTTCC TGA), *GAPDH* (fw: GGTATCGTGGAAGGACTCATGAC, rv: ATGCCAGTGAGCTTCCCGTT CAG).

### Immunoblot analysis

Protein extracts were prepared by incubating cell pellets in RIPA buffer and were fractioned on SDS–PAGE gels. Protein lysates were transferred to PVDF membranes (Sigma) and incubated with specific antibodies and developed with Chemostar PC ECL & Fluorescence Imager (Insta Science Imaging, Göttigen, Germany). A list of antibodies used can be found in the supplemental table. All the quantification of the blots was analyzed by ImageJ (NIH).

### Immunoblotting antibodies

**Table:** 

Protein	Company	Product#	Dilution
SLF2	Abcam	Ab122480	1:1,500
p‐CHK1 (Ser345)	Cell Signaling	2341S	1:1,000
CHK1	Cell Signaling	2360S	1:1,000
p‐CHK2 (Thr68)	Cell Signaling	2661S	1:1,000
CHK2	Cell Signaling	6334S	1:1,000
CLSPN	Cell Signaling	2800S	1:1,000
AURKA	Cell Signaling	14475	1:2,000
AURKB	Cell Signaling	3094	1:2,000
PTTG‐1	Santa Cruz	sc‐56207	1:1,000
FANCD2	Santa Cruz	sc‐20022	1:1,000
SMC5	Invitrogen	PA5‐63037	1:3,000
UbP4D1	Santa Cruz	sc‐8017	1:1,000
SAE1	Abcam	ab185949	1:1,000
SAE2	Abcam	ab185955	1:1,000
ß‐Actin	SigmaAldrich	A1978	1:5,000
ß‐Tubulin	DSHB	E7	0.4 μg/ml
Vinculin	Cell Signaling	13901S	1:1,000
Anti‐rabbit	GE Healthcare	NA934V	1:5,000
Anti‐mouse	Cell Signaling	7076S	1:5,000

### Sample preparation for LC–MS/MS

For whole cell proteome analysis, cells were lysed, reduced, and alkylated in SDS‐lysis Buffer (2% SDS, 50 mM Tris pH 8.5, 10 mM TCEP, 40 mM CAA) complemented with protease inhibitor tablet. Cellular lysates were subsequently boiled, sonicated, and subjected to methanol‐chloroform precipitation. The resulting dried pellet was resuspended in urea digestion buffer (8 M urea, 50 mM Tris pH 8.2) and protein concentration was measured by BCA assay (Pierce™ BCA Protein Assay Kit). Fifty microgram protein was digested by Trypsin (enzymes to protein ratio 1:100) and Lys‐C (enzymes to protein ratio 1:50) overnight at 37°C in 1 M urea, 50 mM Tris pH 8.5. Proteolytic cleavage was stopped by TFA (final concentration 1%) and peptides were subsequently desalted using tC18 Sep‐Pak cartridges (Waters, 50 mg). Subsequently, digested peptides were dissolved in 200 mM EPPS pH 8.2, 10% ACN buffer and peptide concentration was measured by micro BCA assay (Micro BCA™ Protein Assay Kit). Ten microgram of digested peptides were finally labeled (peptides to TMT ratio 1:2) with TMT6‐plex reagents (ThermoFisher Scientific) for 1 h at room temperature. Quenching of the labelling reaction was performed by hydroxylamine at a final concentration of 0.5% and equal amounts of TMT‐labeled samples were pooled followed by cleaning up using tC18 Sep‐Pak cartridges (Waters, 50 mg). For chromatin proteome analysis, enrichment of chromatin‐associated proteins was performed as described in Kustatscher *et al* (2014). Fifty microgram of protein was subjected to Filter‐Aided Sample Preparation as described in Wiśniewski (Kustatscher *et al*, 2014; Wisniewski, 2018). Digestion with Trypsin and Lys‐C and downstream labelling procedure was performed as described before.

### High pH micro‐flow fractionation

Peptides were fractionated using high‐pH liquid chromatography on a micro‐flow HPLC (Dionex U3000 RSLC, Thermo Scientific). Forty‐five microgram of pooled and purified TMT labeled peptides resuspended in Solvent A (5 mM ammonium‐bicarbonate, 5% ACN) were separated on a C18 column (XSelect CSH, 1 mm × 150 mm, 3.5 μm particle size; Waters) using a multistep gradient from 3 to 60% Solvent B (100% ACN) over 65 min at a flow rate of 30 μl/min. Eluting peptides were collected every 43 s from minute 2 for 69 min into a total of 96 fractions, which were cross‐concatenated into 24 or 16 (for chromatin) fractions. Pooled fractions were dried in a vacuum concentrator and resuspended in 3% ACN, 0.1% TFA for LC–MS analysis.

### Mass spectrometry

Tryptic peptides were analyzed on an Orbitrap Lumos coupled to an easy nLC 1200 (ThermoFisher Scientific) using a 35 cm long, 75 μm ID fused‐silica column packed in house with 1.9 μm C18 particles (Reprosil pur, Dr. Maisch), and kept at 50°C using an integrated column oven (Sonation). A synchronous precursor selection (SPS) multi‐notch MS3 method was used in order to minimize ratio compression as previously described (McAlister *et al*, 2014). Assuming equal amounts in each fraction, 500 ng of peptides were eluted by a non‐linear gradient from 4 to 32% ACN over 90 min followed by a step‐wise increase to 75% ACN in 6 min which was held for another 9 min. Full scan MS spectra (350–1,400 *m/z*) were acquired with a resolution of 120,000 at *m/z* 200, maximum injection time of 100 ms and AGC target value of 4 × 10^5^. The most intense precursors with a charge state between 2 and 6 per full scan were selected for fragmentation (“Top Speed” with a cycle time of 1.5 s) and isolated with a quadrupole isolation window of 0.7 Th. MS2 scans were performed in the Ion trap (Turbo) using a maximum injection time of 50 ms, AGC target value of 1.5 × 10^4^ and fragmented using CID with a normalized collision energy (NCE) of 35%. SPS‐MS3 scans for quantification were performed on the 10 most intense MS2 fragment ions with an isolation window of 0.7 Th (MS) and 2 *m/z* (MS2). Ions were fragmented using HCD with an NCE of 65% and analyzed in the Orbitrap with a resolution of 50,000 at *m/z* 200, scan range of 110–500 *m/z*, AGC target value of 1.5 × 10^5^ and a maximum injection time of 86 ms. Repeated sequencing of already acquired precursors was limited by setting a dynamic exclusion of 45 s and 7 ppm and advanced peak determination was deactivated.

### Raw data analysis and statistical significance evaluation

Raw data were analyzed with Proteome Discoverer 2.4 (ThermoFisher Scientific). Acquired MS2‐spectra were searched against the human trypsin digested proteome (20,531 sequences) and a collection of common contaminants (from MaxQuant's “contaminants.fasta”) using SequestHT, allowing a precursor mass tolerance of 7 ppm and a fragment mass tolerance of 0.5 Da after recalibration of mass errors using the Spectra RC‐node applying default settings. In addition to standard dynamic (Oxidation on methionines and acetylation of protein N‐termini) and static (Carbamidomethylation on cysteins) modifications, TMT‐labelling of N‐termini and lysines were set as static modifications. False discovery rates were controlled using Percolator (< 1% FDR on PSM level). Only PSMs with a signal‐to‐noise above 10 and a co‐isolation below 50% derived from unique peptides we are used for protein quantification after total intensity normalization. Peptide groups file was exported into .txt file and subsequent statistical analysis was done with the Perseus software (version 1.6.15.0). Log_2_ values of all the normalized abundances were calculated. Using the histogram analysis function of the software, the normal distribution of the abundance values was visually checked. Good correlation of the experimental replicates was assured by multi‐scatterplot analysis. Samples were then grouped into triplicates and a Student's *t*‐test was performed with randomization of 250 and permutation based FDR 0.05. Then the datasets were exported and used for further analysis in Microsoft Excel. Significant enrichment was defined in Excel based on the *P*‐value and the Student's *t*‐test difference applying the following criteria: −log_10_ *P*‐value > 1.34 and log_2_ ratio ≥ 1 or ≤ −1. Visual representation of data in volcano plots was done using the online portal https://huygens.science.uva.nl/VolcaNoseR/.

### Statistical analysis

All the statistical analyses were performed using GraphPad Prism Version 9.0 (GraphPad Software, La Jolla, CA). The error bars shown in the figures represent standard deviation (SD) unless specified otherwise. In each experiment, the used statistical analysis methods are indicated in the figure legends. Results with a *P*‐value of less than 0.05 were considered significant and indicated in the figures. For *in vivo* experiments, mice were censored from analyses when sacrificed for non‐tumor reasons. For all experiments other than *in vivo* experiments, samples were allocated into experimental groups in a random fashion. No blinding was performed.

## Author contributions

**Le Zhang:** Conceptualization; data curation; formal analysis; visualization; writing – original draft. **Matthias Wirth:** Investigation; visualization; methodology; writing – original draft; writing – review and editing. **Upayan Patra:** Investigation; methodology. **Jacob Stroh:** Formal analysis; investigation; methodology. **Konstandina Isaakidis:** Investigation; methodology. **Leonie Rieger:** Investigation; methodology. **Susanne Kossatz:** Investigation; methodology. **Maja Milanovic:** Methodology. **Chuanbing Zang:** Investigation. **Uta Demel:** Investigation; writing – original draft; writing – review and editing. **Jan Keiten‐Schmitz:** Investigation. **Kristina Wagner:** Investigation. **Katja Steiger:** Investigation. **Roland Rad:** Investigation; methodology. **Florian Bassermann:** Investigation; methodology; writing – original draft; writing – review and editing. **Stefan Müller:** Supervision; investigation; methodology; writing – original draft; writing – review and editing. **Ulrich Keller:** Conceptualization; supervision; funding acquisition; investigation; methodology; writing – original draft; writing – review and editing. **Markus Schick:** Conceptualization; formal analysis; funding acquisition; investigation; visualization; methodology; writing – original draft; writing – review and editing.

## Disclosure and competing interests statement

UK has received reimbursement for advisory board function, speaker honorary and travel support from Takeda for content unrelated to this manuscript. The other authors report no conflict of interest.

## Supporting information



Appendix

Table EV1

Dataset EV1

Dataset EV2

Dataset EV3

Source Data for Appendix

Source Data for Figure 1

Source Data for Figure 4

Source Data for Figure 5

Source Data for Figure 6

Source Data for Figure 7

## Data Availability

The transcriptome data generated in this study have been deposited at the EBI European Nucleotide Archive under accession PRJEB47681 (https://www.ncbi.nlm.nih.gov/bioproject/PRJEB47681). The mass spectrometry proteomics data generated in this study have been deposited in the ProteomeXchange Consortium via the PRIDE partner repository (Deutsch *et al*, 2017; Perez‐Riverol *et al*, 2019) with the dataset identifier PXD041834 (http://www.ebi.ac.uk/pride/archive/projects/PXD041834) and PXD041851 (http://www.ebi.ac.uk/pride/archive/projects/PXD041851).
