# Supplementary material for: Actionable loss of SLF2 drives B‐cell lymphomagenesis and impairs the DNA damage response
Source: EMBO Mol Med. 2023 Jul 24;15(9):e16431. doi: 10.15252/emmm.202216431 (PMC10493575; doi:10.15252/emmm.202216431)
Supplement: Supplementary file 1 — Appendix [file EMMM-15-e16431-s003.pdf]

APPENDIX

Actionable loss of SLF2 drives B-cell lymphomagenesis and impairs the DNA damage response

Zhang et al.

TABLE OF CONTENT

Appendix Figure S1	Page 3
Appendix Figure S2	Page 4
Appendix Figure S3	Page 5
Appendix Figure S4	Page 6
Appendix Figure S5	Page 7
Appendix Figure S6	Page 8
Appendix Figure S7	Page 9
Appendix Figure S8	Page 10
Appendix Figure S9	Page 11
Appendix Figure S10	Page 12
Appendix Figure S11	Page 13
Appendix Figure S12	Page 14
Appendix Figure S13	Page 15
Appendix Figure S14	Page 16

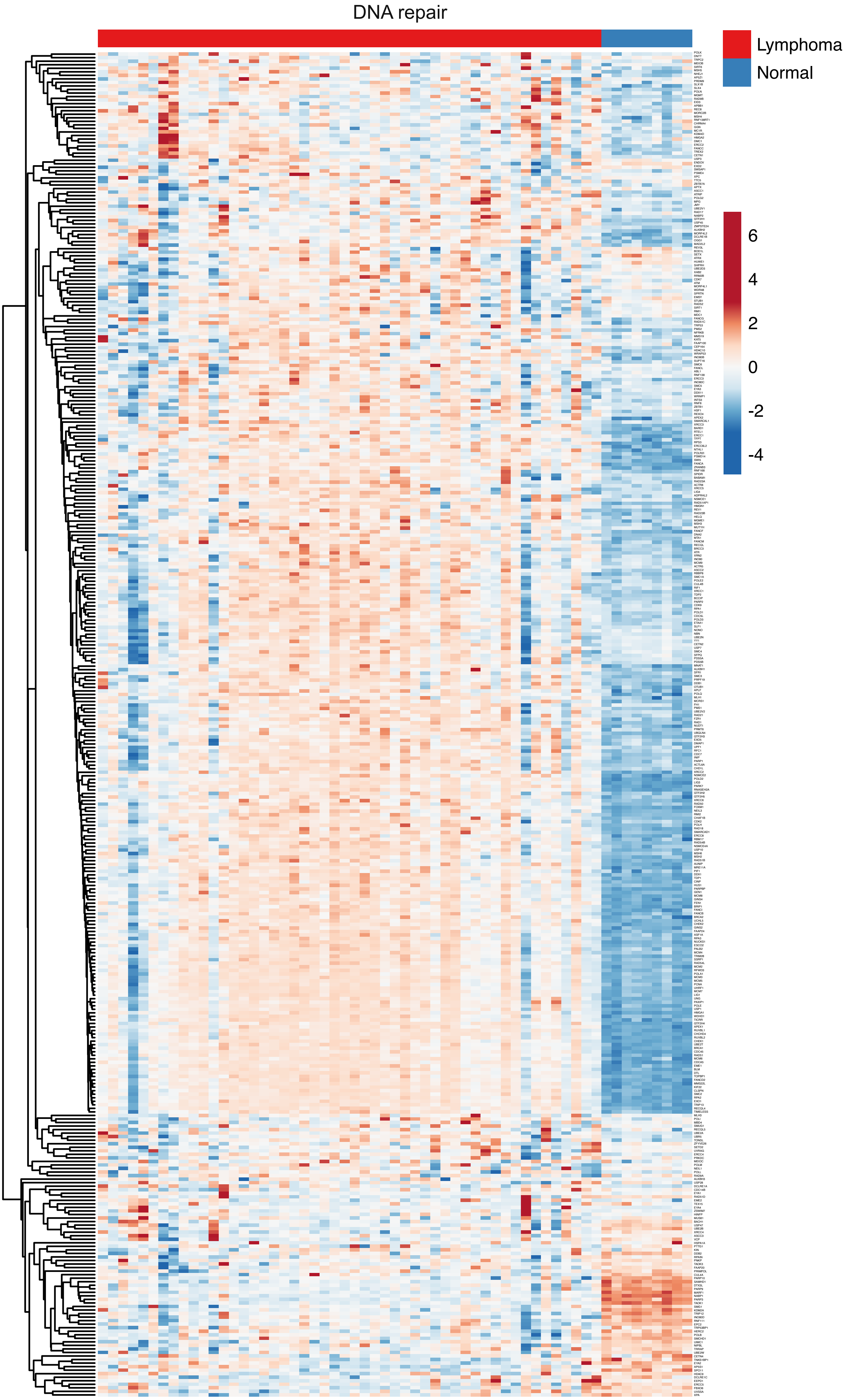

Appendix Figure S1. Analysis of publicly available transcriptome data derived from Eμ-myc lymphomas (n=50) and wildtype B-cells (n=10) showing all genes of the indicated gene set. Assessed was GSE7897.

Cellular Response to DNA damage stimulus

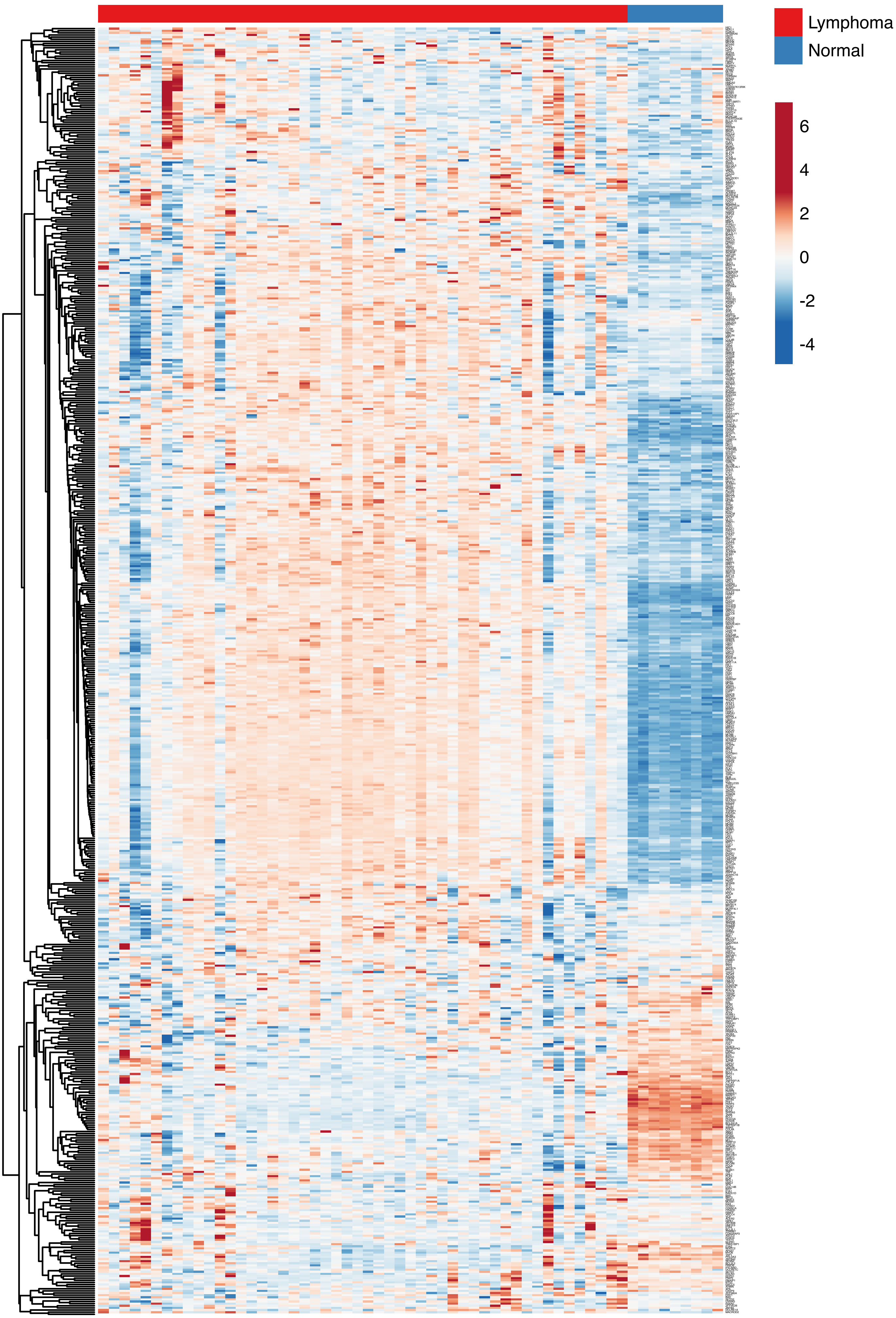

Appendix Figure S2. Analysis of publicly available transcriptome data derived from Eμ-myc lymphomas (n=50) and wildtype B-cells (n=10) showing all genes of the indicated gene set. Assessed was GSE7897.

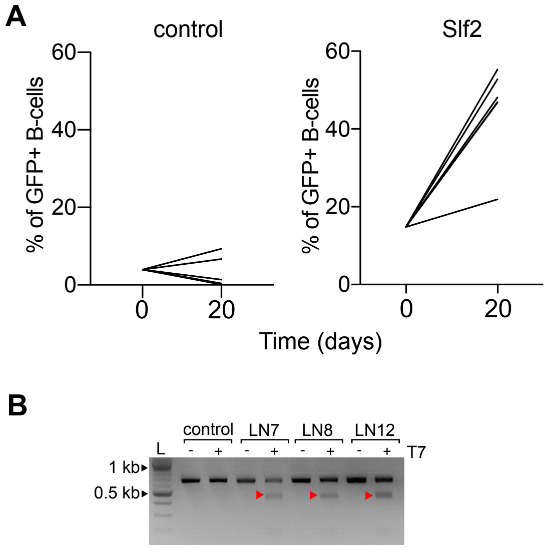

Appendix Figure S3. (A) Enrichment of control-sgRNA (n=5) and Slf2-sgRNA (n=6) transduced HSPCs over time. (B) In vitro T7 nuclease assay showing cutting efficacies of the Slf2-sgRNA in lymphomas from in vivo validation experiment described in Figure 2E.

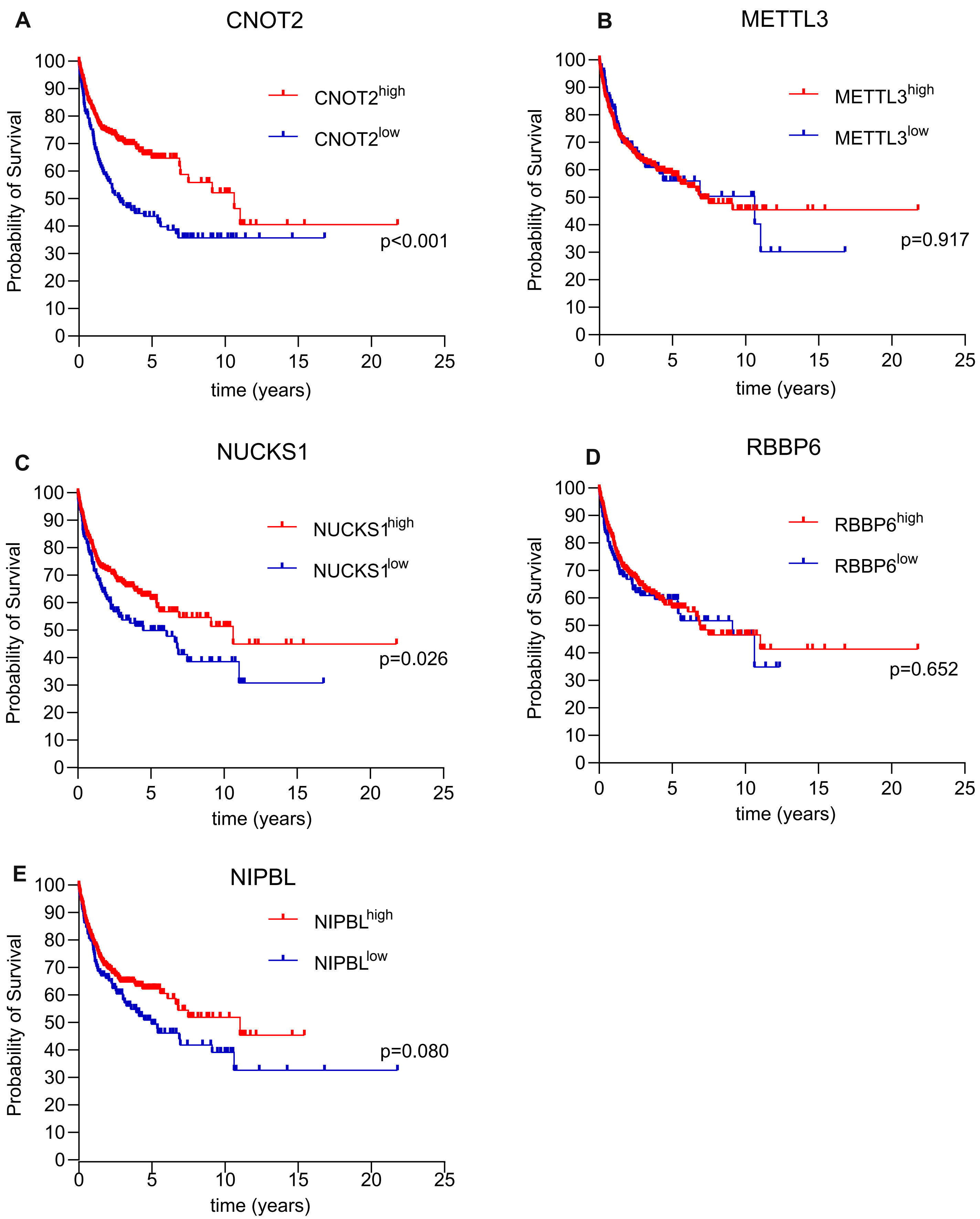

Appendix Figure S4. (A-E) Kaplan-Meier overall survival curves of the indicated cohorts of DLBCL patients (Lenz, NEJM). DLBCL patients were classified according to mRNA expression of the indicated genes. P-value was determined by log-rank (Mantel-Cox) test.

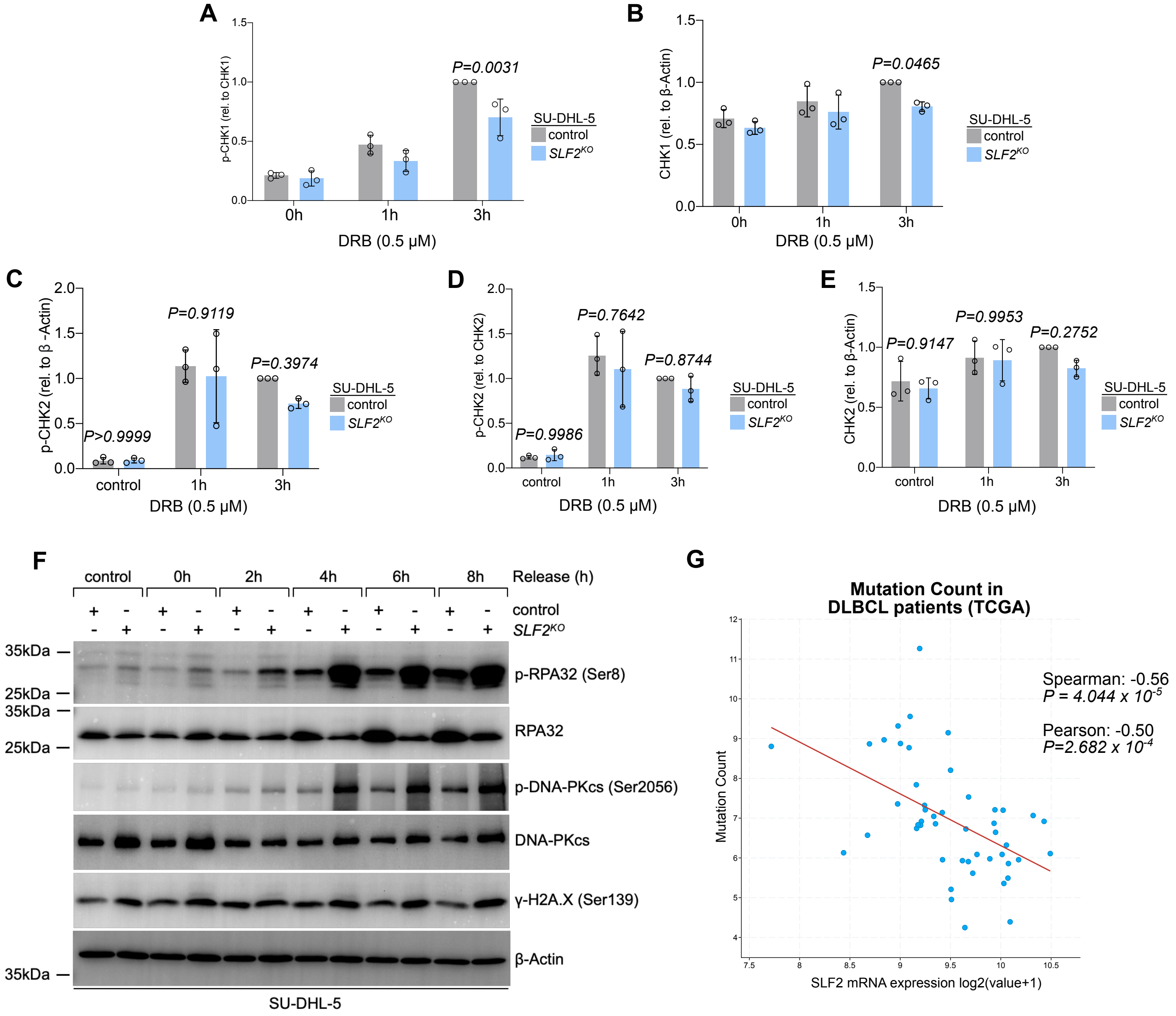

Appendix Figure S5. (A) Quantification of the p-CHK1 western blots from Figure 4E. Protein expression of p-CHK1 was normalized to CHK1 protein expression. p-CHK1 expression in SU-DHL-5 control cells at 3 hours doxorubicin treatment was arbitrarily set to 1. Each dot represents a biological replicate. *P-value* determined by ANOVA; Šídák's multiple comparisons test. (B) Quantification of the CHK1 western blots from Figure 4E. Protein expression of CHK1 was normalized to β-Actin protein expression. CHK1 expression in SU-DHL-5 control cells at 3 hours doxorubicin treatment was arbitrarily set to 1. Each dot represents a biological replicate. *P-value* determined by ANOVA; Šídák's multiple comparisons test. (C) Quantification of the p-CHK2 western blots from Figure 4E. Protein expression of p-CHK2 was normalized to β-Actin protein expression. p-CHK2 expression in SU-DHL-5 control cells at 3 hours doxorubicin treatment was arbitrarily set to 1. Each dot represents a biological replicate. *P-value* determined by ANOVA; Šídák's multiple comparisons test. (D) Quantification of the p-CHK2 western blots from Figure 4E. Protein expression of p-CHK2 was normalized to CHK2 protein expression. p-CHK2 expression in SU-DHL-5 control cells at 3 hours doxorubicin treatment was arbitrarily set to 1. Each dot represents a biological replicate. *P-value* determined by ANOVA; Šídák's multiple comparisons test. (E) Quantification of the CHK2 western blots from Figure 4E. Protein expression of CHK2 was normalized to β-Actin protein expression. CHK2 expression in SU-DHL-5 control cells at 3 hours doxorubicin treatment was arbitrarily set to 1. Each dot represents a biological replicate. *P-value* determined by ANOVA; Šídák's multiple comparisons test. (F) Immunoblot analysis of indicated proteins in SU-DHL-5 control and CRISPR/Cas9 mediated *SLF2^{KO}* cell lines. SU-DHL-5 control and *SLF2^{KO}* cells we treated for 30 minutes with 1 µM doxorubicin (DRB). DRB was then washed out and the cells were for indicated timepoints before harvest for immunoblot analysis. (G) Correlation analysis of *SLF2* mRNA and number of mutations derived from https://www.cbioportal.org/ (Cohort: Lymphoid Neoplasm Diffuse Large B-cell Lymphoma (TCGA, Firehose Legacy).

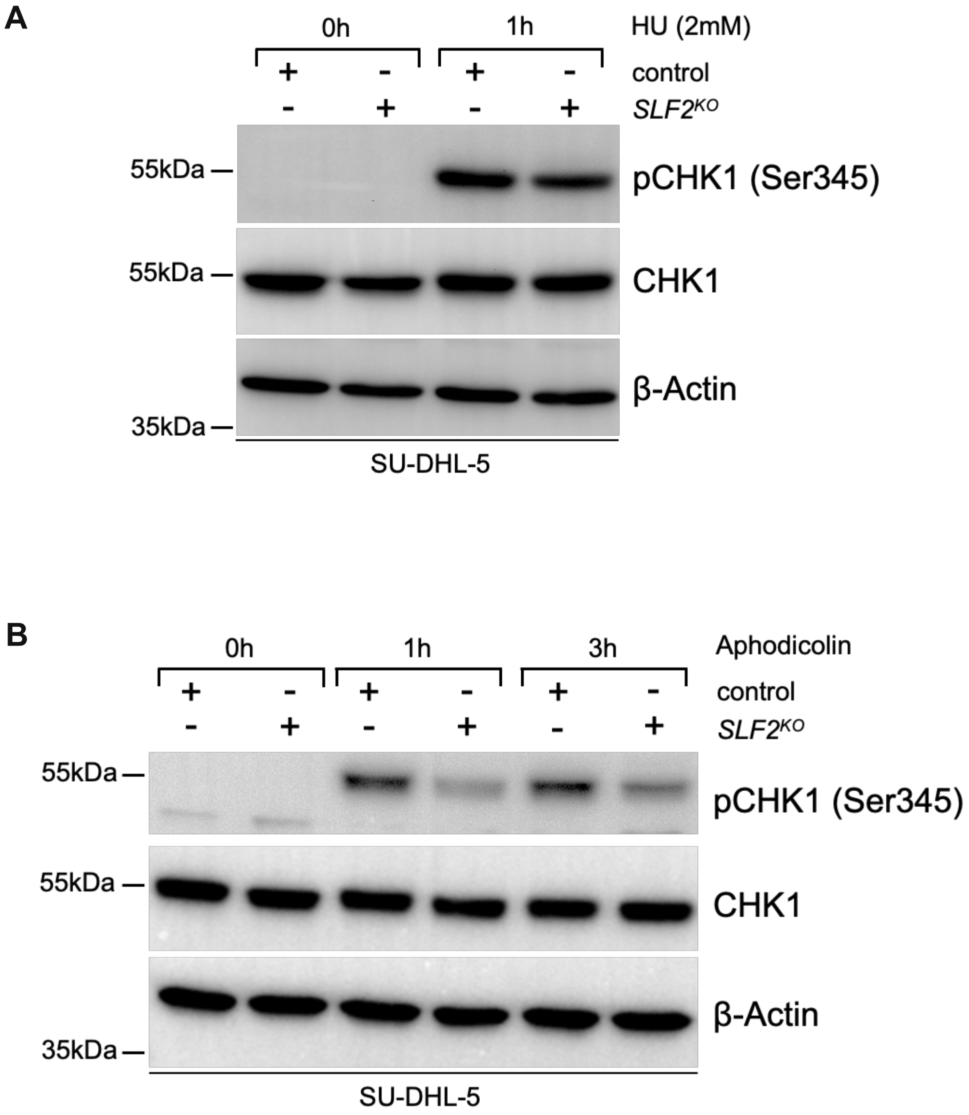

Appendix Figure S6. (A) Immunoblot analysis of indicated proteins in SU-DHL-5 control and CRISPR/Cas9 mediated *SLF2*^{KO} cell lines treated with HU (2 mM) for indicated time points. (B) Immunoblot analysis of indicated proteins in SU-DHL-5 control and CRISPR/Cas9 mediated *SLF2*^{KO} cell lines treated with Aphidicolin (2 µg/ml) for indicated time points.

A

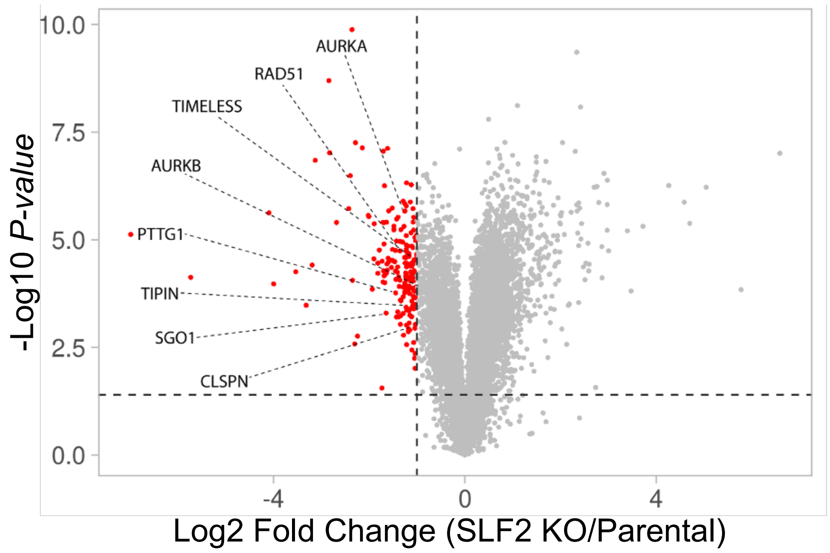

Appendix Figure S7. (A) Volcano plot depicting significantly depleted proteins (red) in whole cell proteome of *SLF2*^{KO} SU-DHL-5 cells compared to the corresponding parental cell line; proteins belonging to the DDR, cell cycle regulation, and chromosome segregation are labelled.

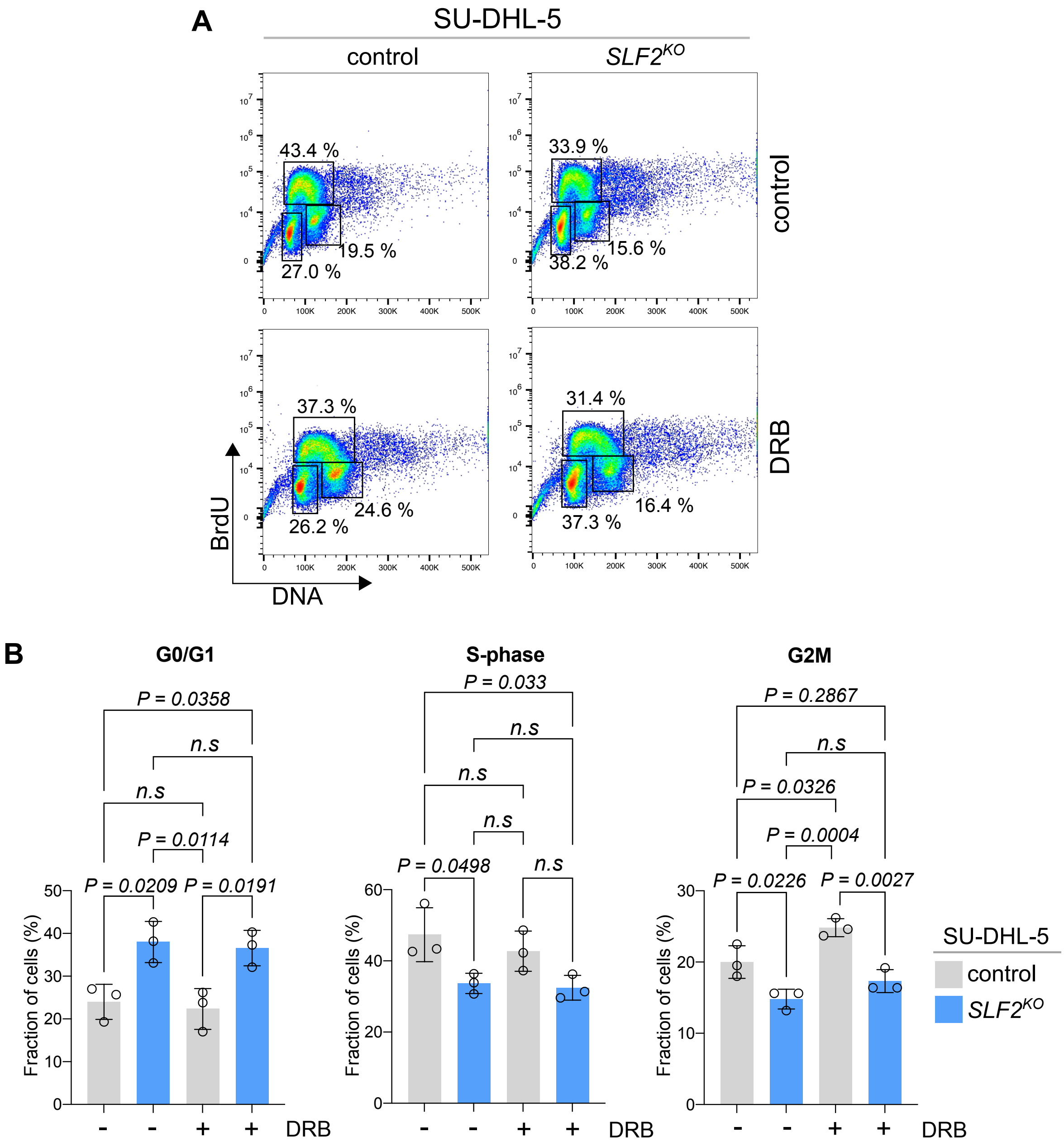

Appendix Figure S8. (A) Representative histograms of the flow cytometry BrdU cell cycle profiling experiment of SU-DHL-5 control and *SLF2*^{KO} cells treated with or without DRB (0.5 µM) for 3 hours. (B) Quantification of flow cytometry results for BrdU cell cycle analysis described in Figure S8A. *P-value* was determined by ANOVA; Tukey's post hoc test. Error bars represent SD from three independent experiments.

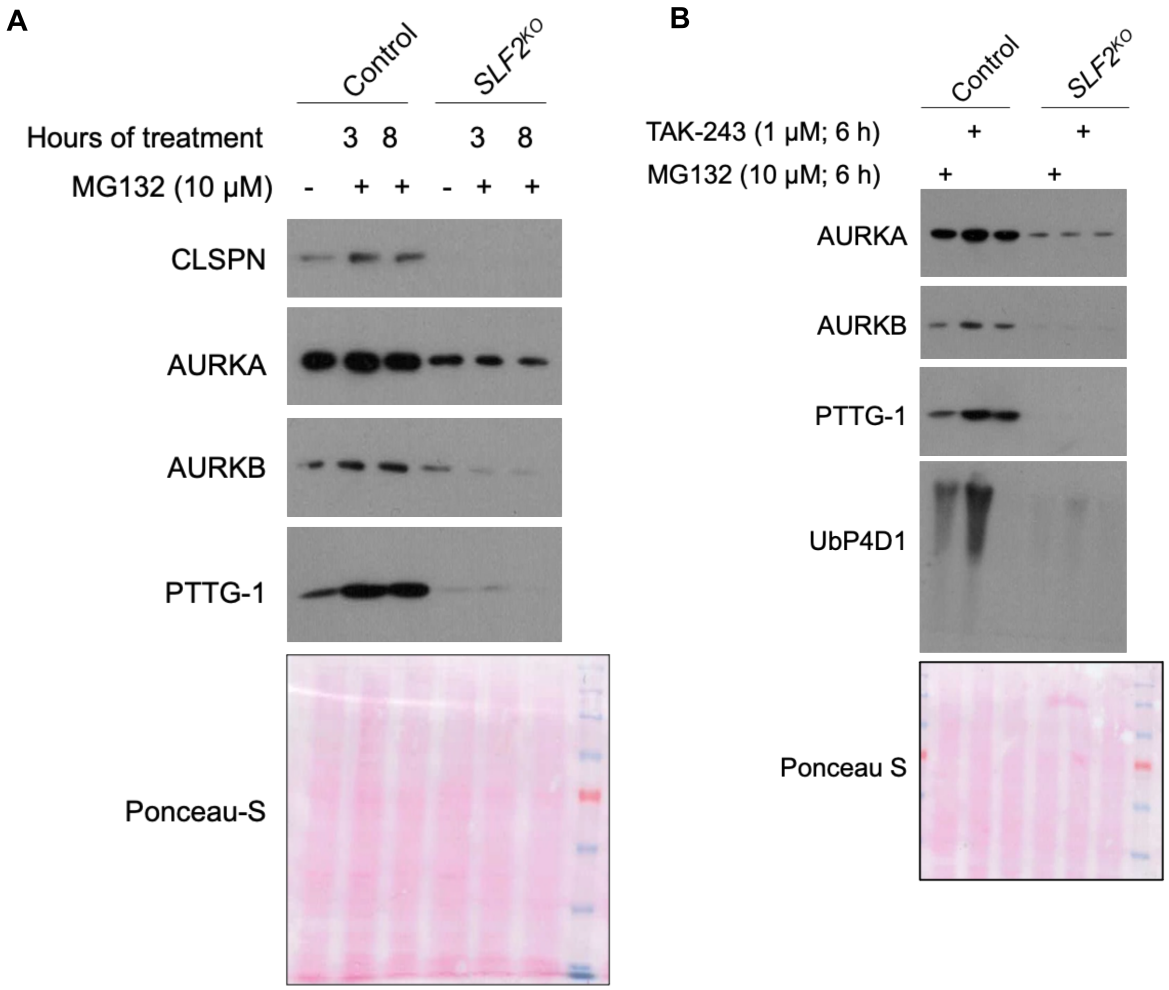

Appendix Figure S9. (A) Immunoblot analysis of indicated proteins in SU-DHL-5 control and *SLF2^KO^* cells lines treated with MG132 (10 µM) for the indicated time points. (B) Immunoblot analysis of indicated proteins in SU-DHL-5 control and SLF2KO cells lines treated with MG132 (10 µM) or TAK-234 (1 µM) for the indicated time points.

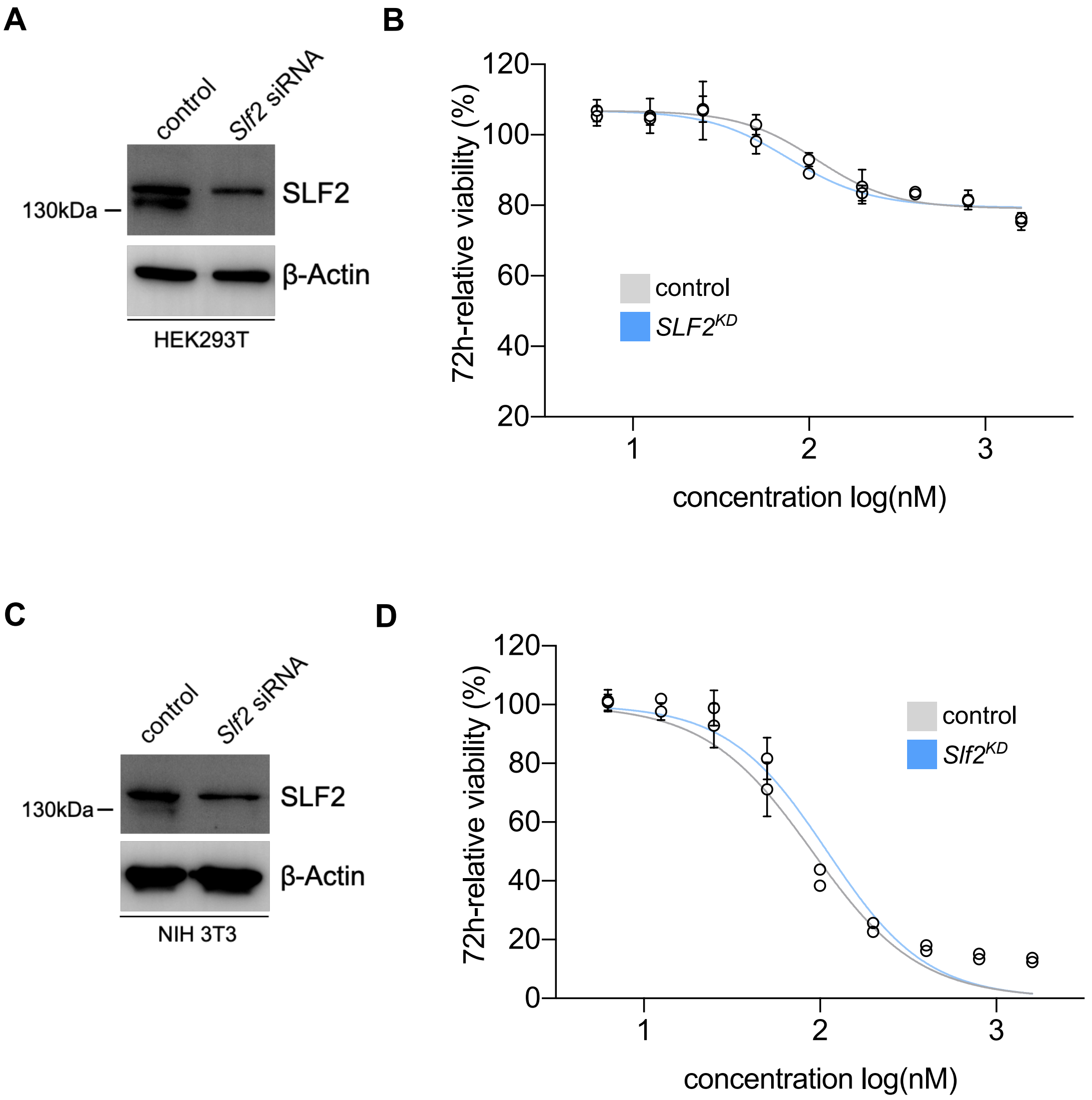

Appendix Figure S10. (A) Immunoblot analysis of indicated proteins in HEK293T cells transfected with either control siRNA or an siRNA targeting SLF2. (B) SUMO inhibitor (SUMOi) dose response curves of control and SLF2-depleted cell lines described in Figure S12A. Cells were treated with increasing concentrations of SUMOi for 72h and viability was determined by celltiterGlo measurement. Error bars represent SD from three independent experiments. (C) Immunoblot analysis of indicated proteins in NIH3T3 cells transfected with either control siRNA or an siRNA targeting Slf2. (D) SUMO inhibitor (SUMOi) dose response curves of control and SLF2-depleted cell lines described in Figure S12C. Cells were treated with increasing concentrations of SUMOi for 72h and viability was determined by celltiterGlo measurement. Error bars represent SD from three independent experiments.

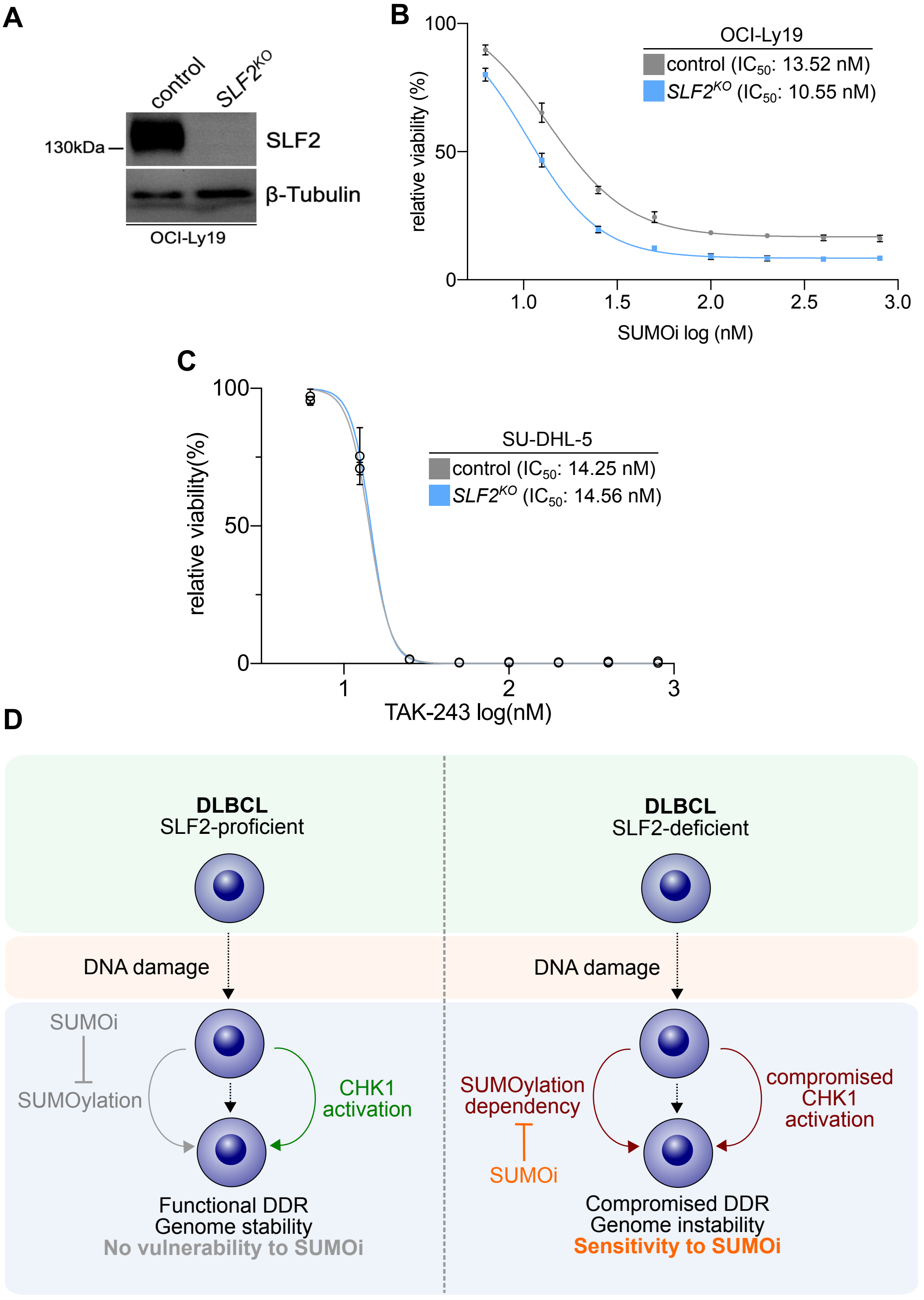

Appendix Figure S11. (A) Immunoblot analysis of indicated proteins in OCI-Ly19 control and *SLF2*^{KO} cells. (B) SUMO inhibitor (SUMOi (ML-093); 0, 6.25, 12.5, 25, 50, 100, 200, 400, 800 nM) dose response curves of control and SLF2-depleted cell lines described in Figure S11A. Cells were treated with increasing concentrations of SUMOi for 72 hours and viability was determined by negative of DAPI staining and flow cytometry measurement. Error bars represent SD from three independent experiments. (C) TAK-243 (ubiquitin E1 inhibitor; 0, 6.25, 12.5, 25, 50, 100, 200, 400, 800 nM) dose response curves of control and SLF2-deficient SU-DHL-5 cell lines. Cells were treated with increasing concentrations of TAK-243 for 72 hours and viability was determined by negative of DAPI staining and flow cytometry measurement. Error bars represent SD from three independent experiments. (D) Model of the actionable molecular vulnerability to SUMOylation inhibition defined by SLF2 deficiency and associated impaired DNA damage response in DLBCL cells.

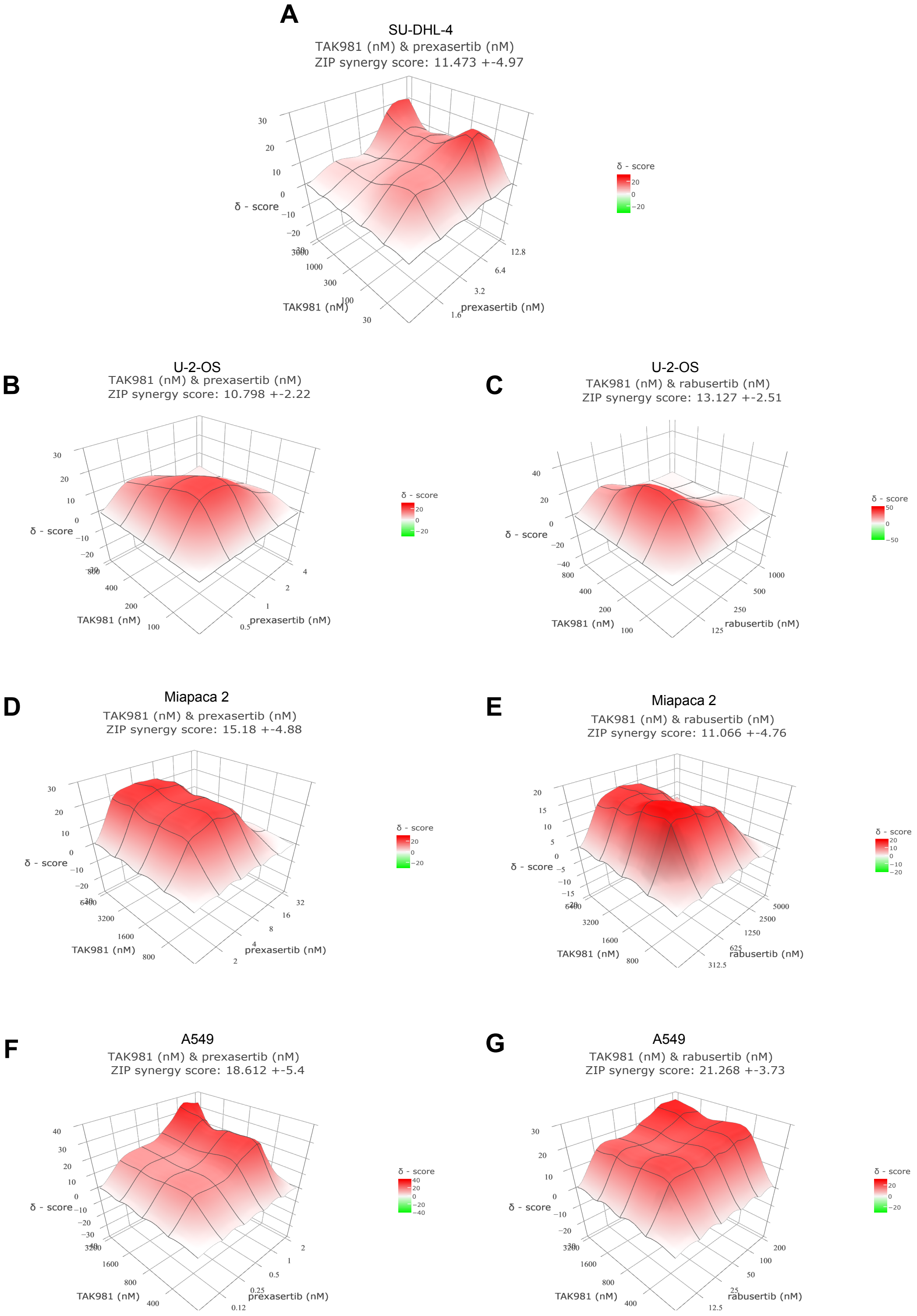

Appendix Figure S12. (A) ZIP synergy score plot of human DLBCL cell line OCI-Ly1 co-treated with increasing concentrations of SUMOi and Prexasertib. (B) ZIP synergy score plot of human osteosarcoma cell line U-2-OS co-treated with increasing concentrations of SUMOi and Prexasertib. (C) ZIP synergy score plot of human osteosarcoma cell line U-2-OS co-treated with increasing concentrations of SUMOi and Rabusertib. (D) ZIP synergy score plot of human pancreatic ductal adenocarcinoma cell line Miapaca2 co-treated with increasing concentrations of SUMOi and Prexasertib. (E) ZIP synergy score plot of human pancreatic ductal adenocarcinoma cell line Miapaca2 co-treated with increasing concentrations of SUMOi and Rabusertib. (F) ZIP synergy score plot of human non-small cell lung cancer cell line A549 co-treated with increasing concentrations of SUMOi and Prexasertib. (G) ZIP synergy score plot of human non-small cell lung cancer cell line A549 co-treated with increasing concentrations of SUMOi and Rabusertib.

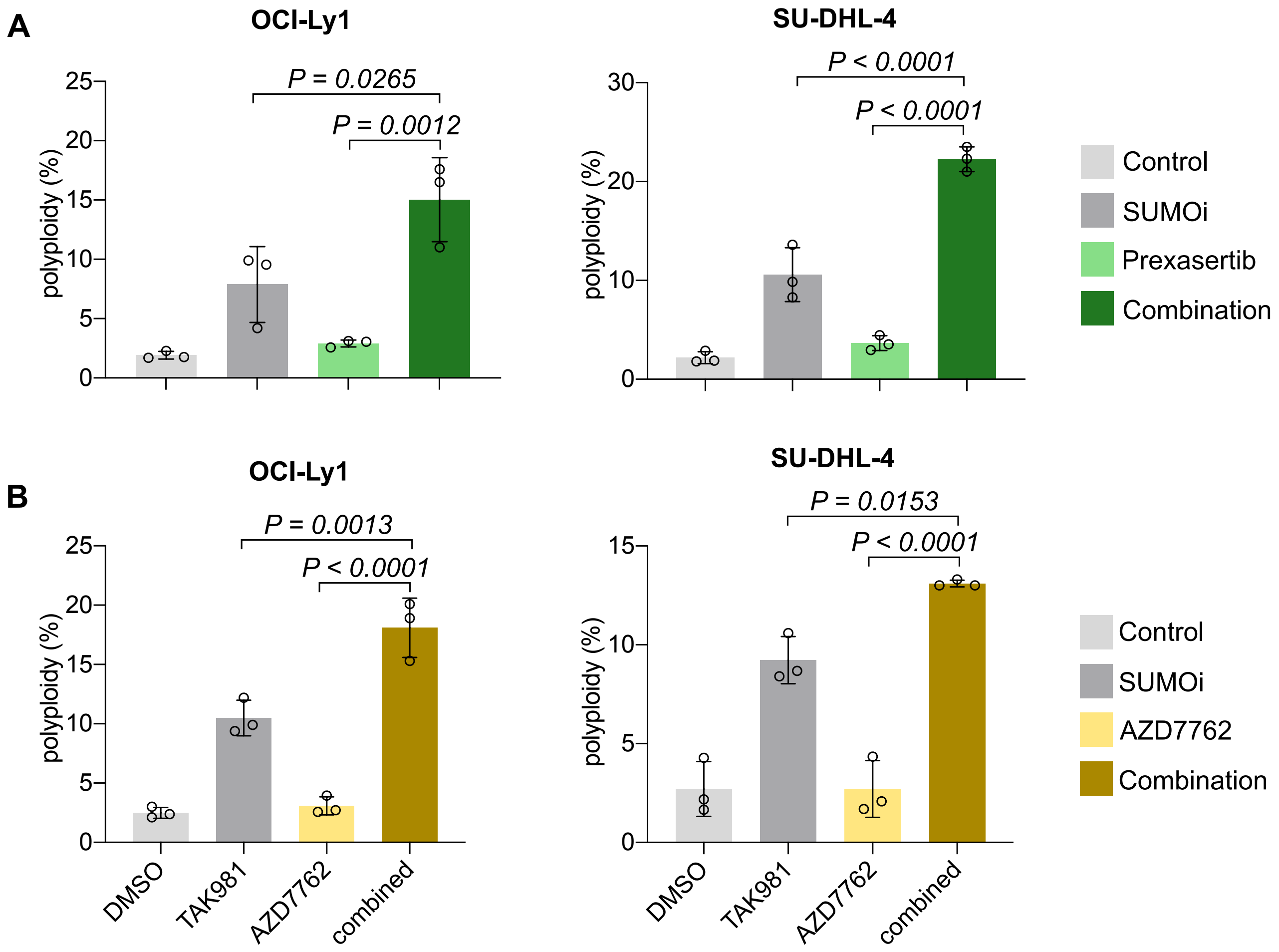

Appendix Figure S13. (A-B) Quantification of flow cytometry results for PI cell cycle analysis in human OCI-Ly1 and SU-DHL-4 cells after co-treatment with the indicated inhibitors (OCI-Ly1: SUMOi, 15 nM; prexasertib, 1nM; AZD7762, 110 nM, SU-DHL-4: SUMOi, 30 nM; prexasertib, 6.4nM; AZD7762, 110 nM) for 72h. P-value determined by two-way ANOVA; Tukey's multiple comparisons test. Error bars represent SD from three independent experiments.

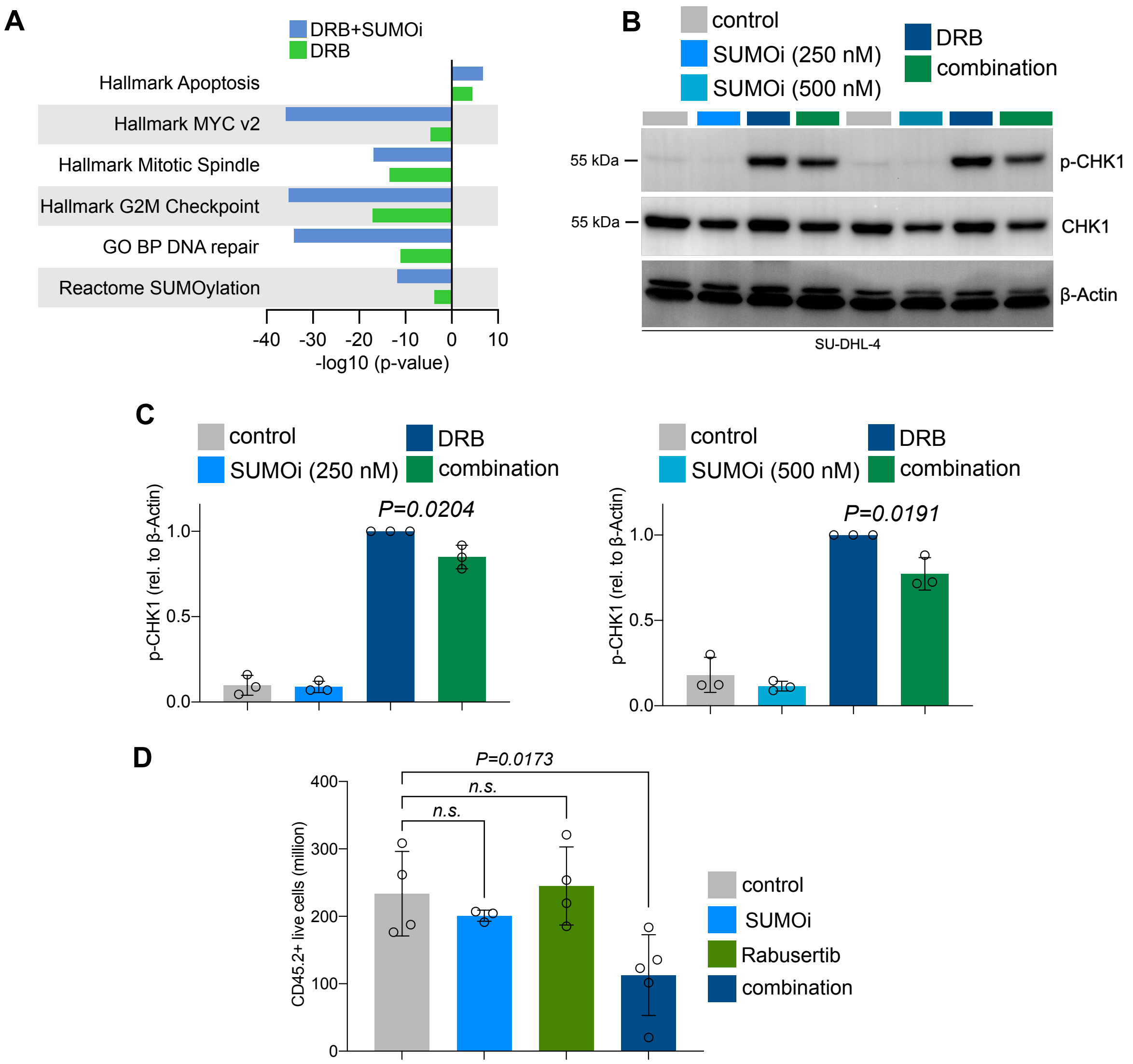

Appendix Figure S14. (A) Summary of GSEA of expression data derived from transcriptome profiling SU-DHL-4 cells treated with doxorubicin (0.5 µM, 3 hours) or doxorubicin (0.5 µM, 3 hours) in combination with SUMOi with the indicated gene sets. P-value determined by Kolmogorov-Smirnov test. (B) Immunoblot analysis of human SU-DHL-4 cell lines upon DRB treatment for indicated time points with the indicated antibodies. Cells have been pre-treated with indicated SUMOi concentrations for 24 hours. (C) Quantification of the p-CHK1 western blots from Figure S15B. Protein expression of p-CHK1 was normalized to β-Actin protein expression. p-CHK1 expression at 3 hours doxorubicin treatment was arbitrarily set to 1. Data are presented as mean +/- SD of n=3 independent experiments. *P-value* determined by one-way ANOVA; Tukey's multiple comparisons test. (D) Primary murine *Eµ-Myc* lymphoma cells were injected into syngeneic CD45.1 recipient mice. Mice were treated as described with vehicle, SUMOi (TAK-981), Rabusertib or the combination of SUMOi and Rabusertib twice a week and acute treatment effects were analyzed by flow cytometry.
