# Supplementary figures and images for: Actionable loss of SLF2 drives B‐cell lymphomagenesis and impairs the DNA damage response
Source: EMBO Mol Med. 2023 Jul 24;15(9):e16431. doi: 10.15252/emmm.202216431 (PMC10493575; doi:10.15252/emmm.202216431)

Appendix Figure S5F

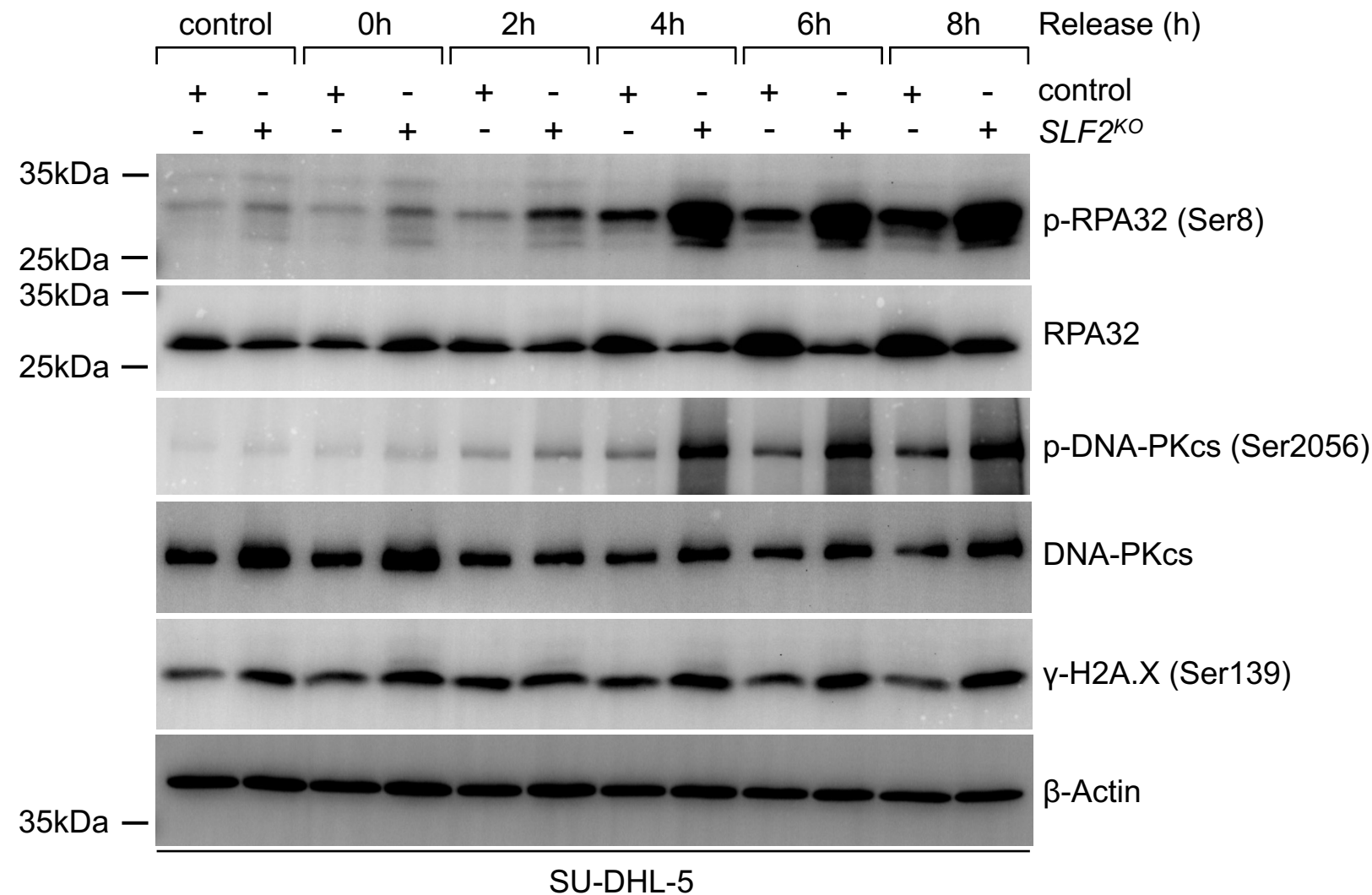

Appendix Figure S5F

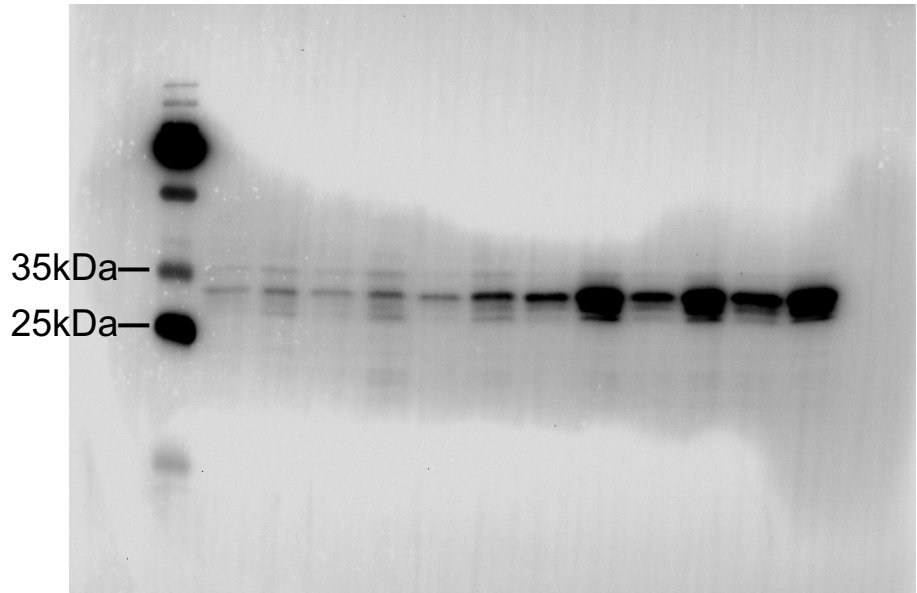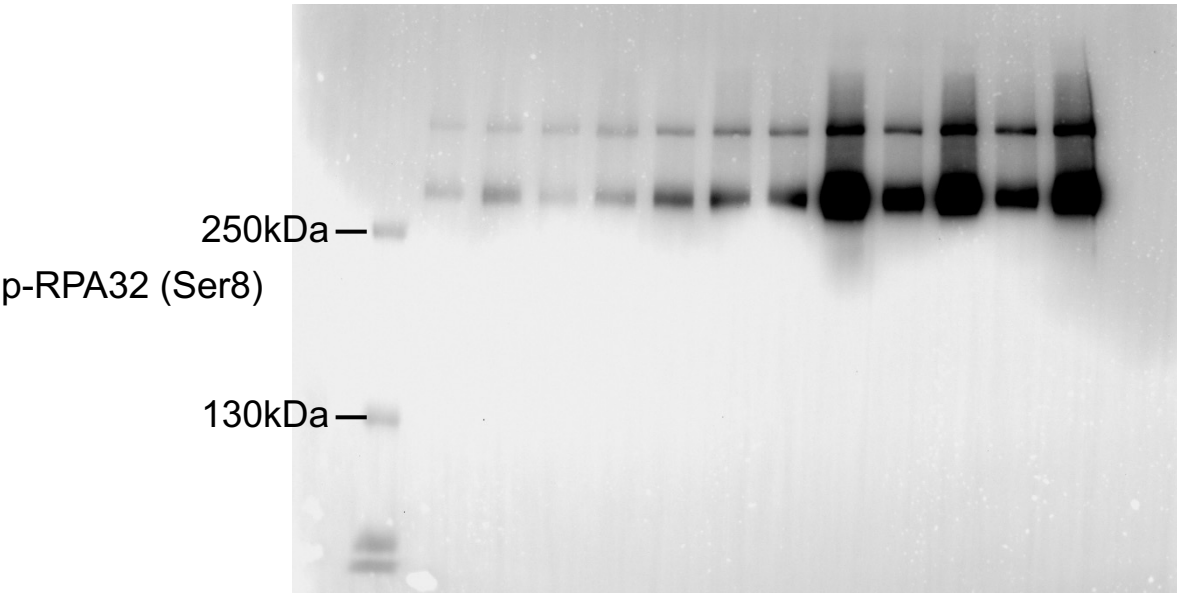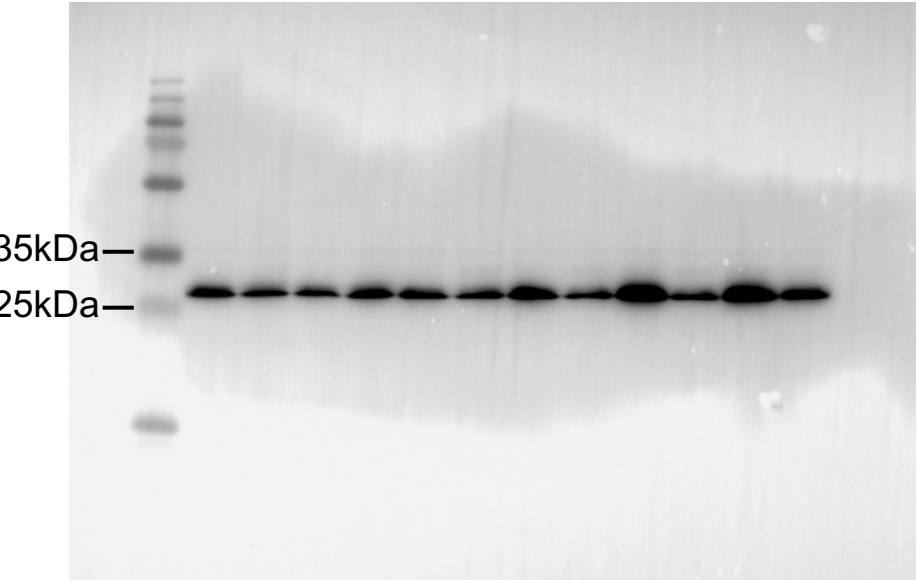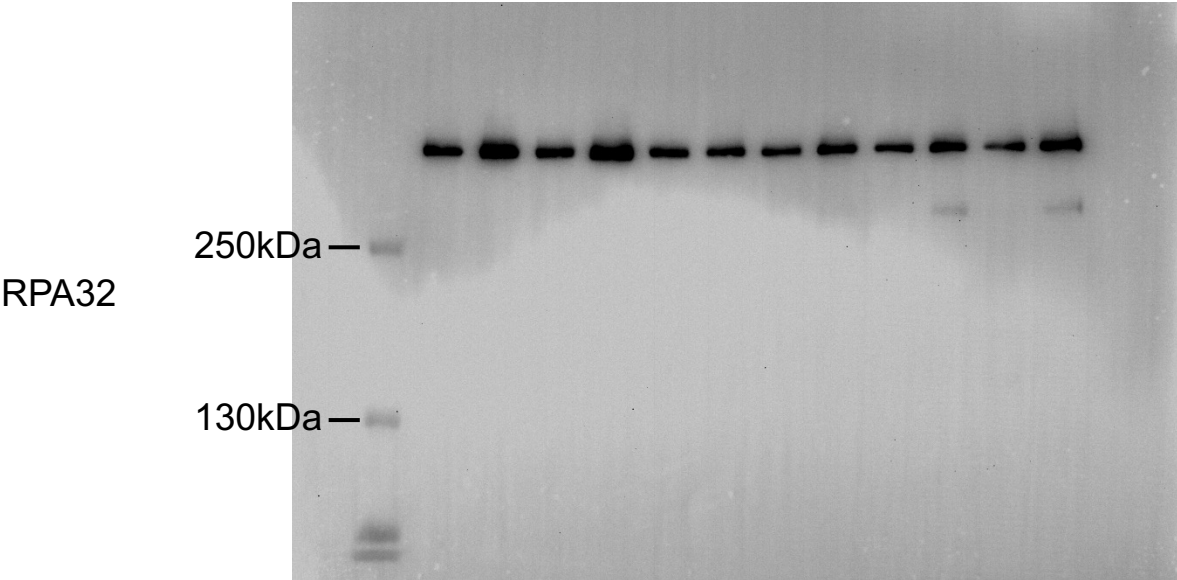

Appendix Figure S5F

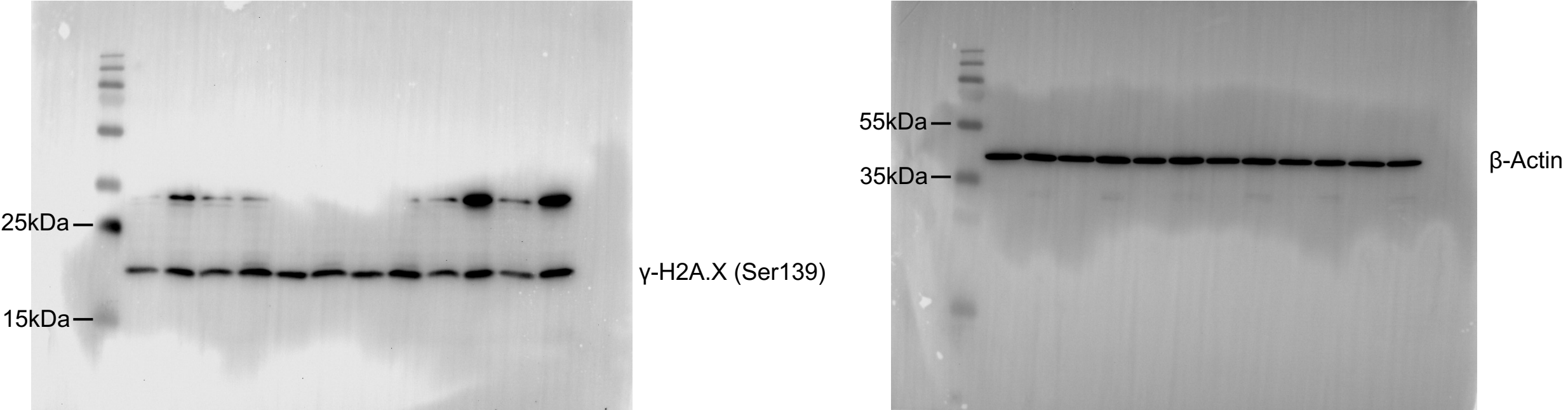

Supplement: Supplementary file 6 — Source Data for Appendix [file EMMM-15-e16431-s008.zip › EMM-2022-16431-V3-Appendix_Figure_S5F-sd.pdf]

Appendix Figure S6A

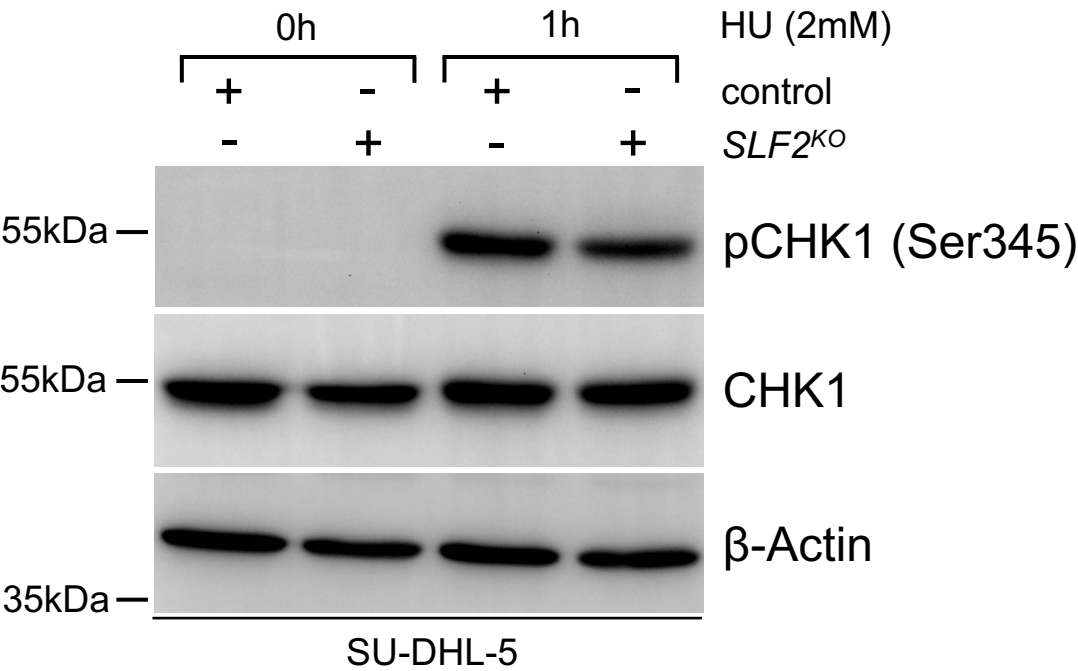

Appendix Figure S6A

p-CHK1 (Ser345)

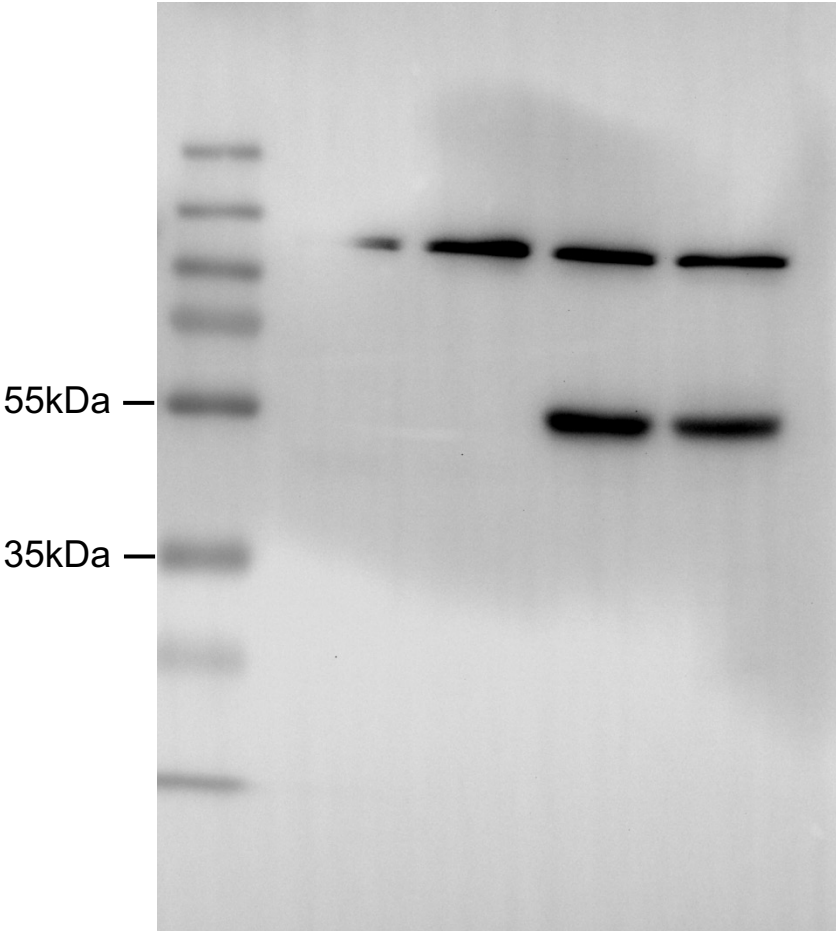

CHK1

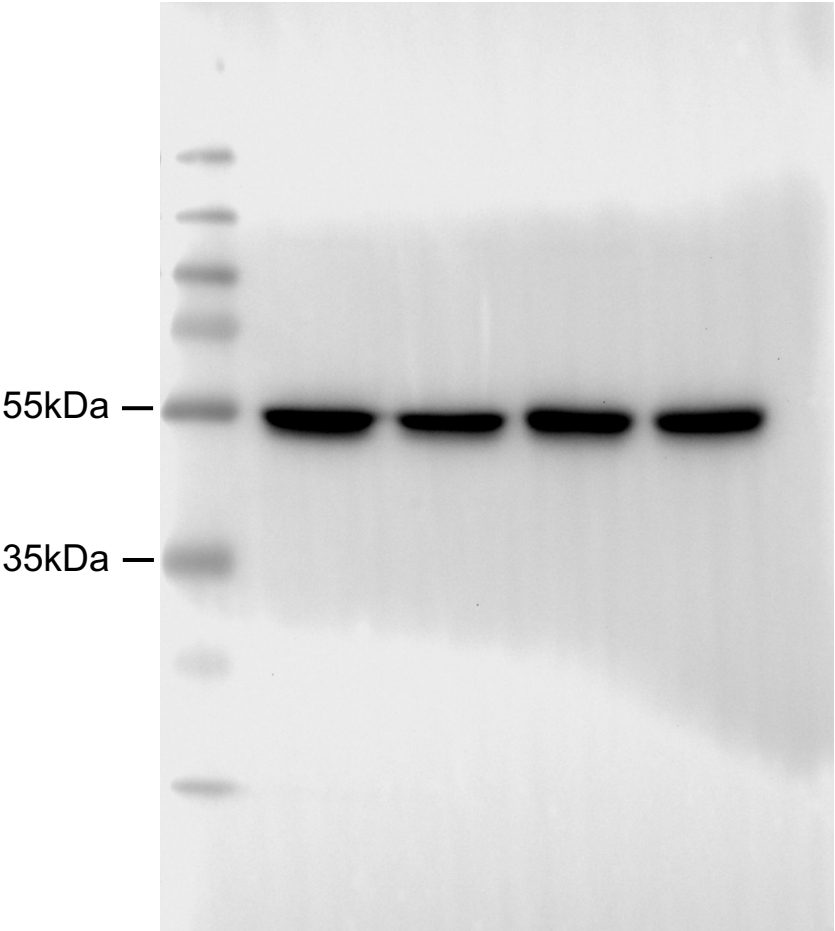

$\beta$ -Actin

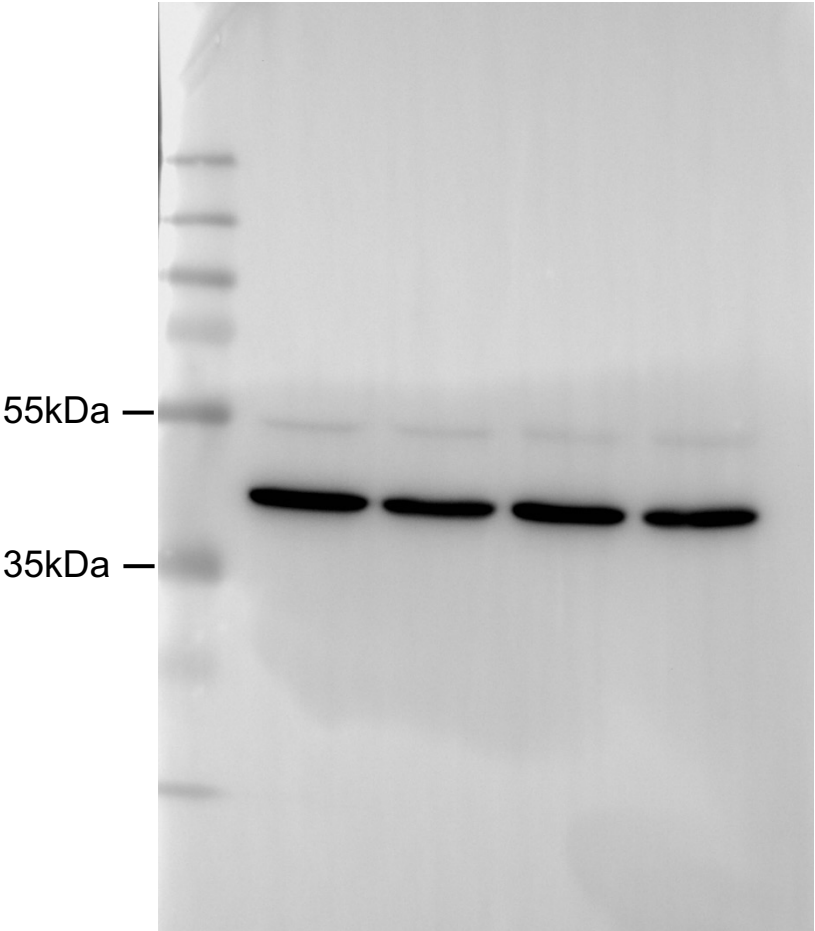

Supplement: Supplementary file 6 — Source Data for Appendix [file EMMM-15-e16431-s008.zip › EMM-2022-16431-V3-Appendix_Figure_S6A-sd.pdf]

Appendix Figure S6B

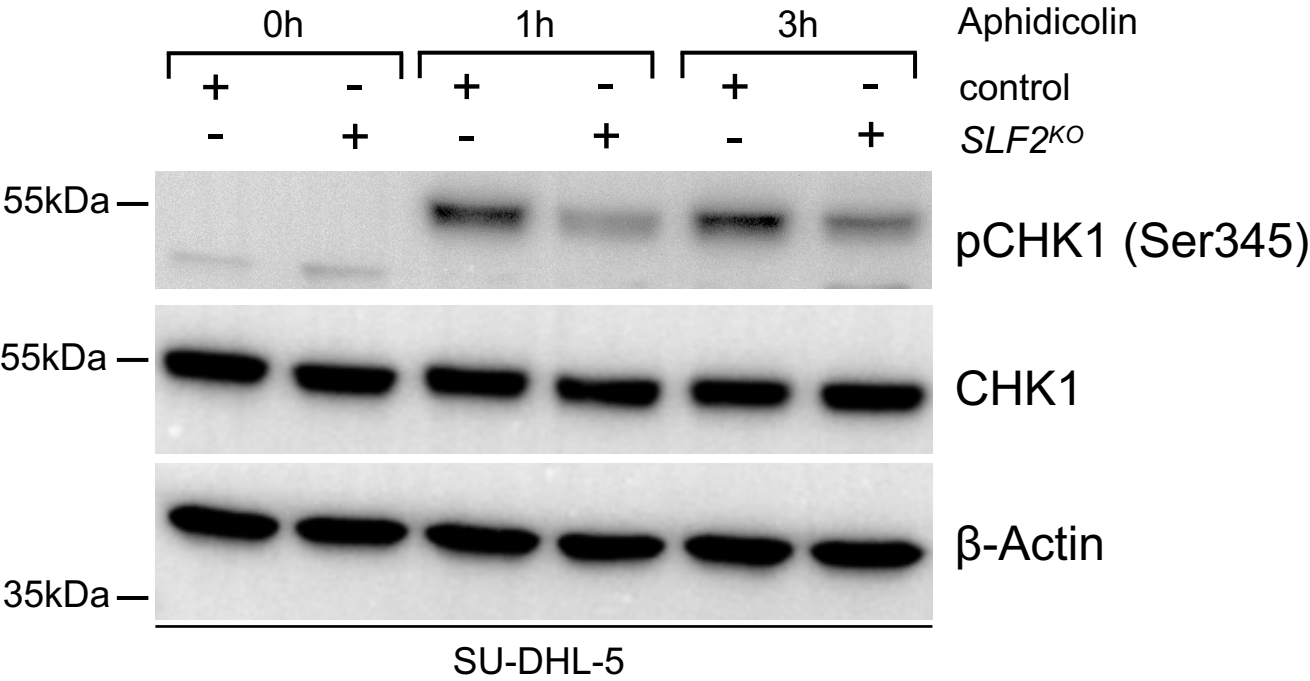

Appendix Figure S6B

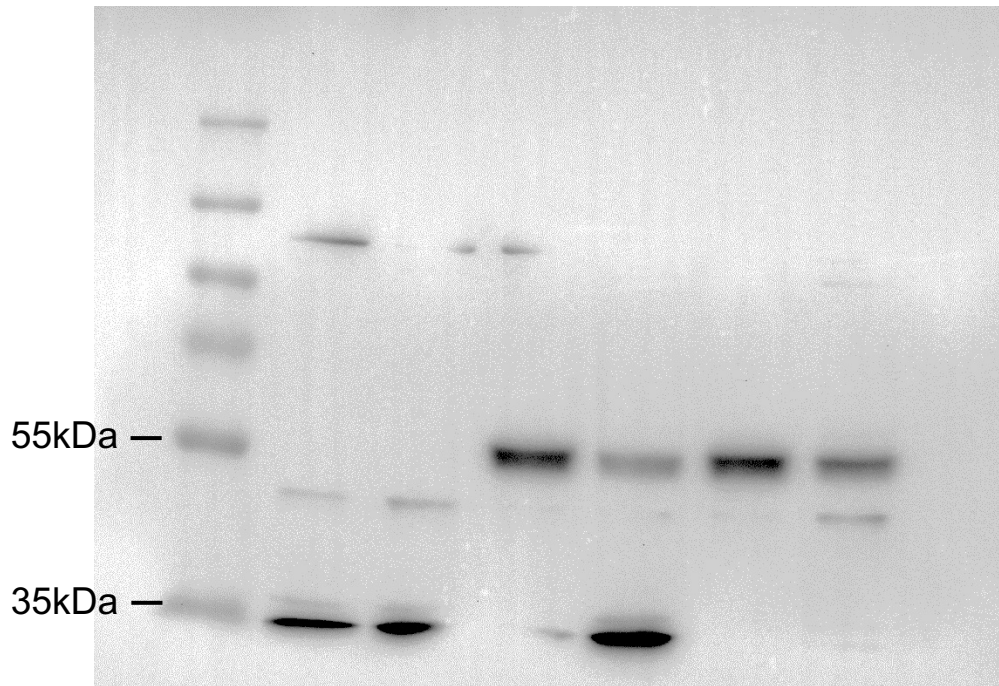

p-Chk1 (Ser345)

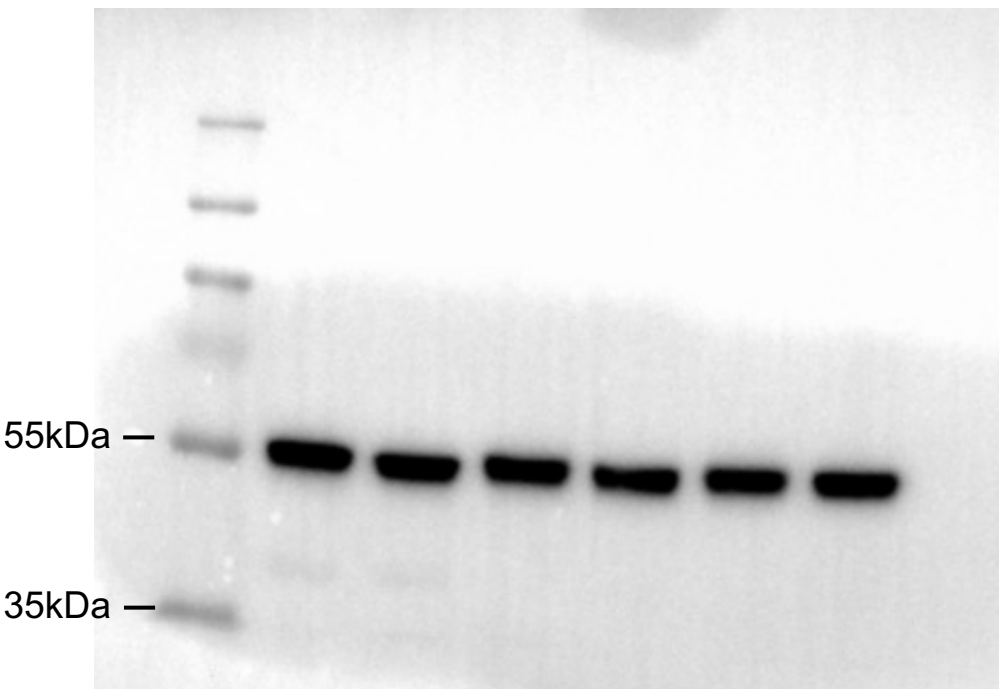

Chk1

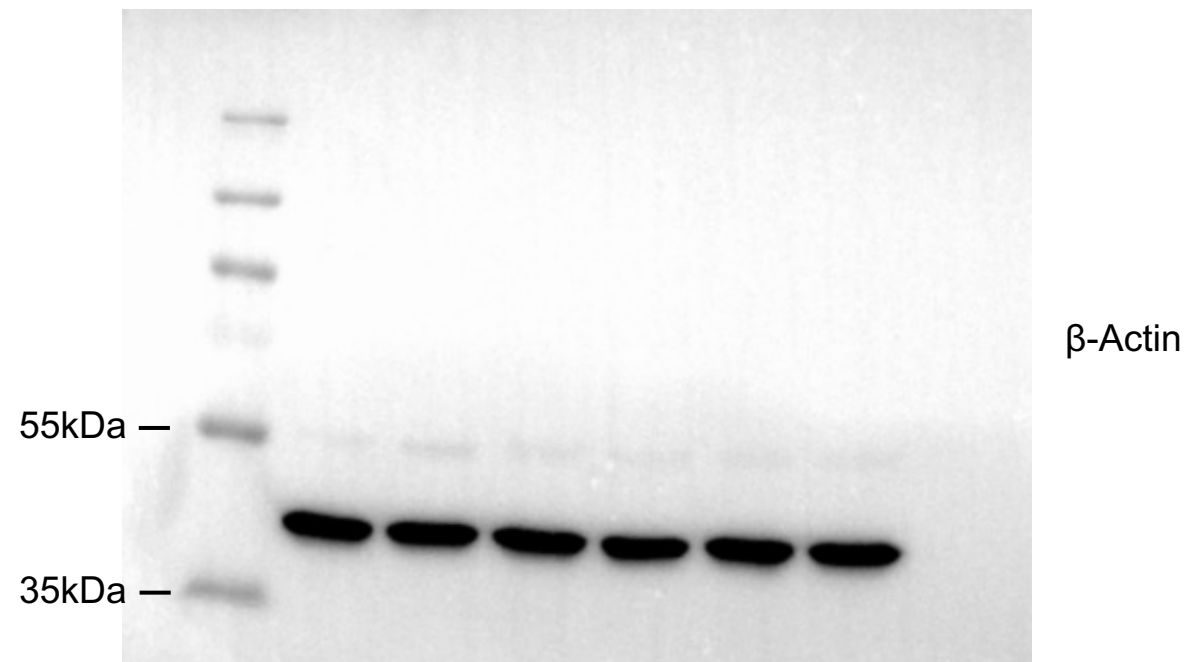

$\beta$ -Actin

Supplement: Supplementary file 6 — Source Data for Appendix [file EMMM-15-e16431-s008.zip › EMM-2022-16431-V3-Appendix_Figure_S6B-sd.pdf]

Appendix Figure S9A

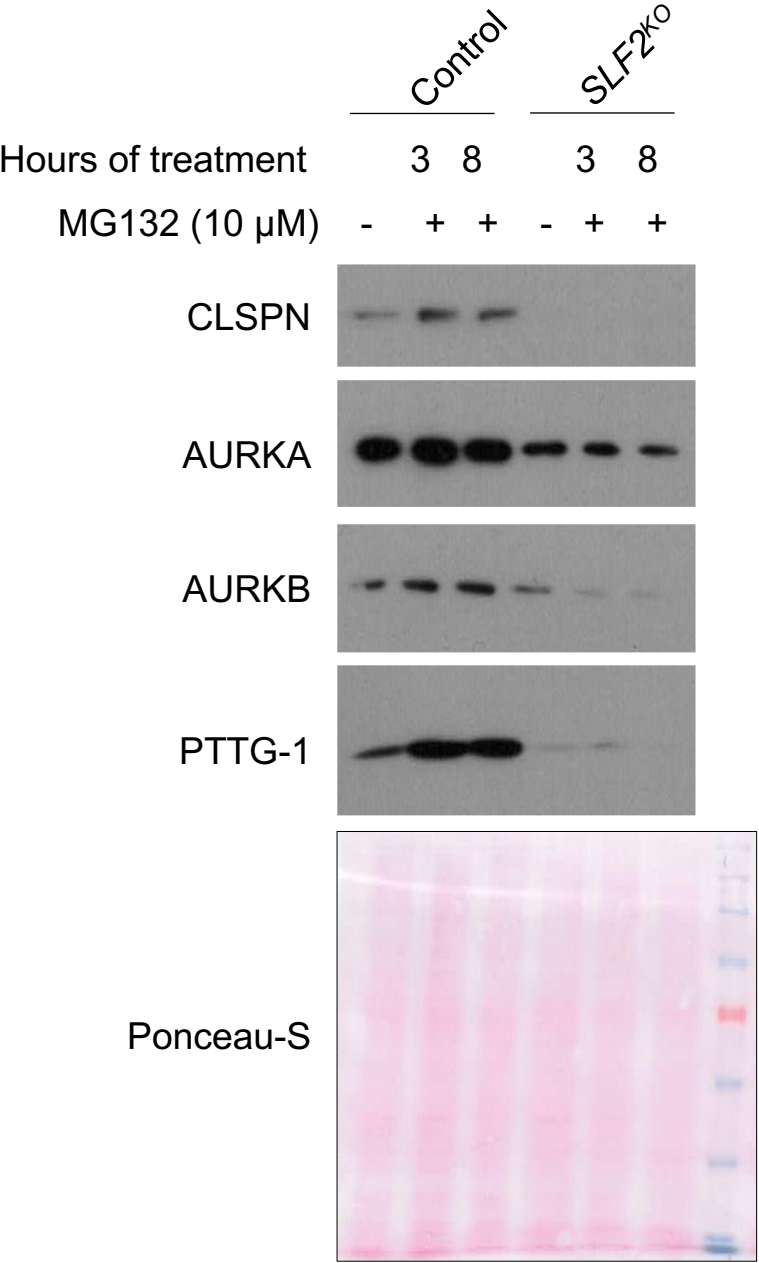

Appendix Figure S9A

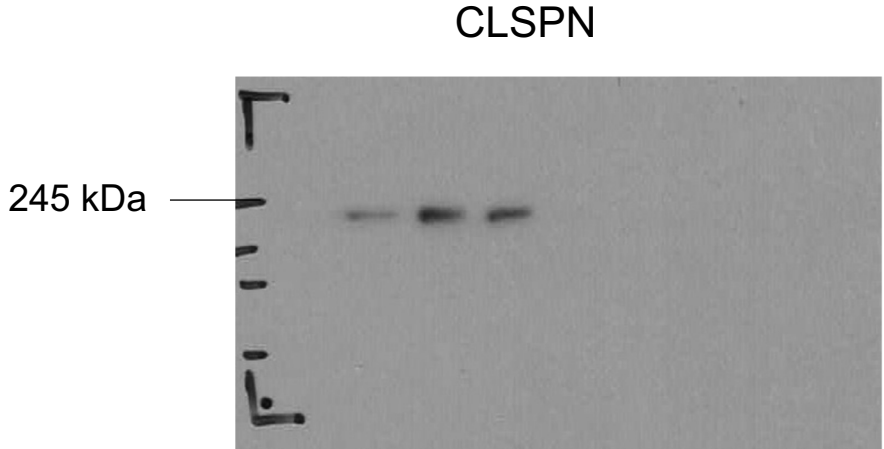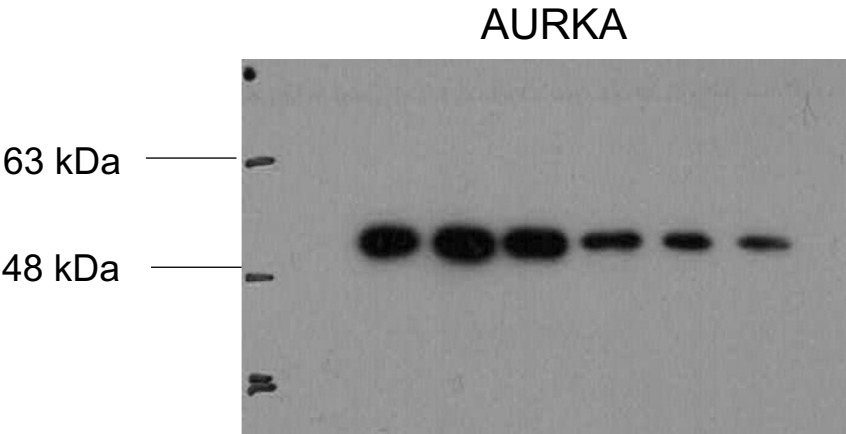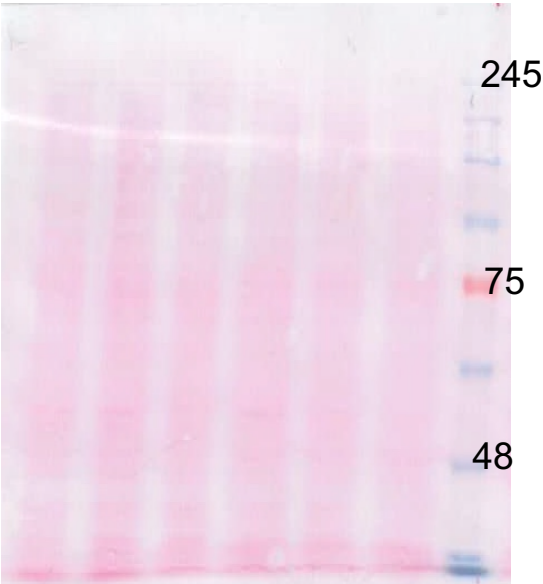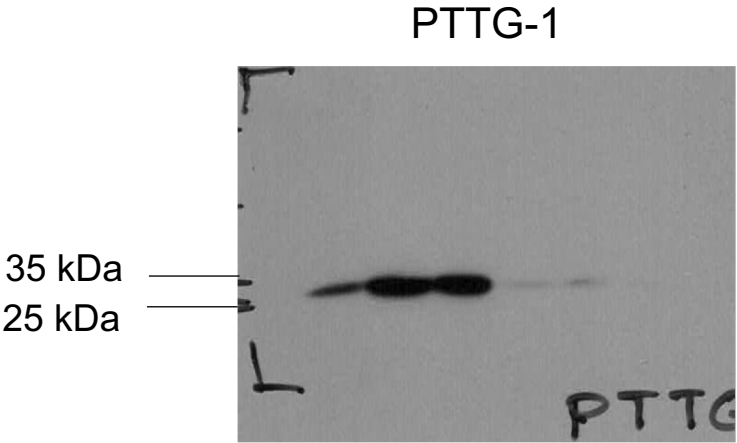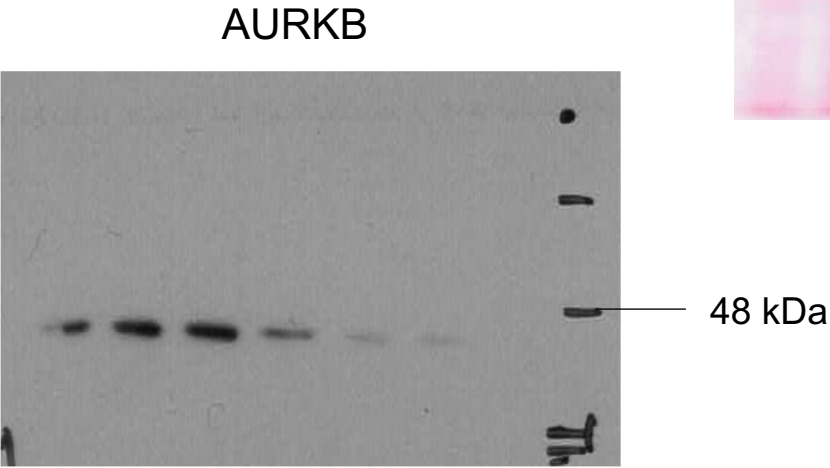

Supplement: Supplementary file 6 — Source Data for Appendix [file EMMM-15-e16431-s008.zip › EMM-2022-16431-V3-Appendix_Figure_S9A-sd.pdf]

Appendix Figure S9B

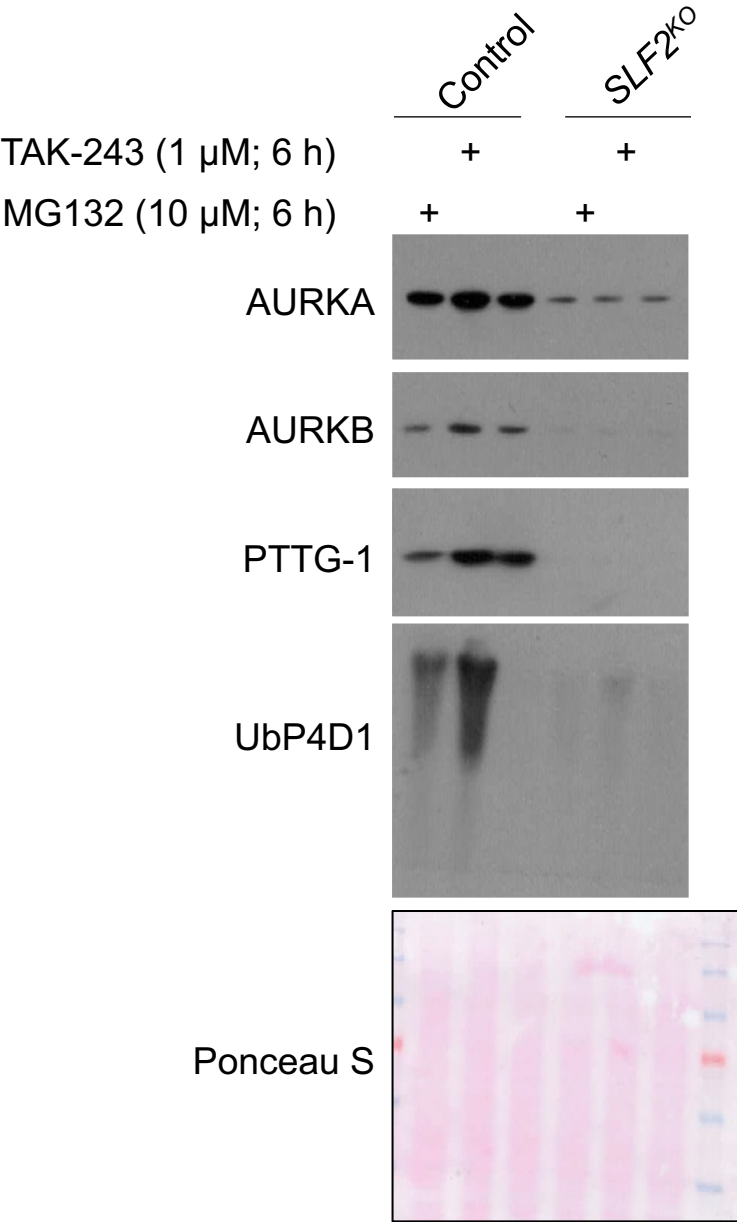

Appendix Figure S9B

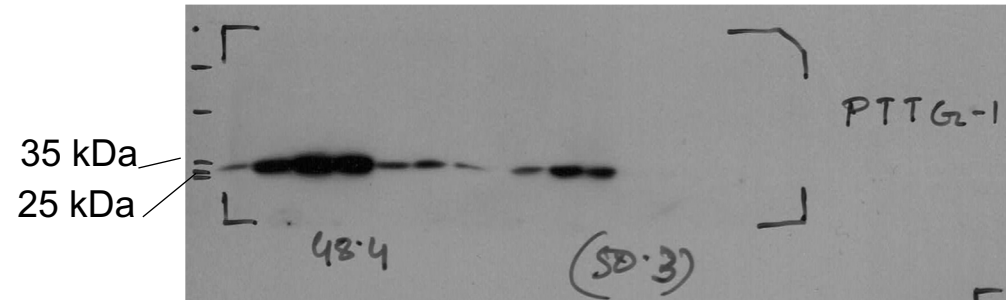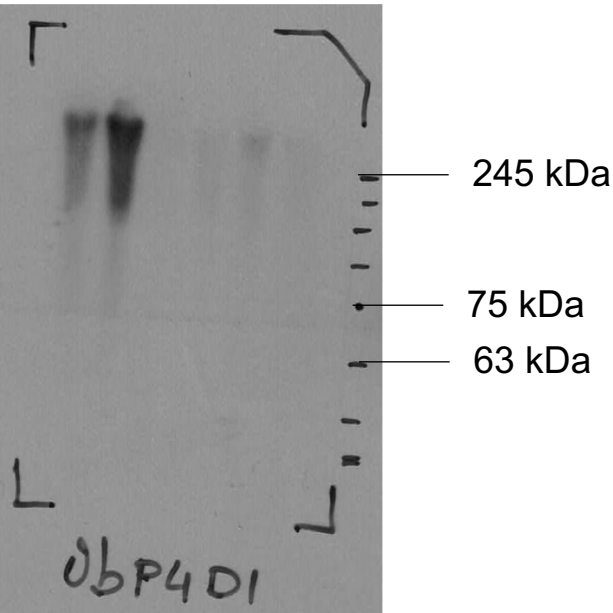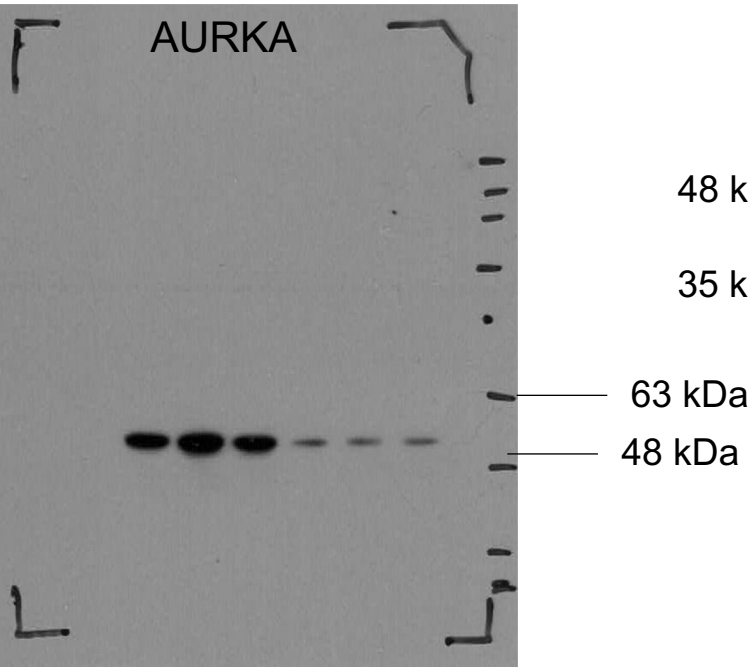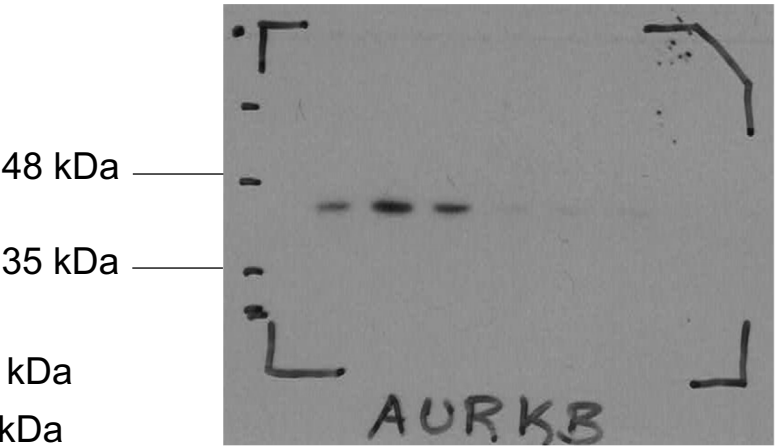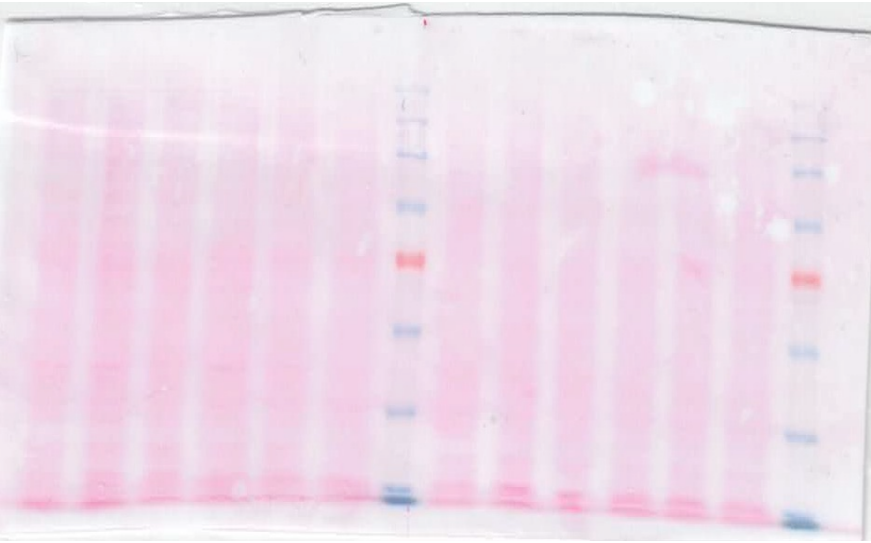

Supplement: Supplementary file 6 — Source Data for Appendix [file EMMM-15-e16431-s008.zip › EMM-2022-16431-V3-Appendix_Figure_S9B-sd.pdf]

Appendix Figure S10A

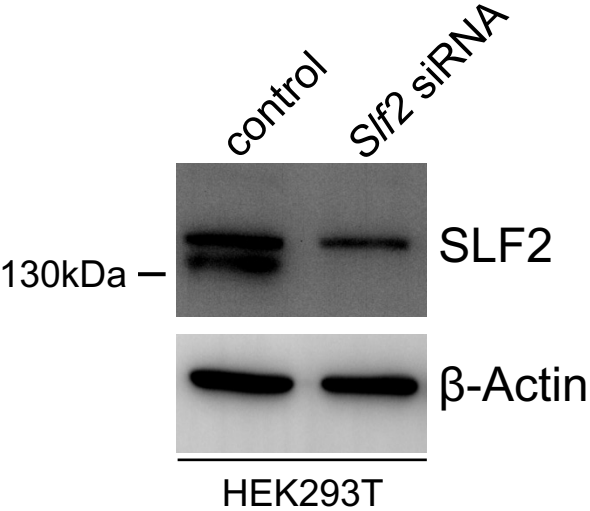

Appendix Figure S10C

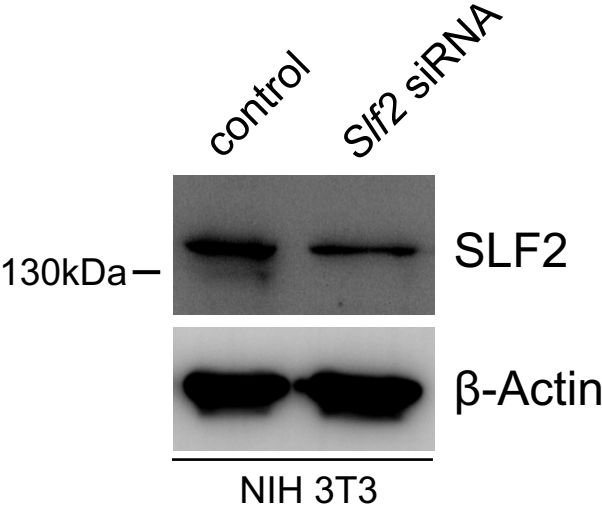

Appendix Figure S10A and S10C

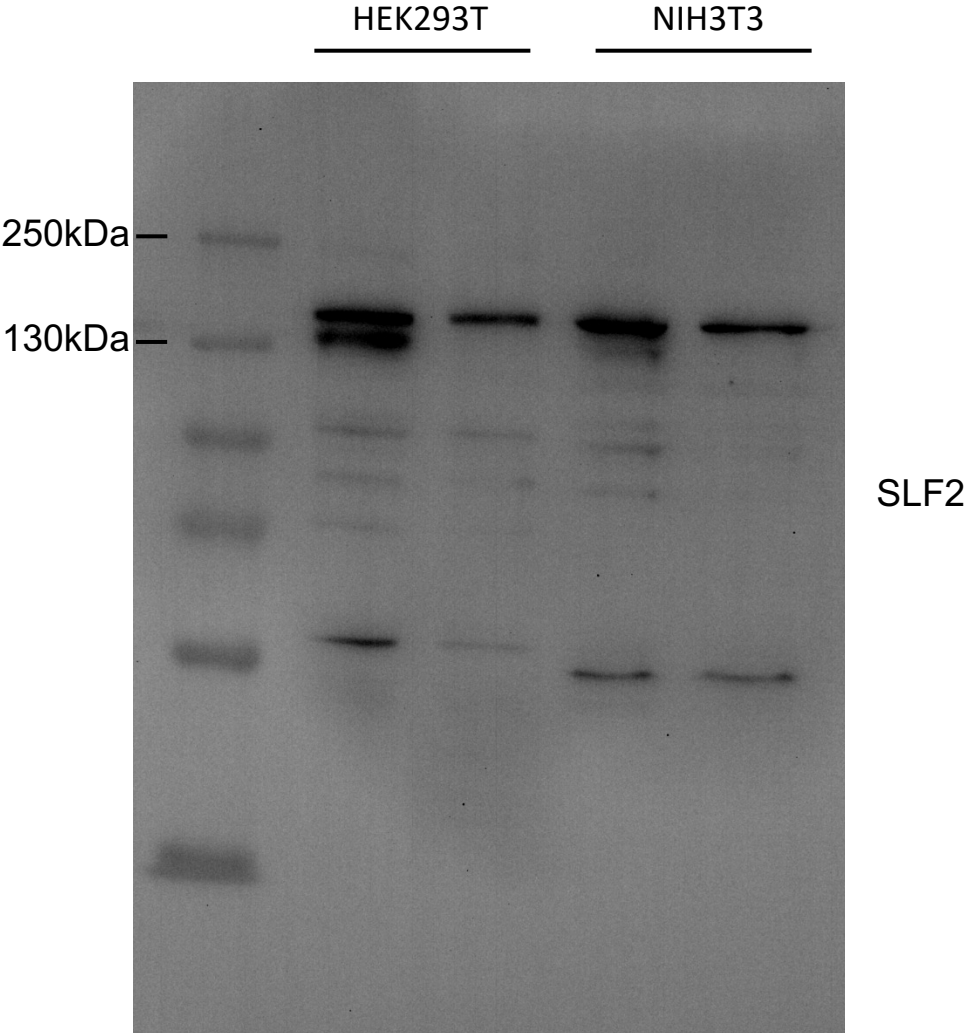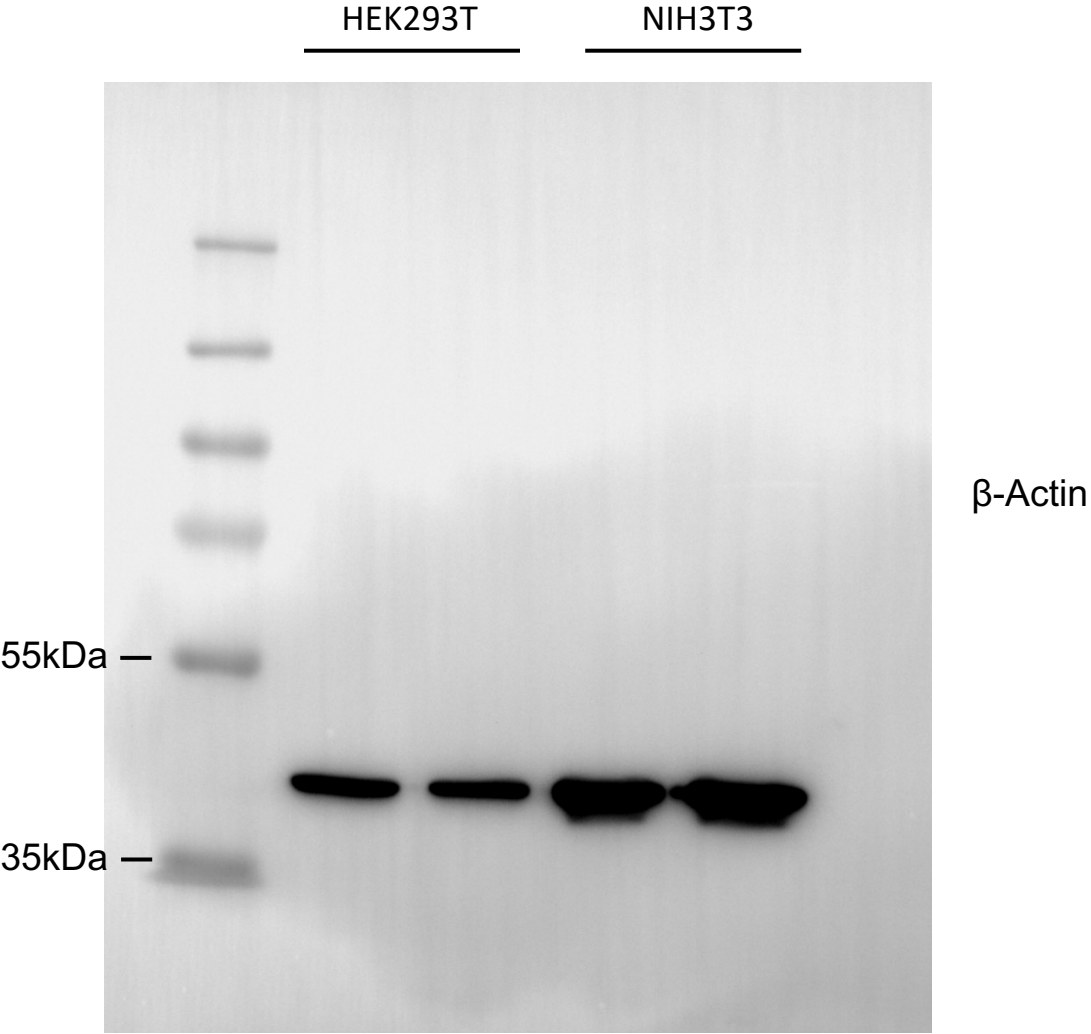

Supplement: Supplementary file 6 — Source Data for Appendix [file EMMM-15-e16431-s008.zip › EMM-2022-16431-V3-Appendix_Figure_S10A__B-sd.pdf]

Appendix Figure S11A

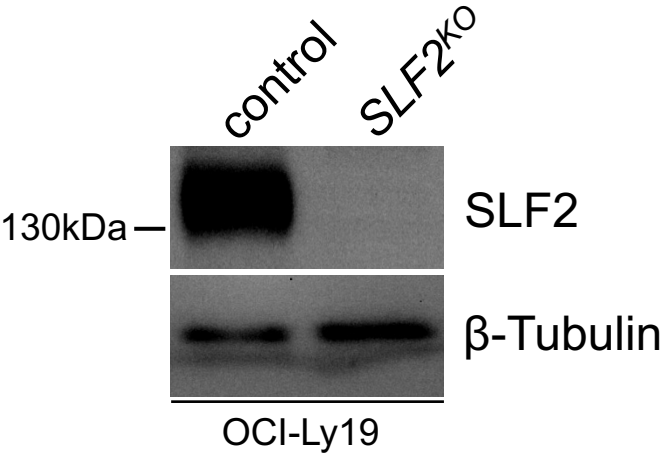

Appendix Figure S11A

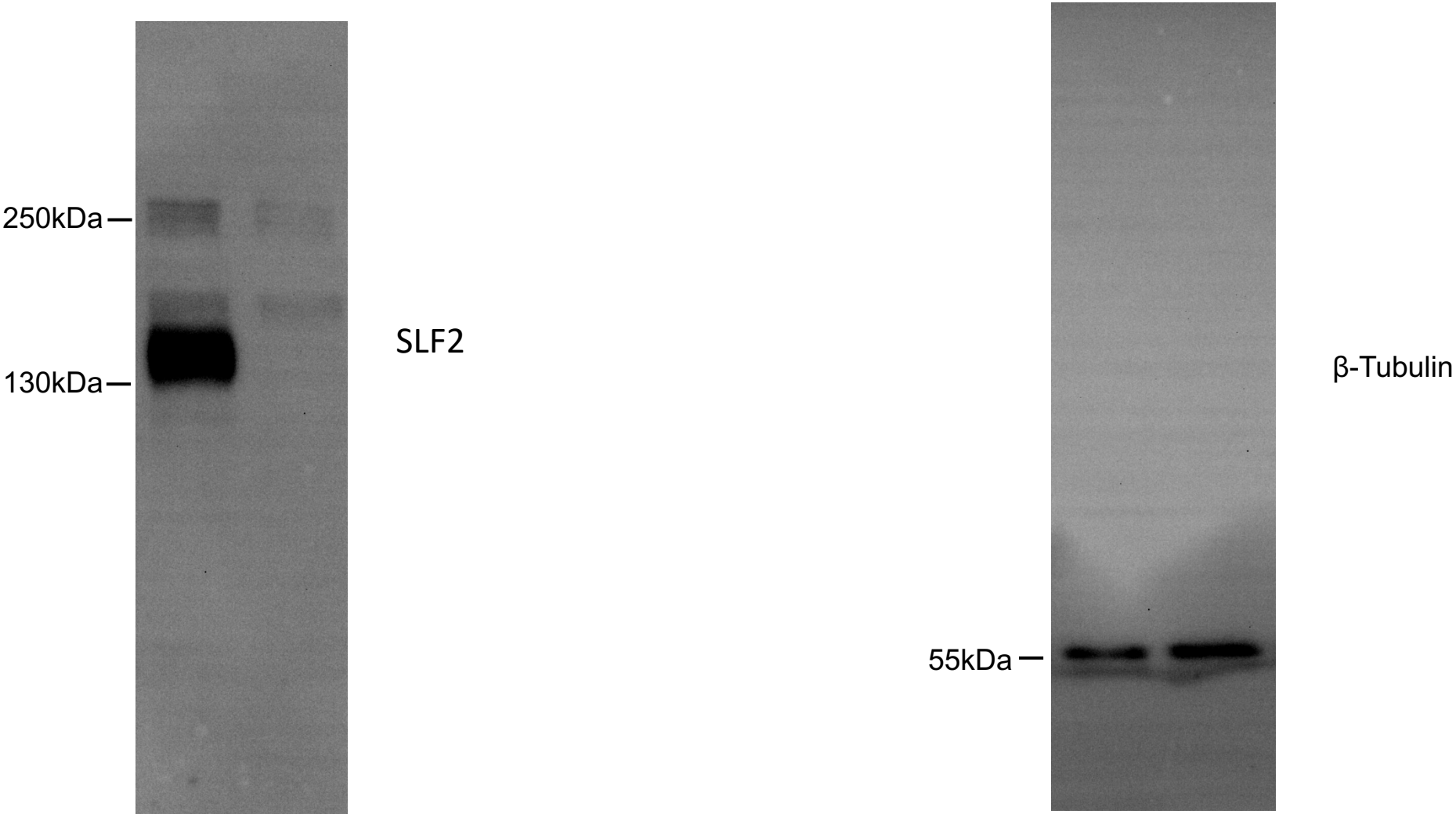

Supplement: Supplementary file 6 — Source Data for Appendix [file EMMM-15-e16431-s008.zip › EMM-2022-16431-V3-Appendix_Figure_S11A-sd.pdf]

Figure 1F

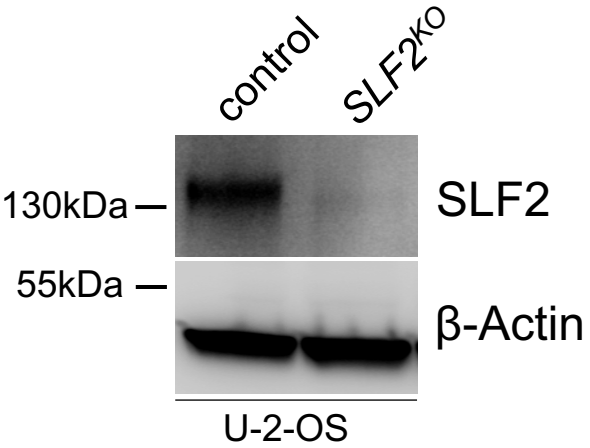

Figure 1F

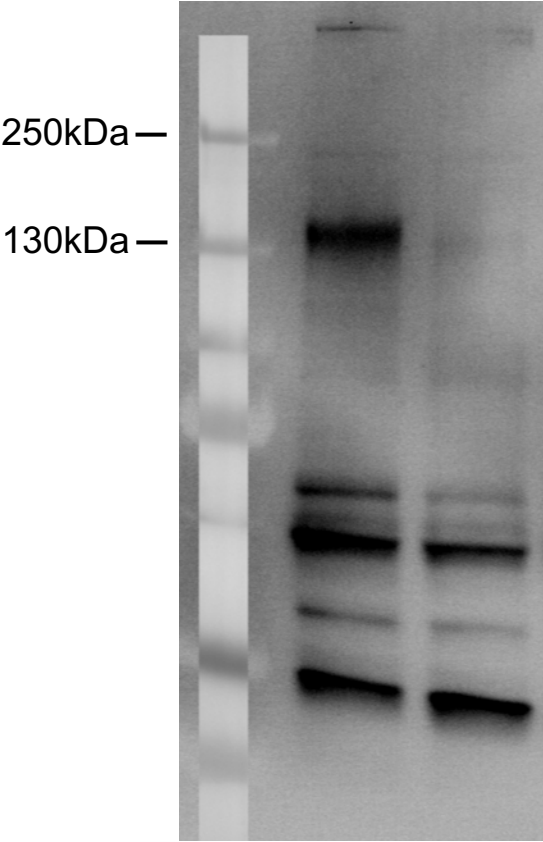

SLF2

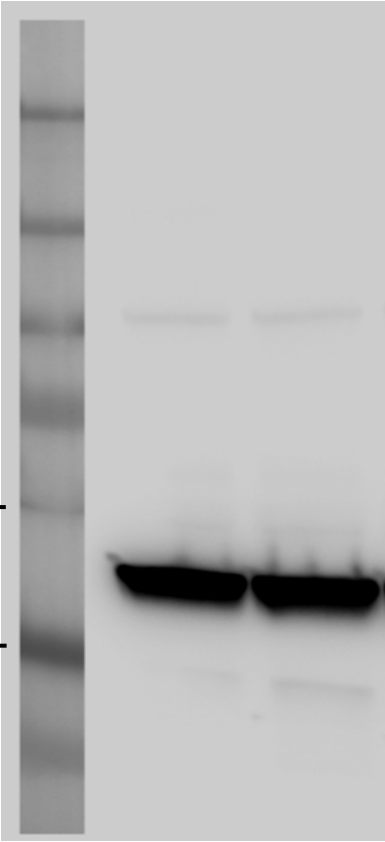

$\beta$ -Actin

Supplement: Supplementary file 7 — Source Data for Figure 1 [file EMMM-15-e16431-s005.pdf]

Figure 6C

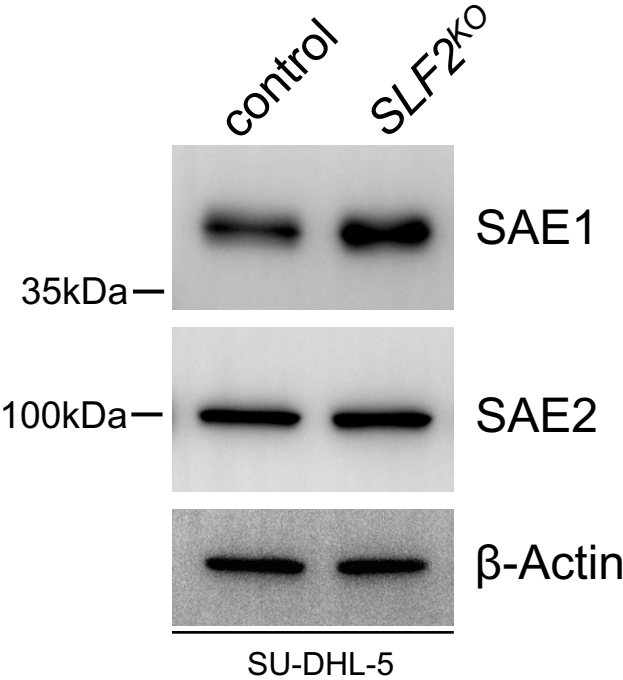

Figure 6C

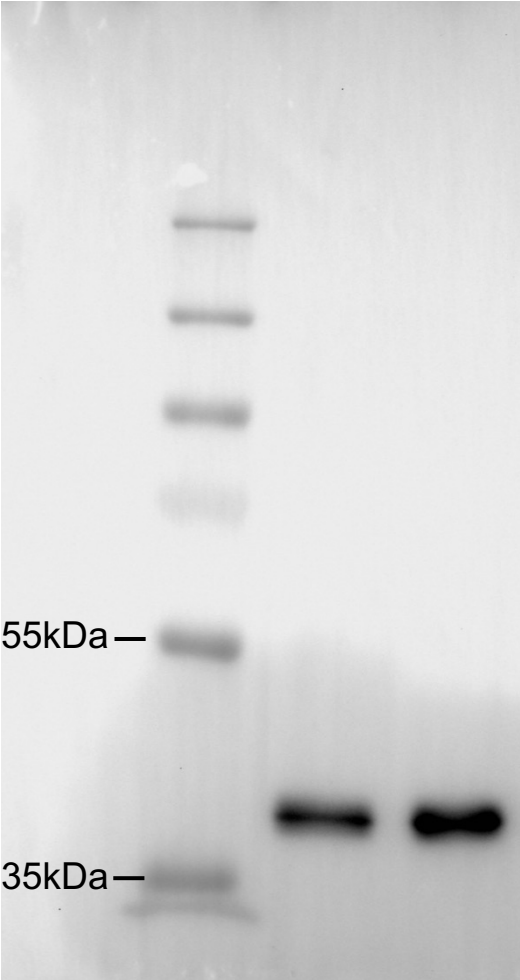

SAE1

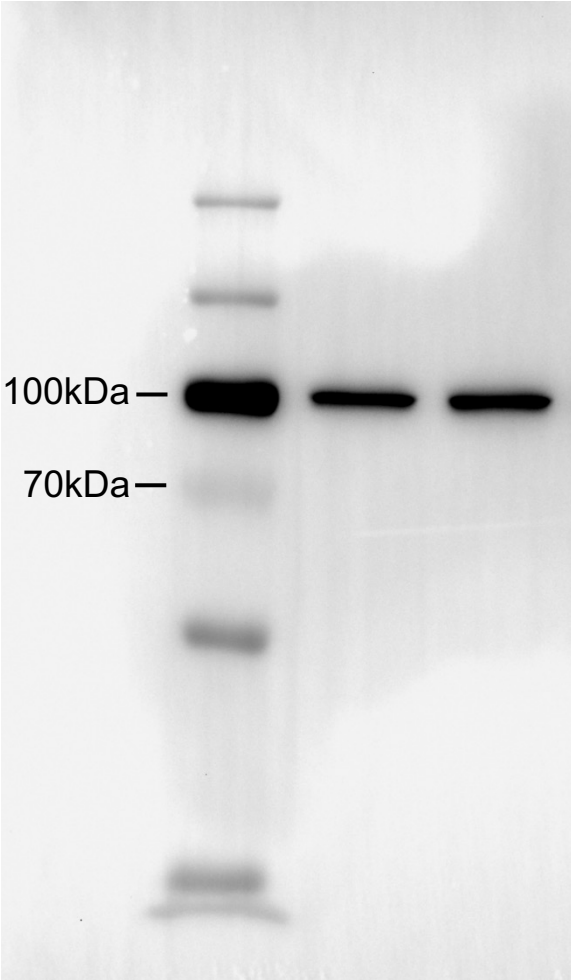

SAE2

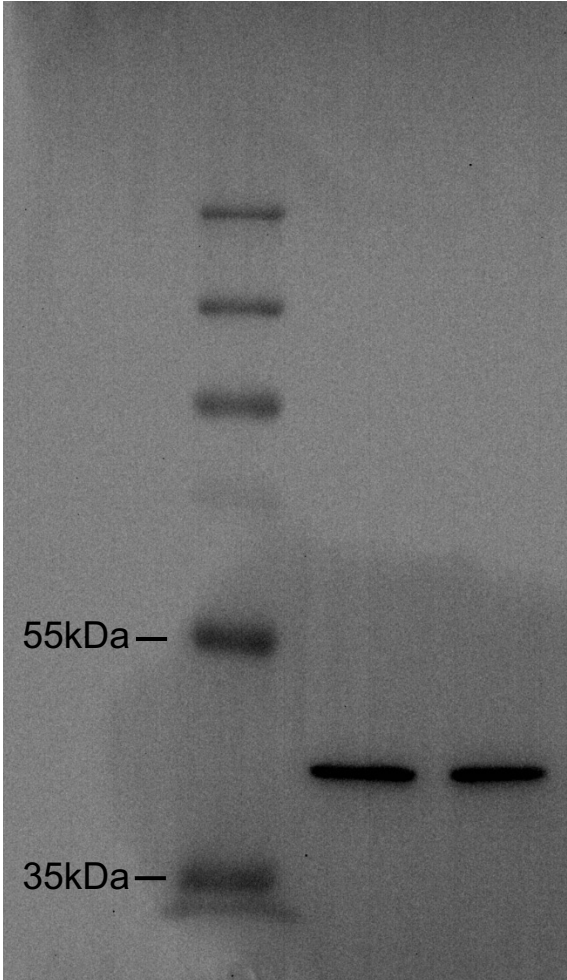

$\beta$ -Actin

Supplement: Supplementary file 10 — Source Data for Figure 6 [file EMMM-15-e16431-s001.pdf]

Figure 7A

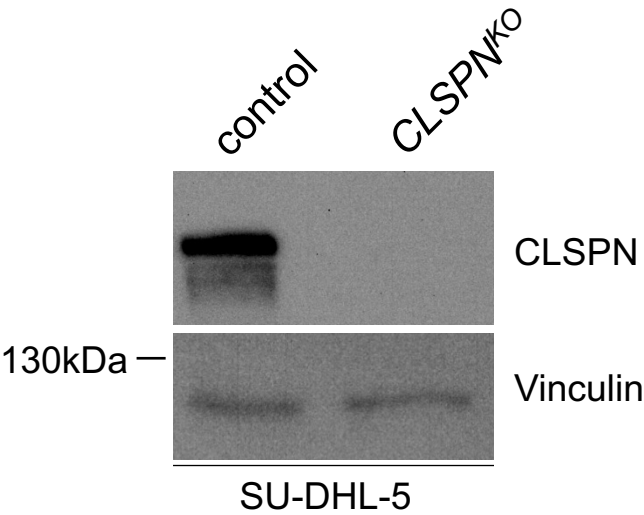

Figure 7A

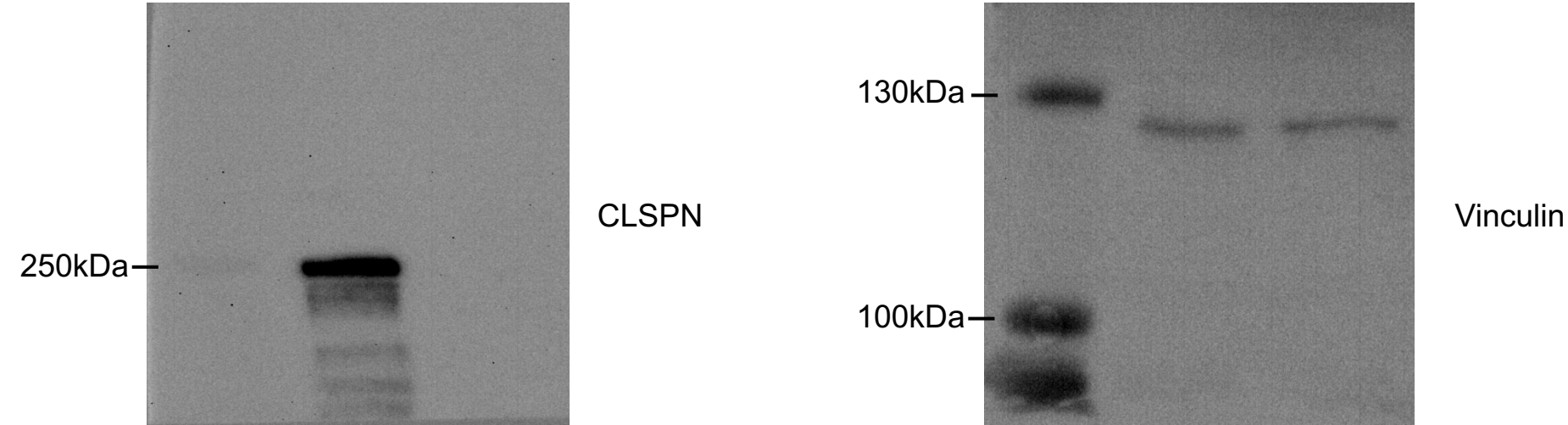

Figure 7B

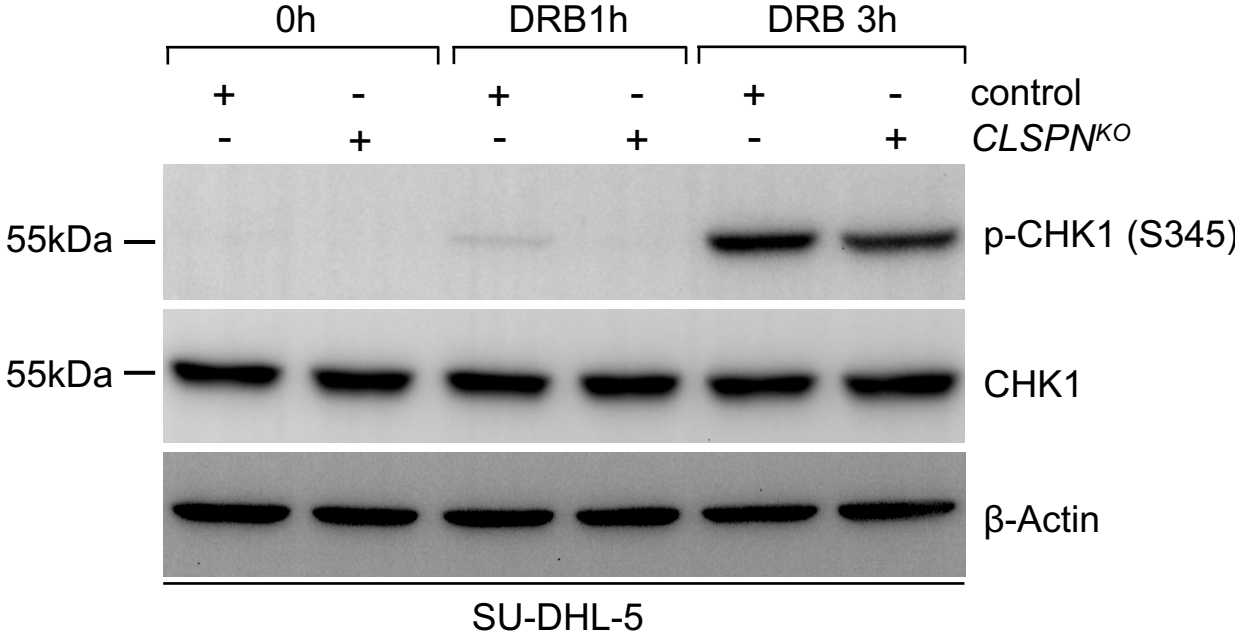

Figure 7B

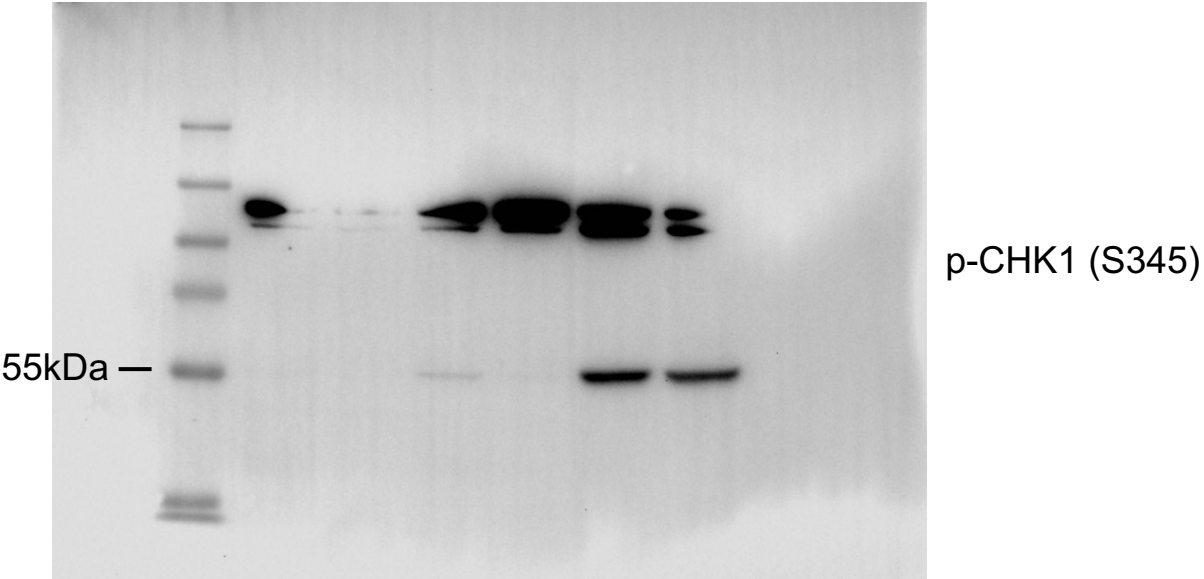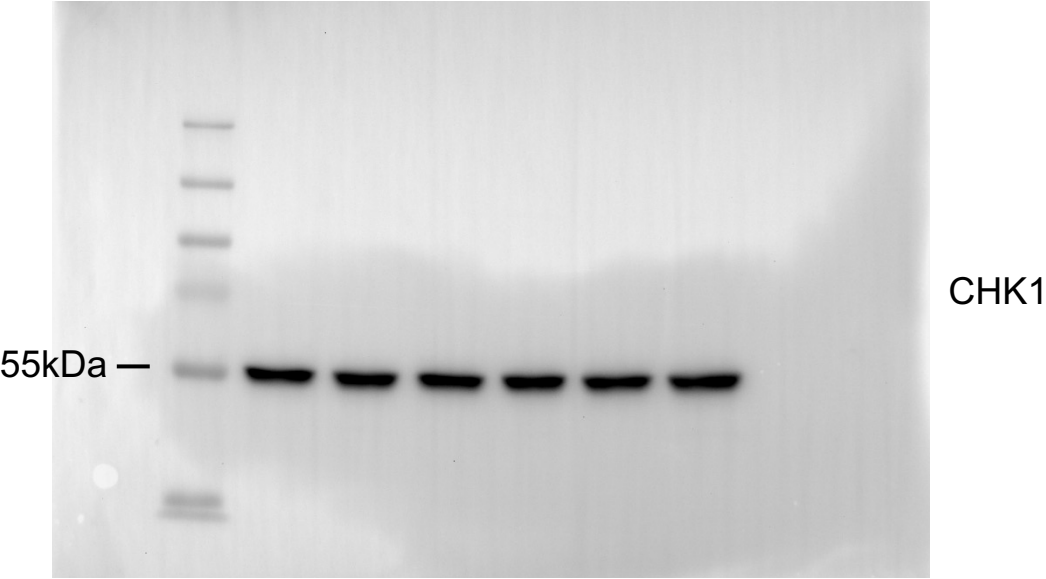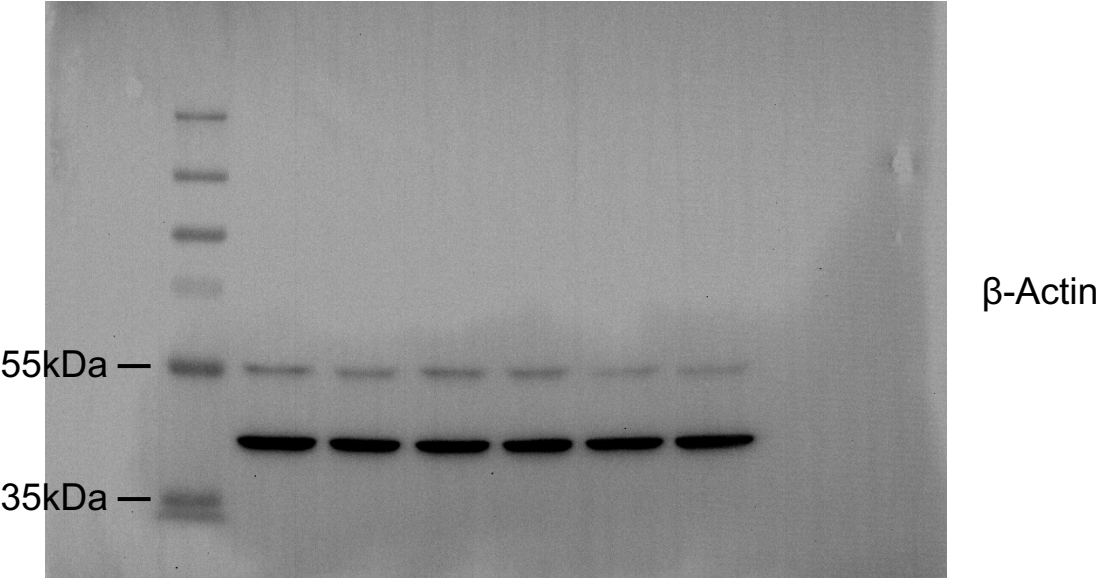

Figure 7D

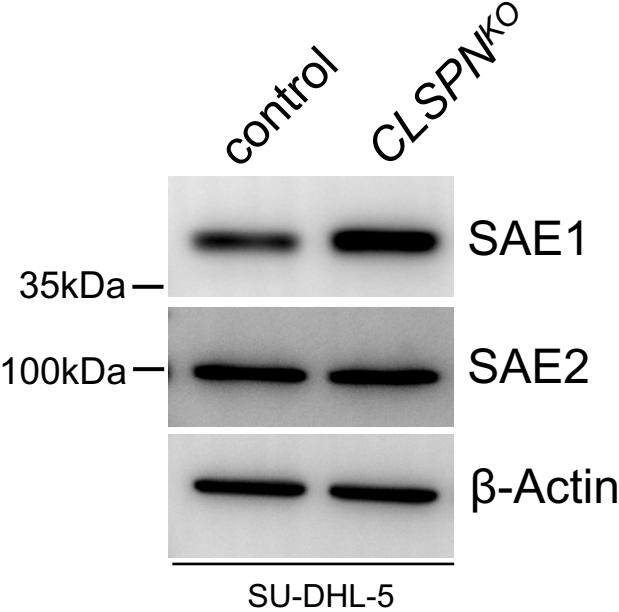

Figure 7D

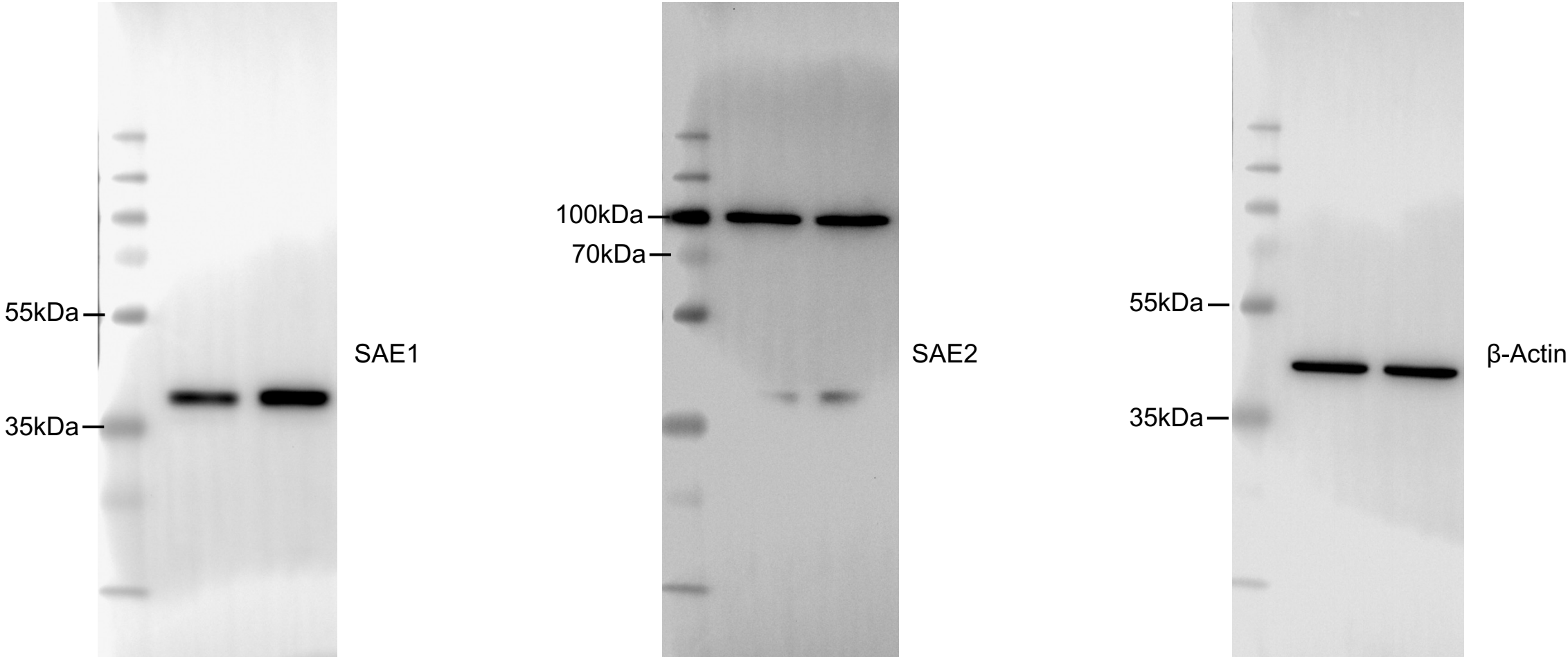

Supplement: Supplementary file 11 — Source Data for Figure 7 [file EMMM-15-e16431-s006.pdf]
