# Supplementary material for: Actionable loss of SLF2 drives B‐cell lymphomagenesis and impairs the DNA damage response
Source: EMBO Mol Med. 2023 Jul 24;15(9):e16431. doi: 10.15252/emmm.202216431 (PMC10493575; doi:10.15252/emmm.202216431)
Supplement: Supplementary file 8 — Source Data for Figure 4 [file EMMM-15-e16431-s007.pdf]

Figure 4A

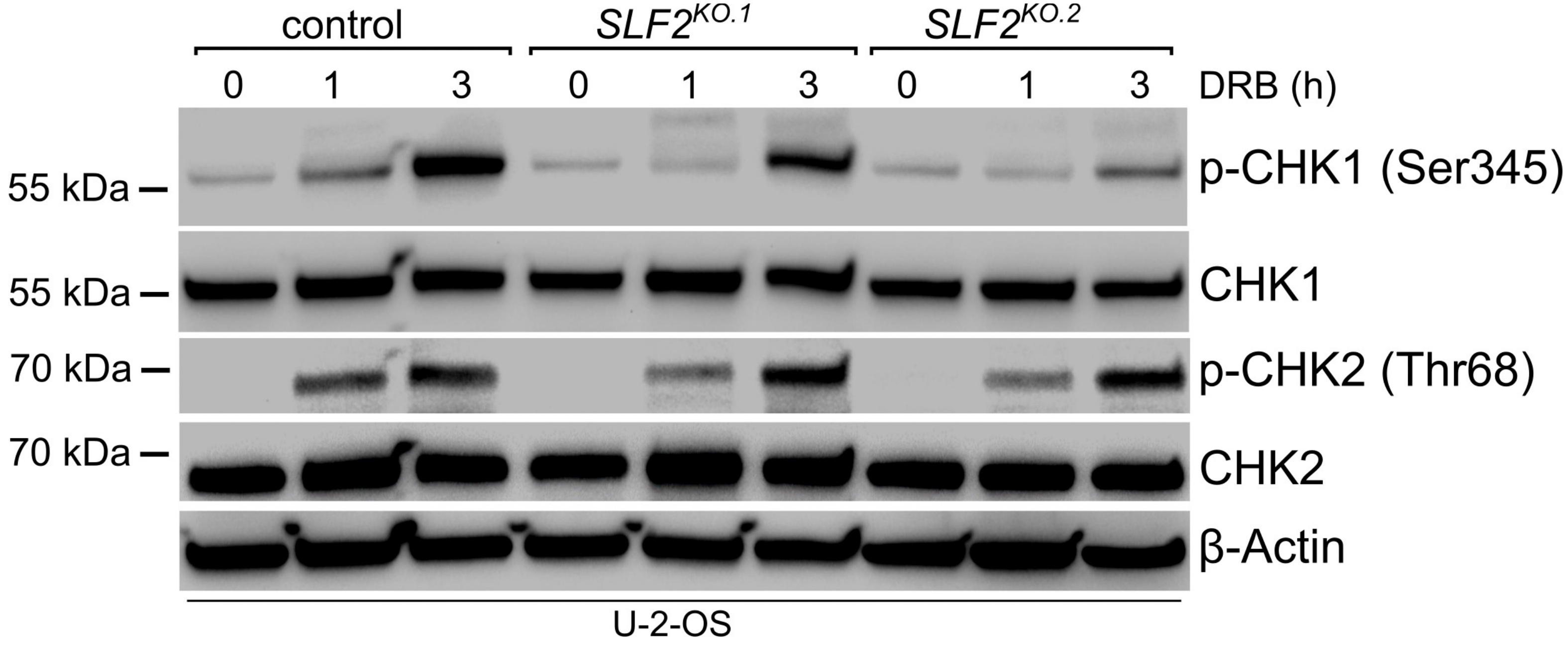
control
SLF2KO.1
SLF2KO.2
0 1 3 0 1 3 0 1 3 DRB (h)
55 kDa
p-CHK1 (Ser345)
55 kDa
CHK1
70 kDa
p-CHK2 (Thr68)
70 kDa
CHK2
β-Actin
U-2-OS

Figure 4A

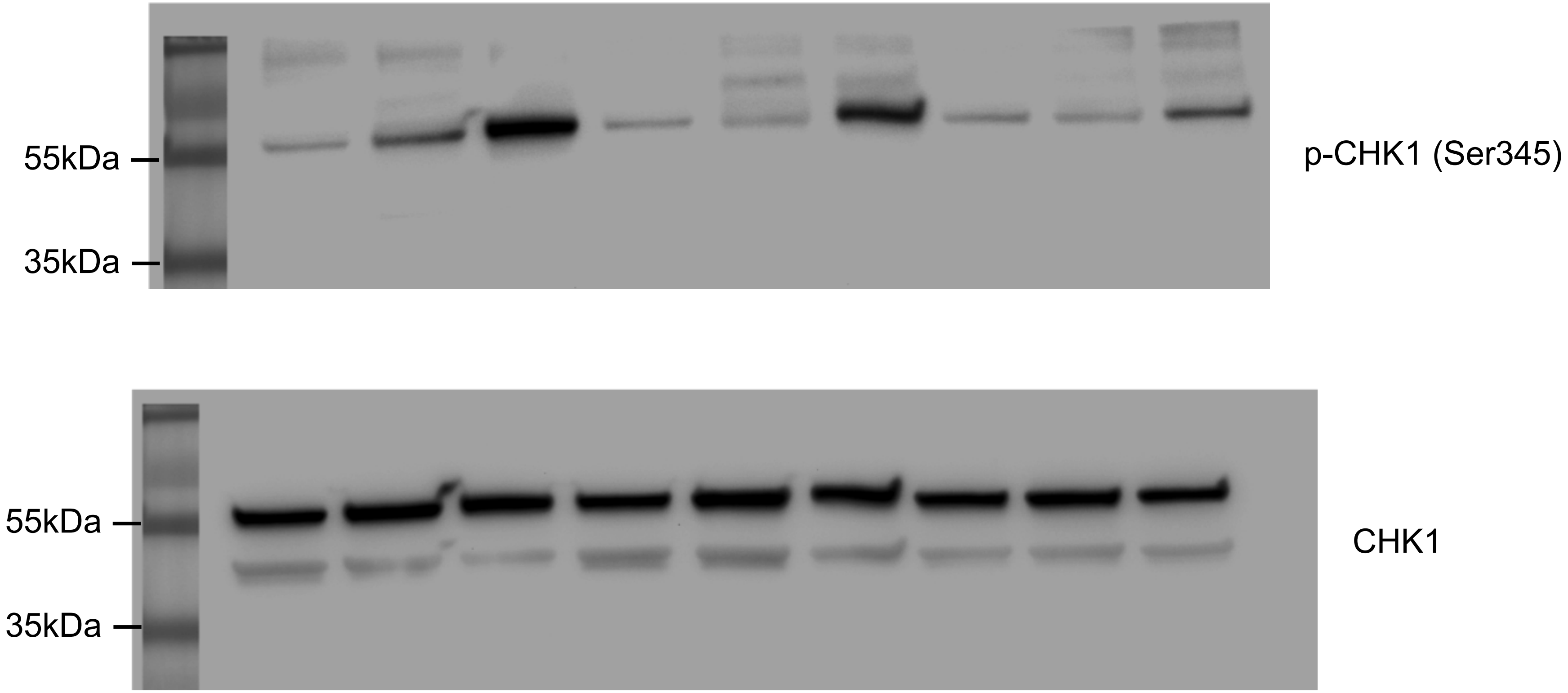

Figure 4A

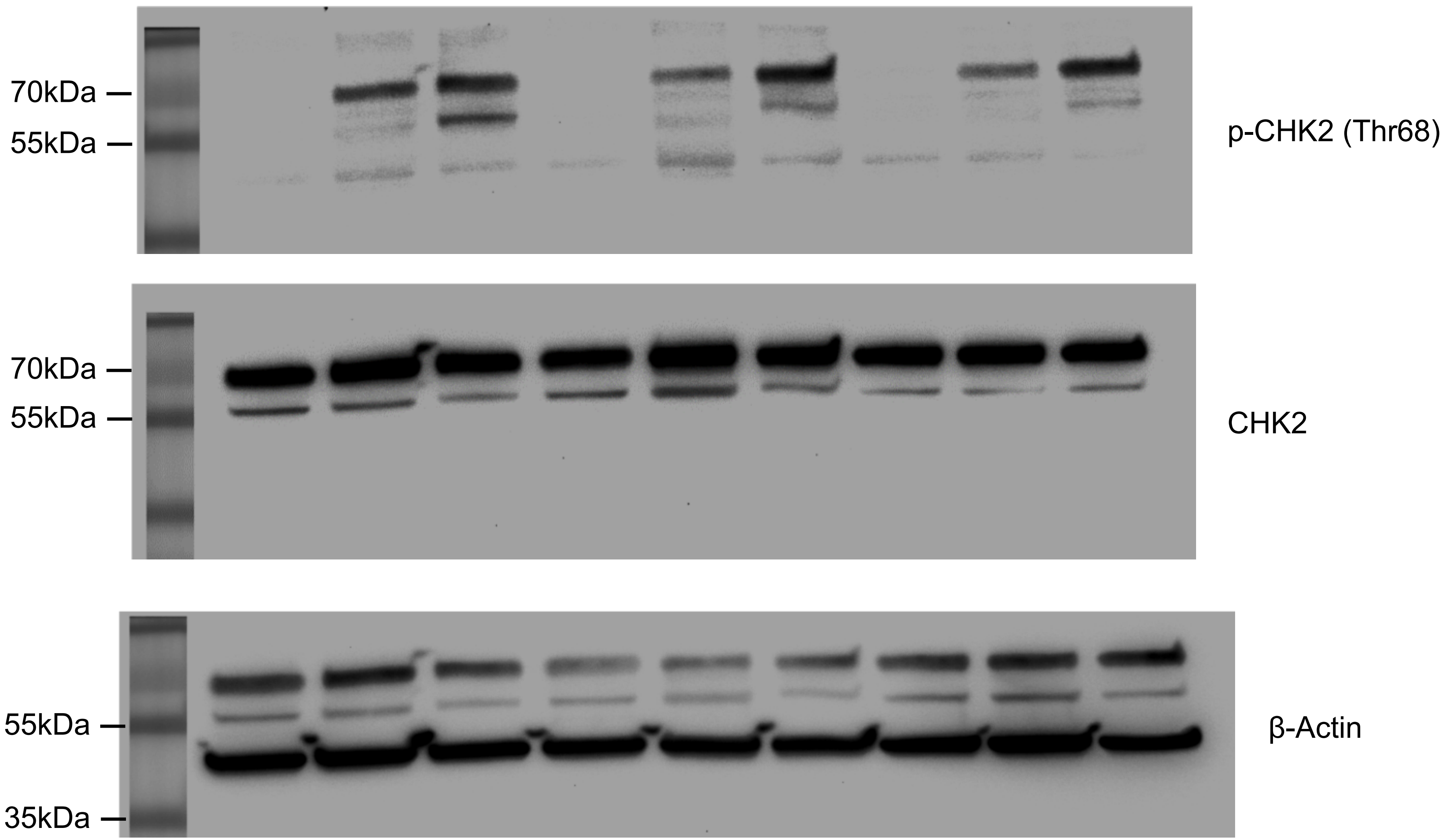

70kDa
55kDa
p-CHK2 (Thr68)
70kDa
55kDa
CHK2
55kDa
35kDa
β-Actin

Figure 4B

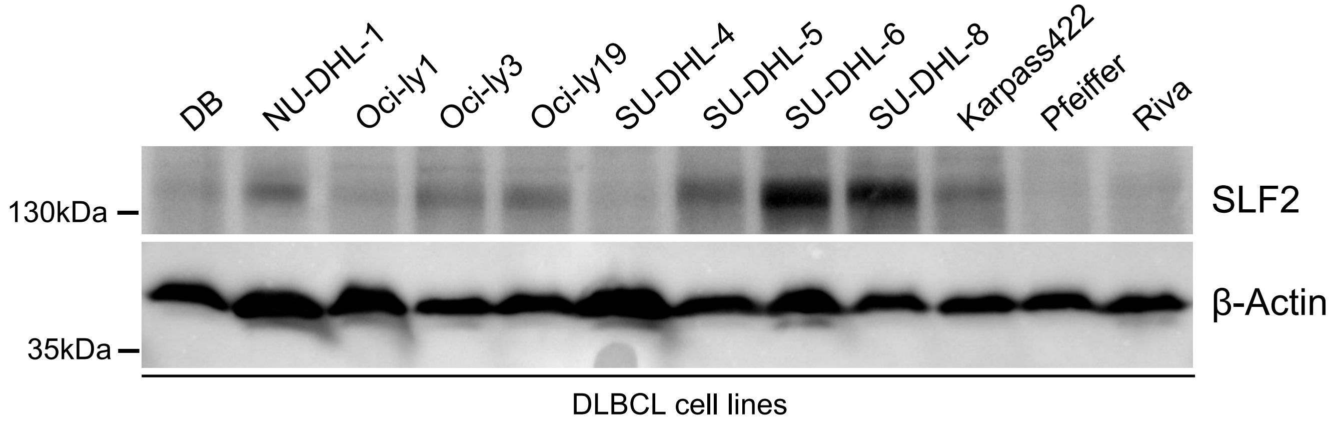
DB
NU-DHL-1
Oci-ly1
Oci-ly3
Oci-ly19
SU-DHL-4
SU-DHL-5
SU-DHL-6
SU-DHL-8
Karpass422
Pfeiffer
Riva
130kDa
SLF2
β-Actin
35kDa
DLBCL cell lines

Figure 4B

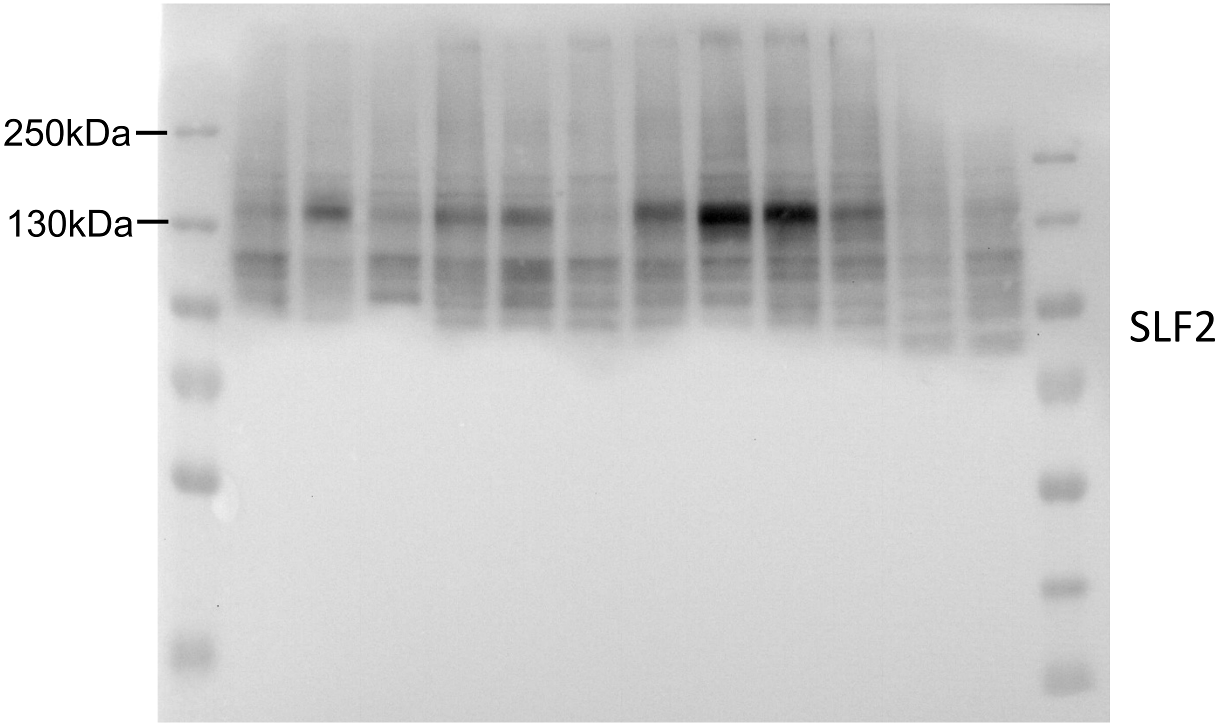

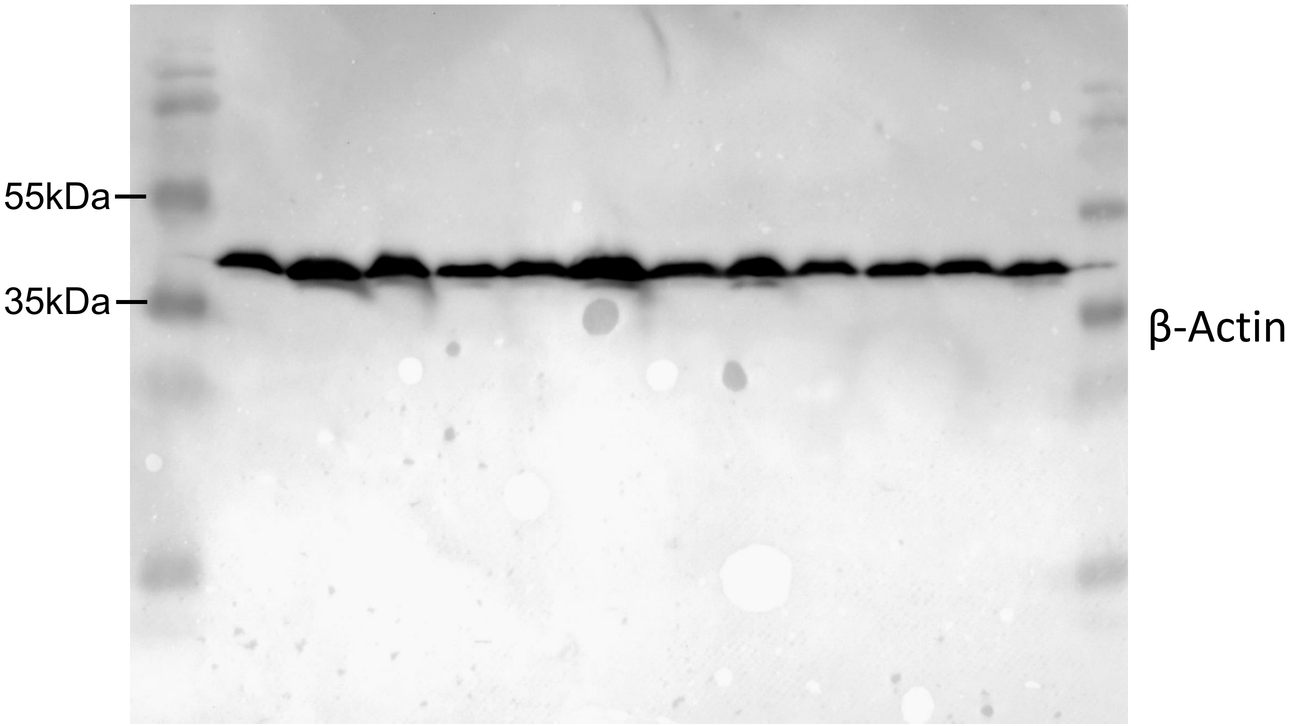

Figure 4C

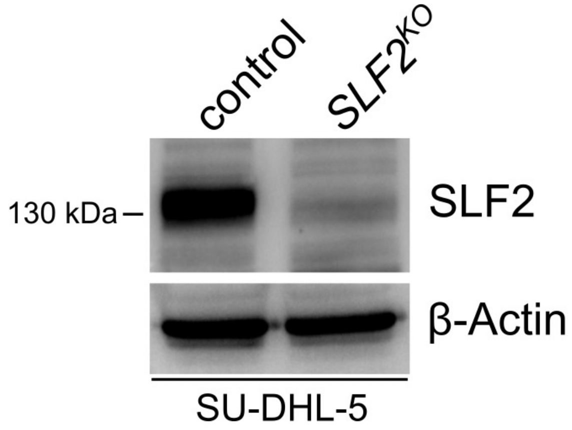

Figure 4C

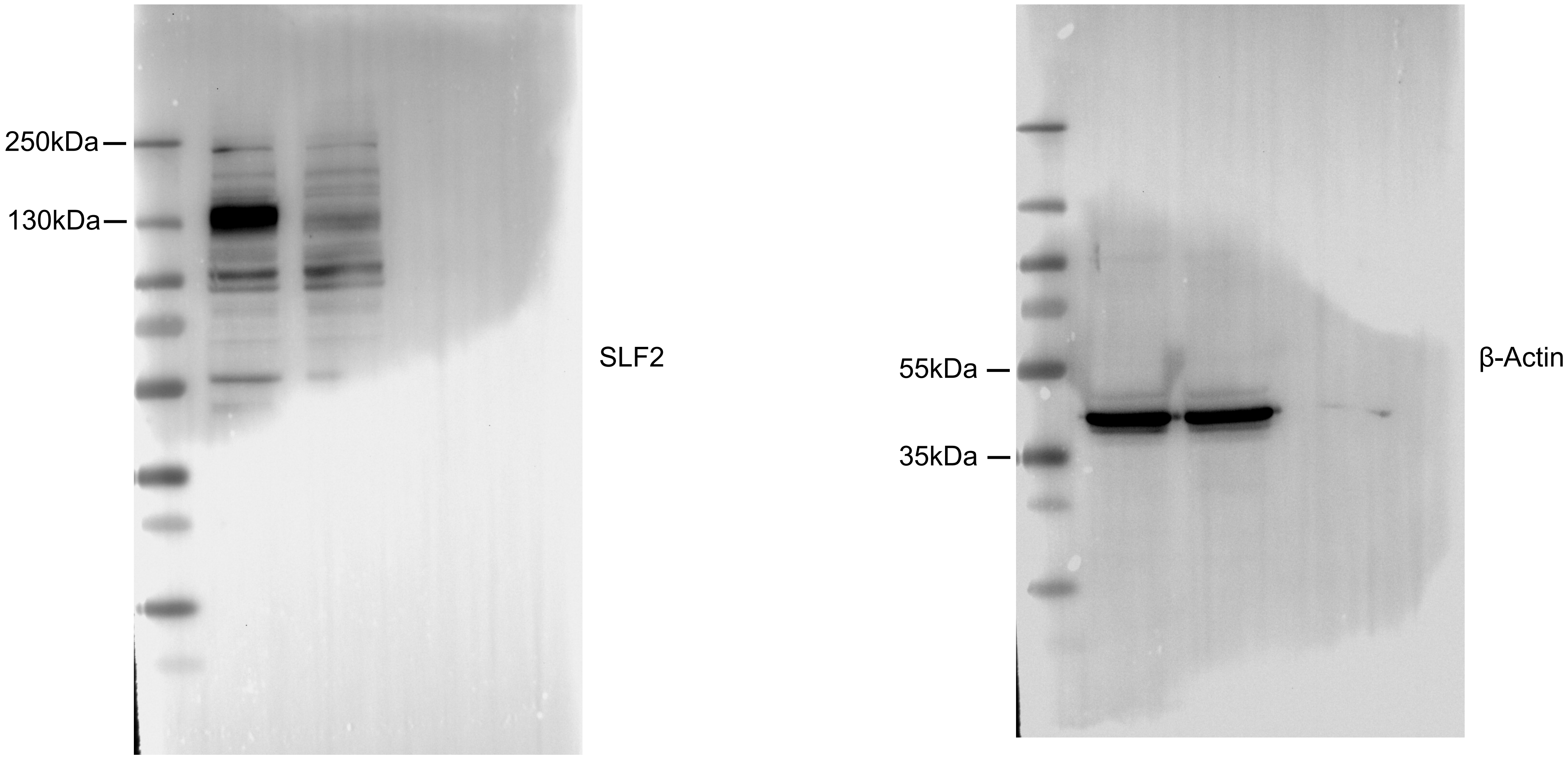

Figure 4E

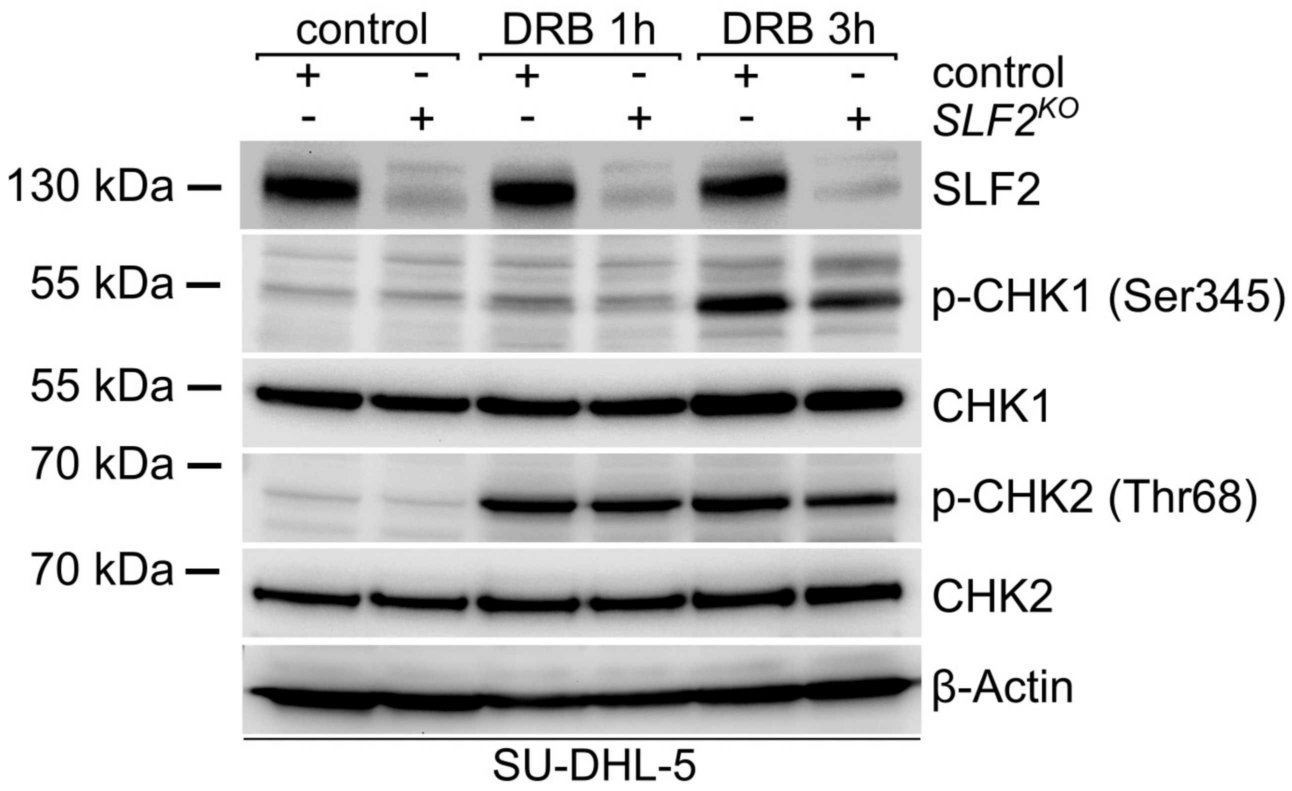
control
DRB 1h
DRB 3h
+ - + - + -
control
- + - + - +
SLF2KO
130 kDa
55 kDa
55 kDa
70 kDa
70 kDa
SLF2
p-CHK1 (Ser345)
CHK1
p-CHK2 (Thr68)
CHK2
β-Actin
SU-DHL-5

Figure 4E

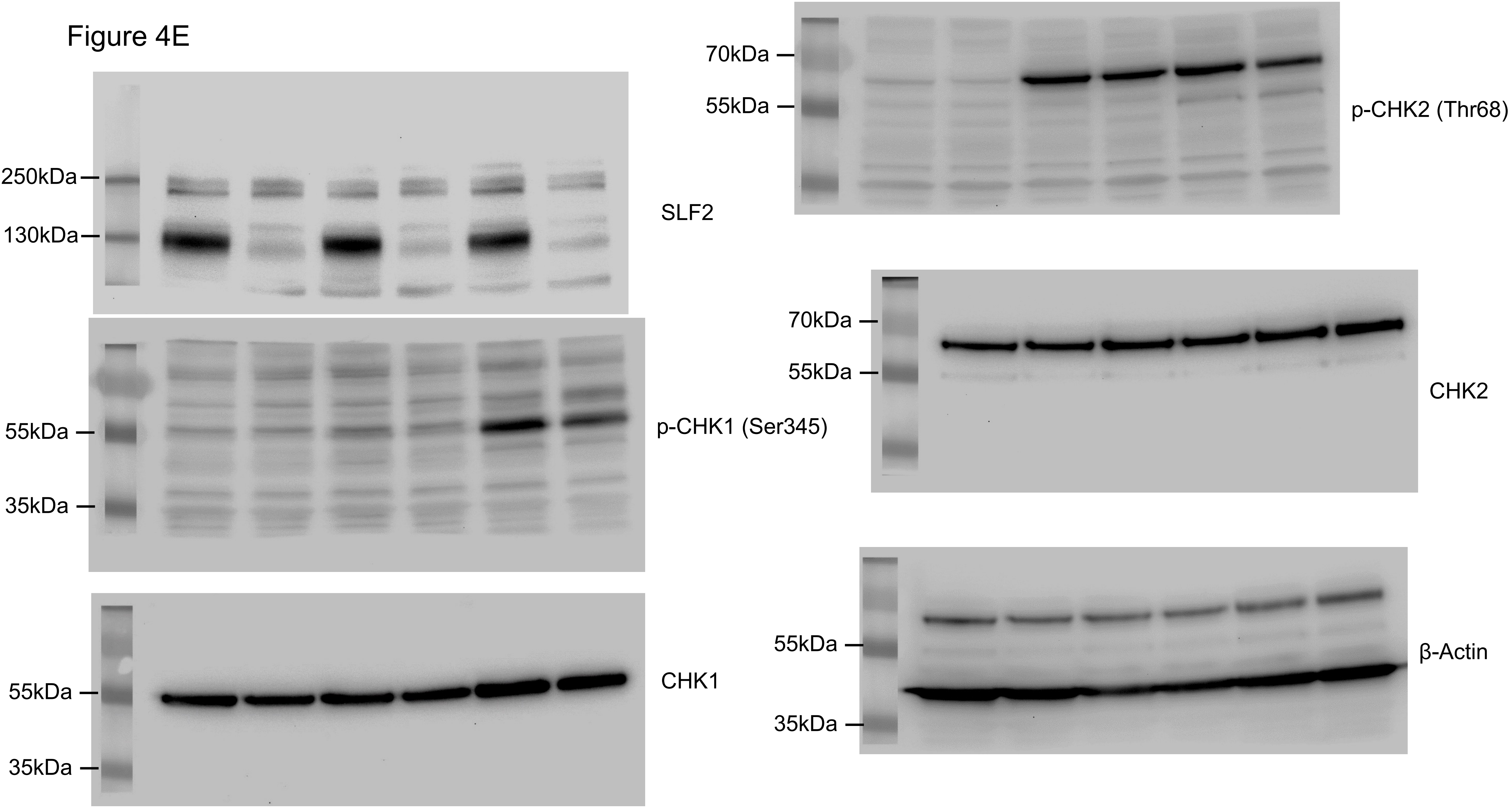
250kDa
130kDa
SLF2
55kDa
35kDa
p-CHK1 (Ser345)
55kDa
35kDa
CHK1
70kDa
55kDa
p-CHK2 (Thr68)
70kDa
55kDa
CHK2
55kDa
35kDa
β-Actin

Figure 4G

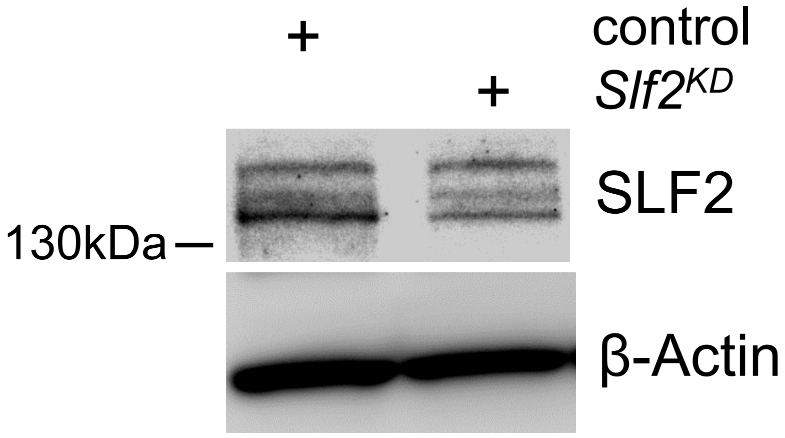

Figure 4G

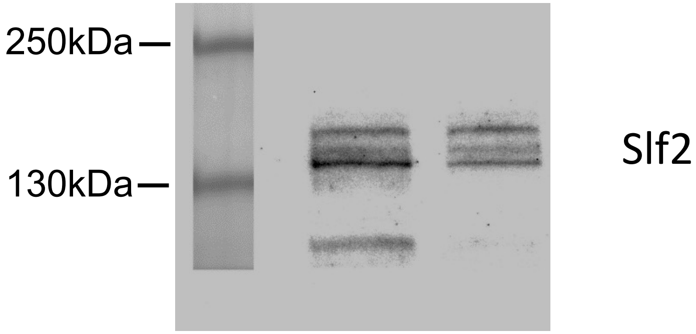

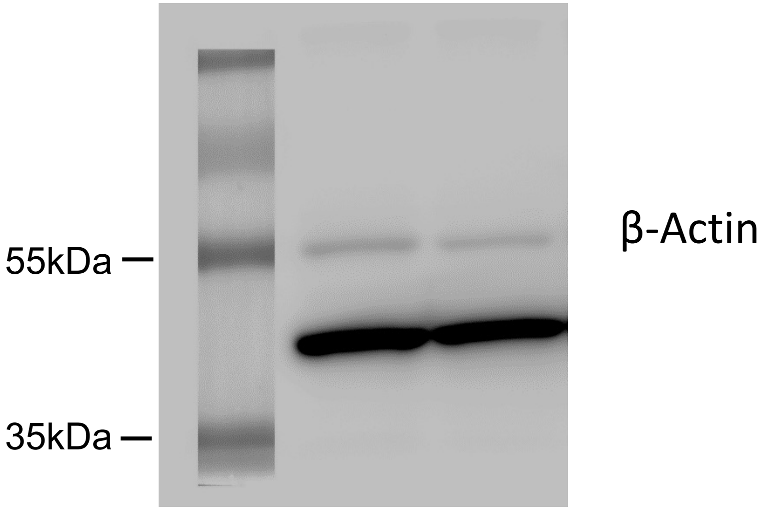

Figure 4I

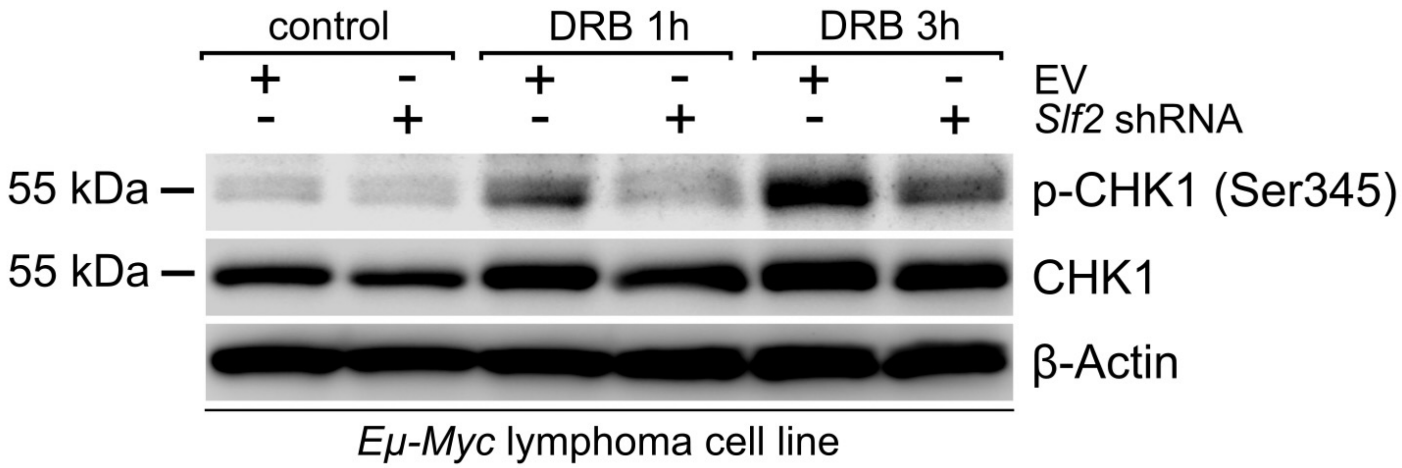
control
DRB 1h
DRB 3h
EV
Slf2 shRNA
+ - + - + -
- + - + - +
55 kDa
55 kDa
p-CHK1 (Ser345)
CHK1
β-Actin
Eμ-Myc lymphoma cell line

Figure 4I

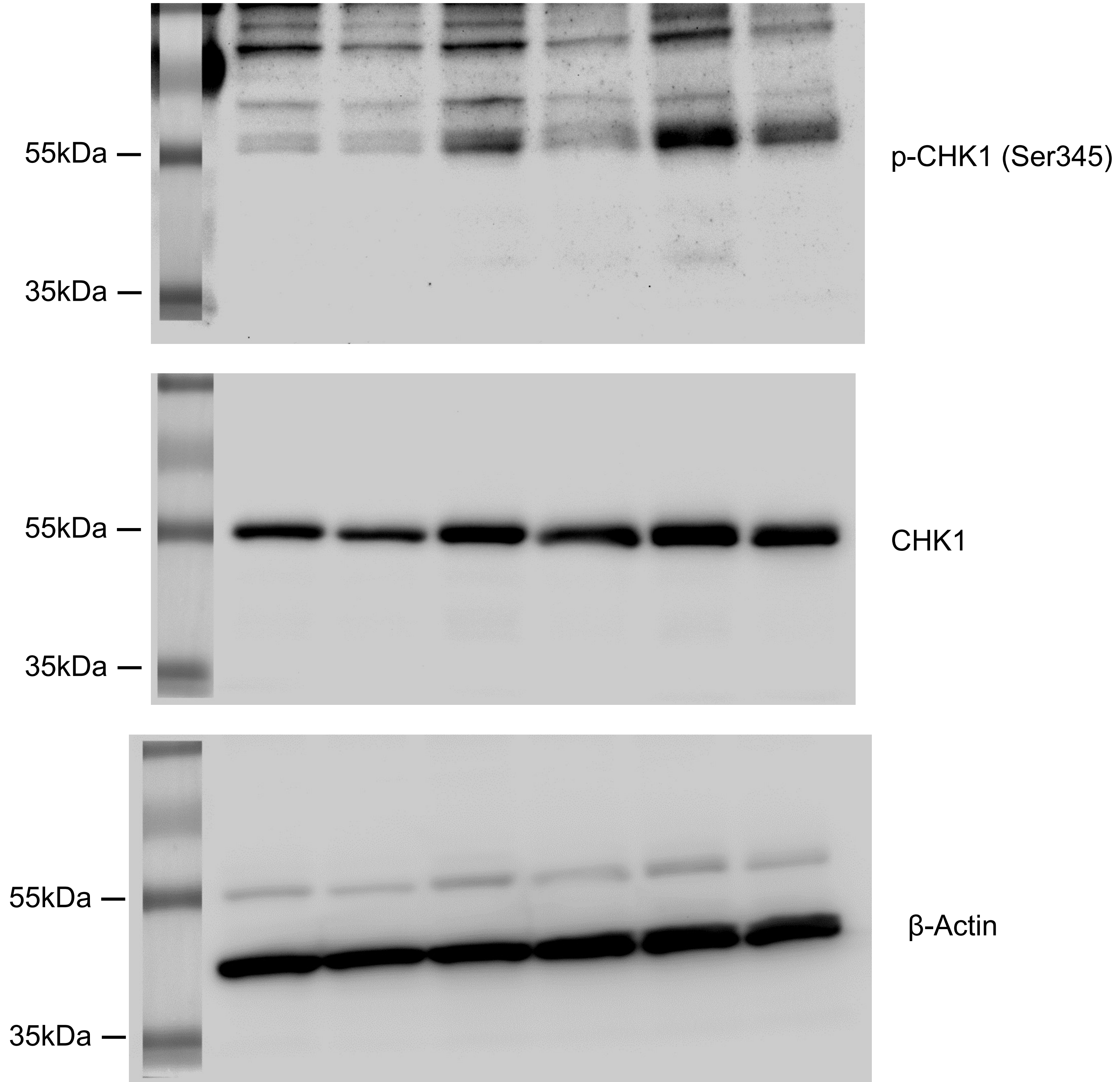
55kDa
35kDa
p-CHK1 (Ser345)
55kDa
35kDa
CHK1
55kDa
35kDa
β-Actin
