# Supplementary material for: Actionable loss of SLF2 drives B‐cell lymphomagenesis and impairs the DNA damage response
Source: EMBO Mol Med. 2023 Jul 24;15(9):e16431. doi: 10.15252/emmm.202216431 (PMC10493575; doi:10.15252/emmm.202216431)
Supplement: Supplementary file 9 — Source Data for Figure 5 [file EMMM-15-e16431-s010.pdf]

Main Figure 5B and Appendix Figure S12I

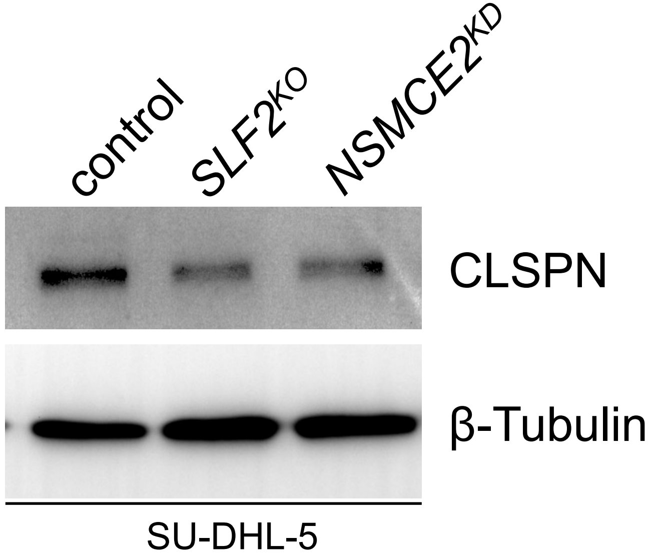

Main Figure 5B and Appendix Figure S12I

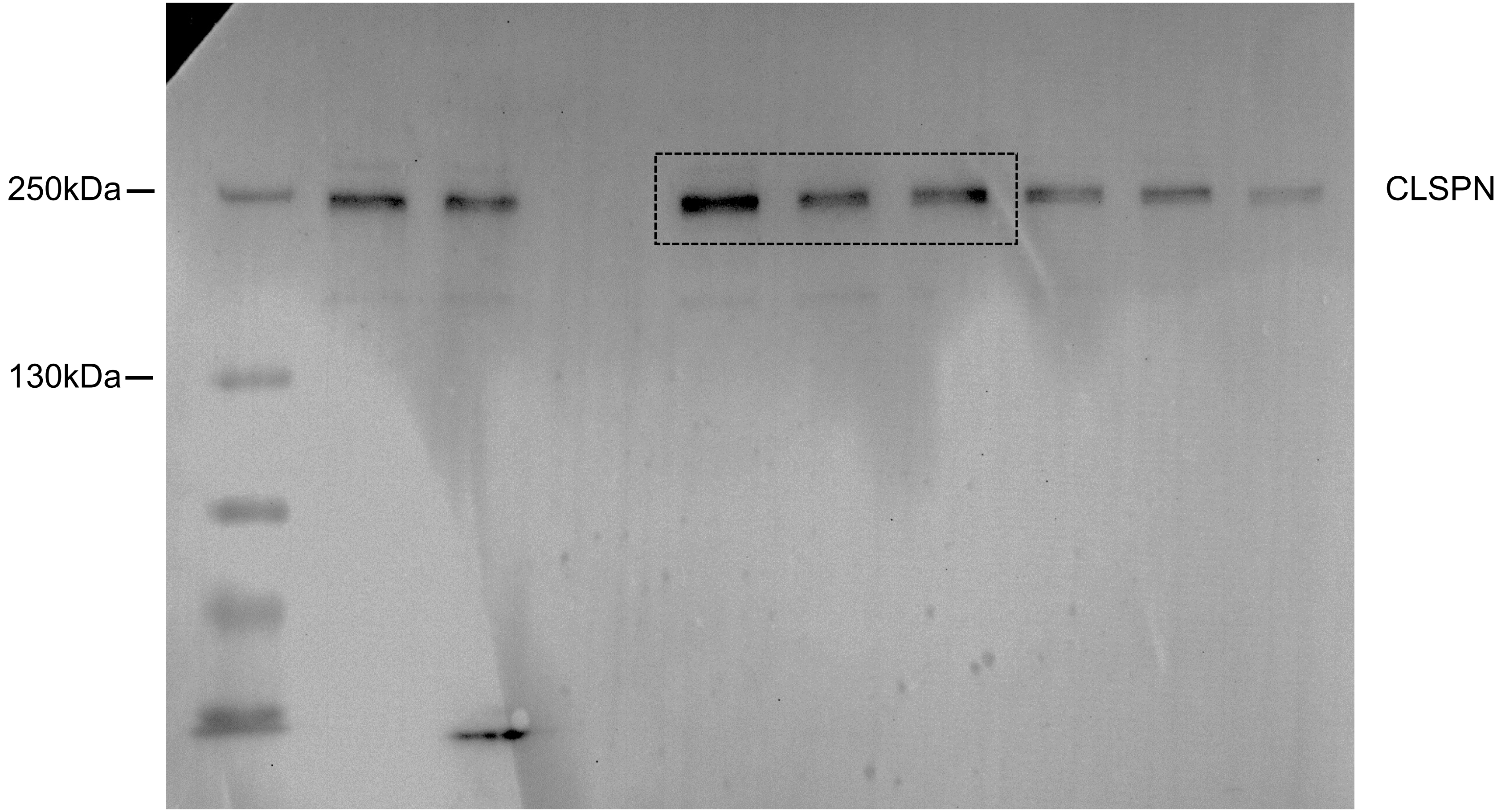

Main Figure 5B and Appendix Figure S12I

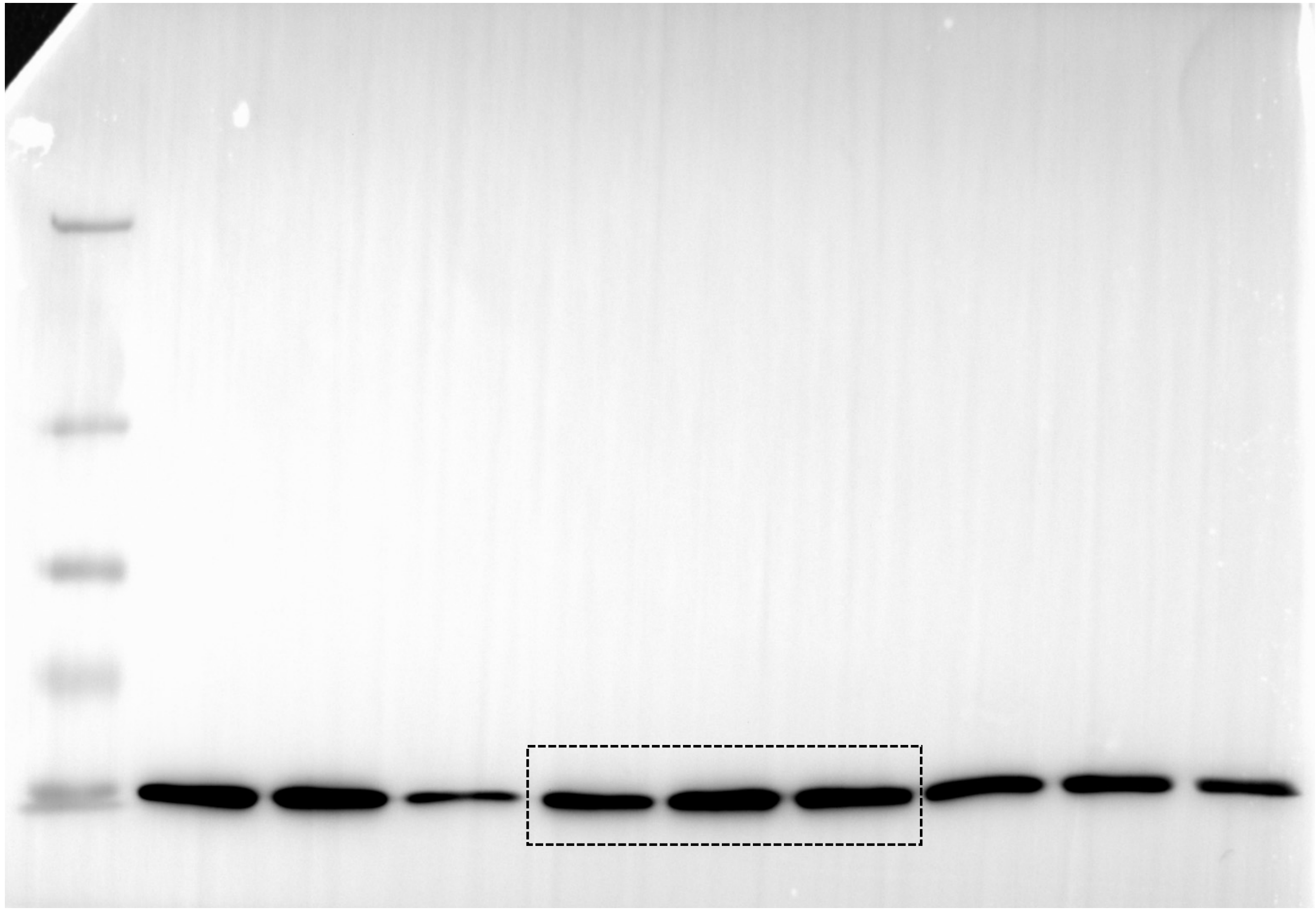

Figure 5E

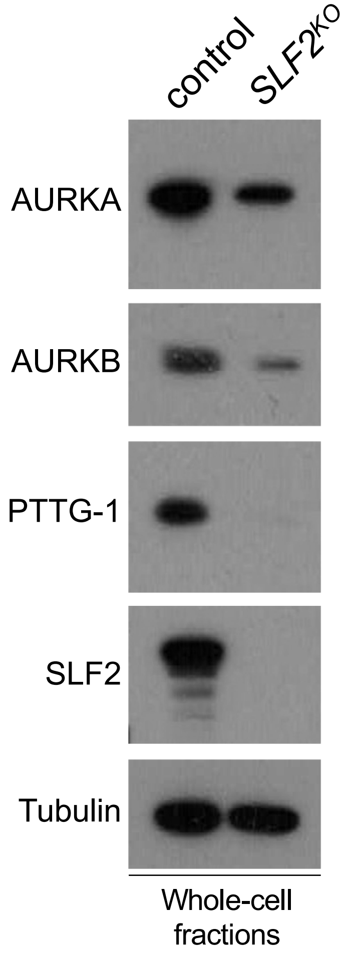
control
SLF2KO
AURKA
AURKB
PTTG-1
SLF2
Tubulin
Whole-cell fractions

Figure 5E

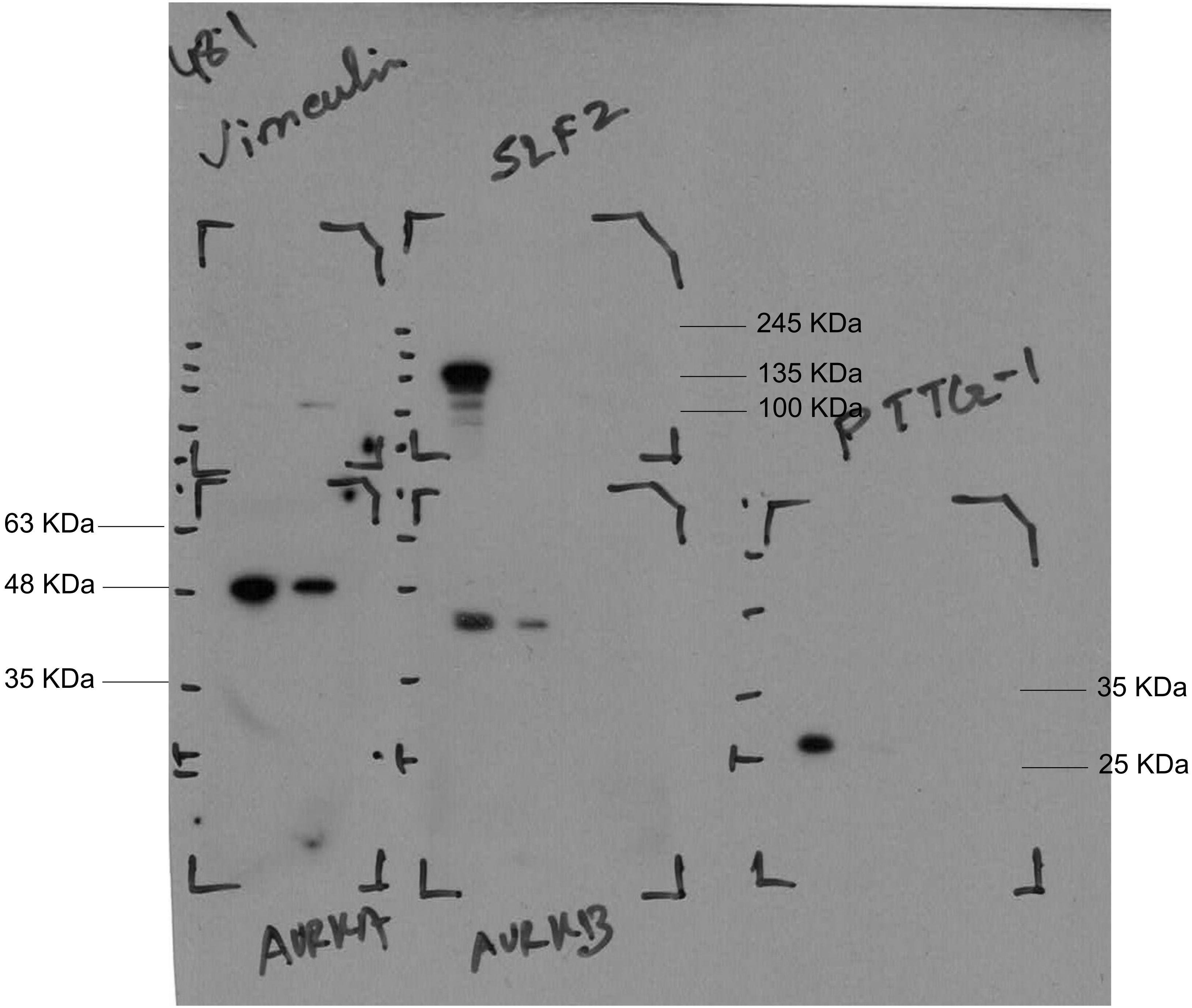

4B1
Vimculin
SLF2
245 KDa
135 KDa
100 KDa
PTTG-1
63 KDa
48 KDa
35 KDa
35 KDa
25 KDa
AURKA
AURKB

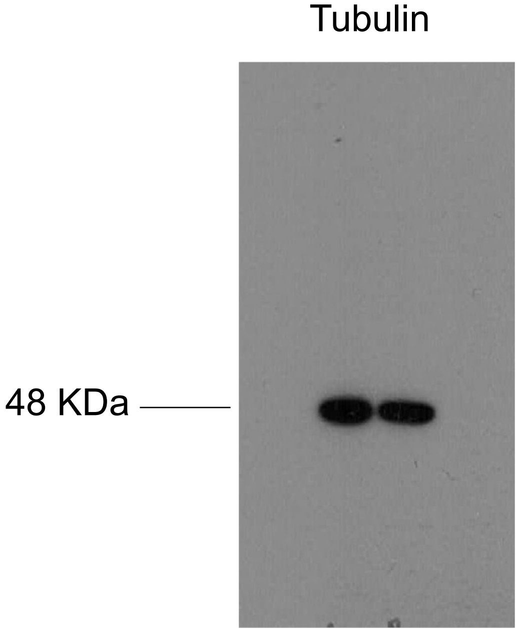

Tubulin
48 KDa

Figure 5F

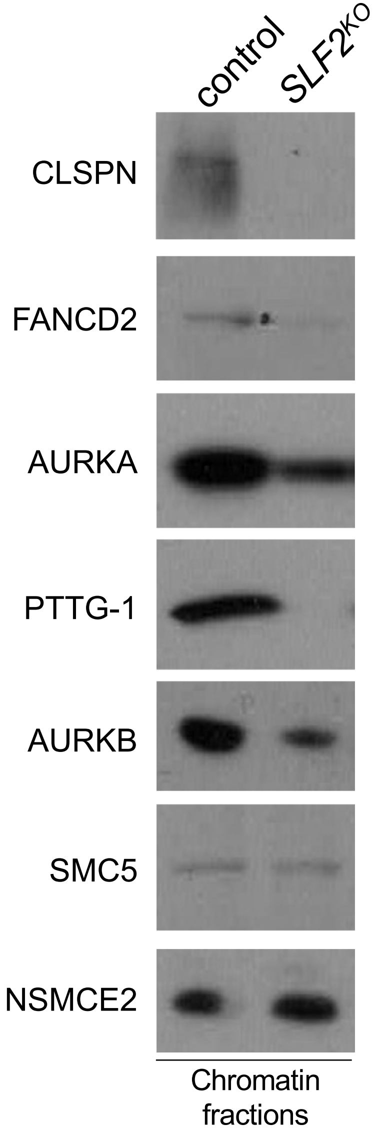
control
SLF2KO
CLSPN
FANCD2
AURKA
PTTG-1
AURKB
SMC5
NSMCE2
Chromatin fractions

Figure 5F

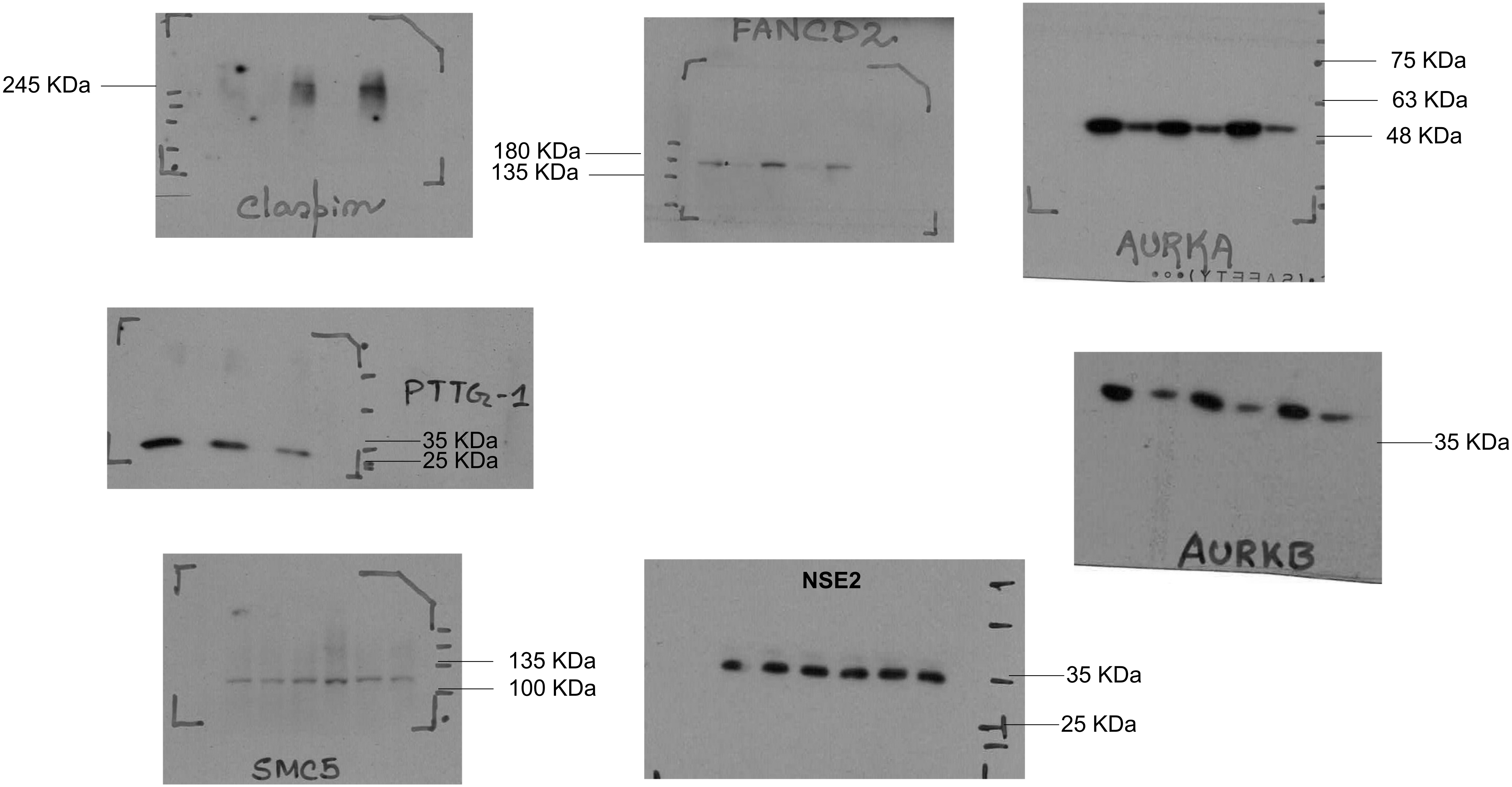

245 KDa
claspin
FANCD2
180 KDa
135 KDa
75 KDa
63 KDa
48 KDa
AURKA
PTTG-1
35 KDa
25 KDa
35 KDa
AURKB
135 KDa
100 KDa
SMC5
NSE2
35 KDa
25 KDa
