# Supplementary material for: Actionable loss of SLF2 drives B‐cell lymphomagenesis and impairs the DNA damage response
Source: EMBO Mol Med. 2023 Jul 24;15(9):e16431. doi: 10.15252/emmm.202216431 (PMC10493575; doi:10.15252/emmm.202216431)
Supplement: Supplementary file 12 [file EMMM-15-e16431-s002.pdf]

EMBO Molecular Medicine

Actionable loss of SLF2 drives B-cell lymphomagenesis and impairs the DNA damage response

Le Zhang, Matthias Wirth, Upayan Patra, Jacob Stroh, Konstandina Isaakidis, Leonie Rieger, Susanne Kossatz, Maja Milanovic, Chuanbing Zang, Uta Demel, Jan Keiten-Schmitz, Kristina Wagner, Katja Steiger, Roland Rad, Florian Bassermann, Stefan Müller, Ulrich Keller, and Markus Schick
DOI: 10.15252/emmm.202216431

Corresponding author(s): Markus Schick (markus.schick@charite.de) , Ulrich Keller (ulrich.keller@charite.de)

Review Timeline:

Submission Date:	10th Jun 22
Editorial Decision:	28th Jun 22
Revision Received:	14th May 23
Editorial Decision:	9th Jun 23
Revision Received:	2nd Jul 23
Accepted:	6th Jul 23

Editor: Lise Roth

Transaction Report:

(Note: With the exception of the correction of typographical or spelling errors that could be a source of ambiguity, letters and reports are not edited. Depending on transfer agreements, referee reports obtained elsewhere may or may not be included in this compilation. Referee reports are anonymous unless the Referee chooses to sign their reports.)

28th Jun 2022

Dear Dr. Schick,

Thank you for the submission of your manuscript to EMBO Molecular Medicine. We have now received feedback from the three reviewers who agreed to evaluate your manuscript. As you will see from the reports below, they all mention the potential interest of the study, but also raise a number of partially overlapping concerns, mostly related to the need to strengthen the mechanistic insight. Furthermore, referee #3 asked for additional in vivo experiments to demonstrate drug synergy in a mouse B-cell lymphoma model.
While we understand this might be time-consuming, we agree this experiment is important to substantiate the clinical relevance of the findings, and are therefore willing to extend the revision time to 6 months.
Please let us know if you would like to discuss the revision further.

Addressing the reviewers' concerns in full will be necessary for further considering the manuscript in our journal, and acceptance of the manuscript will entail a second round of review. EMBO Molecular Medicine encourages a single round of revision only and therefore, acceptance or rejection of the manuscript will depend on the completeness of your responses included in the next, final version of the manuscript. For this reason, and to save you from any frustrations in the end, I would strongly advise against returning an incomplete revision.

When submitting your revised manuscript, please carefully review the instructions that follow below. We perform an initial quality control of all revised manuscripts before re-review; failure to include requested items will delay the evaluation of your revision.

We require:

1) A .docx formatted version of the manuscript text (including legends for main figures, EV figures and tables). Please make sure that the changes are highlighted to be clearly visible.

2) Individual production quality figure files as .eps, .tif, .jpg (one file per figure). For guidance, download the 'Figure Guide PDF' (https://www.embopress.org/page/journal/17574684/authorguide#figureformat).

3) A .docx formatted letter INCLUDING the reviewers' reports and your detailed point-by-point responses to their comments. As part of the EMBO Press transparent editorial process, the point-by-point response is part of the Review Process File (RPF), which will be published alongside your paper.

4) A complete author checklist, which you can download from our author guidelines (https://www.embopress.org/page/journal/17574684/authorguide#submissionofrevisions). Please insert information in the checklist that is also reflected in the manuscript. The completed author checklist will also be part of the RPF.

5) Please note that all corresponding authors are required to supply an ORCID ID for their name upon submission of a revised manuscript.

6) It is mandatory to include a 'Data Availability' section after the Materials and Methods. Before submitting your revision, primary datasets produced in this study need to be deposited in an appropriate public database, and the accession numbers and database listed under 'Data Availability'. Please remember to provide a reviewer password if the datasets are not yet public (see https://www.embopress.org/page/journal/17574684/authorguide#dataavailability).
In case you have no data that requires deposition in a public database, please state so in this section. Note that the Data Availability Section is restricted to new primary data that are part of this study.

7) For data quantification: please specify the name of the statistical test used to generate error bars and P values, the number (n) of independent experiments (specify technical or biological replicates) underlying each data point and the test used to calculate p-values in each figure legend. The figure legends should contain a basic description of n, P and the test applied. Graphs must include a description of the bars and the error bars (s.d., s.e.m.). Please indicate the exact p values, not a range.

8) We would also encourage you to include the source data for figure panels that show essential data. Numerical data should be provided as individual .xls or .csv files (including a tab describing the data). For blots or microscopy, uncropped images should be submitted (using a zip archive if multiple images need to be supplied for one panel). Additional information on source data and instruction on how to label the files are available at
.

9) Our journal encourages inclusion of *data citations in the reference list* to directly cite datasets that were re-used and

obtained from public databases. Data citations in the article text are distinct from normal bibliographical citations and should directly link to the database records from which the data can be accessed. In the main text, data citations are formatted as follows: "Data ref: Smith et al, 2001" or "Data ref: NCBI Sequence Read Archive PRJNA342805, 2017". In the Reference list, data citations must be labeled with "[DATASET]". A data reference must provide the database name, accession number/identifiers and a resolvable link to the landing page from which the data can be accessed at the end of the reference. Further instructions are available at .

10) We replaced Supplementary Information with Expanded View (EV) Figures and Tables that are collapsible/expandable online. A maximum of 5 EV Figures can be typeset. EV Figures should be cited as 'Figure EV1, Figure EV2" etc... in the text and their respective legends should be included in the main text after the legends of regular figures.
- For the figures that you do NOT wish to display as Expanded View figures, they should be bundled together with their legends in a single PDF file called *Appendix*, which should start with a short Table of Content. Appendix figures should be referred to in the main text as: "Appendix Figure S1, Appendix Figure S2" etc.
- Additional Tables/Datasets should be labeled and referred to as Table EV1, Dataset EV1, etc. Legends have to be provided in a separate tab in case of .xls files. Alternatively, the legend can be supplied as a separate text file (README) and zipped together with the Table/Dataset file.
See detailed instructions here:
.

11) The paper explained: EMBO Molecular Medicine articles are accompanied by a summary of the articles to emphasize the major findings in the paper and their medical implications for the non-specialist reader. Please provide a draft summary of your article highlighting
- the medical issue you are addressing,
- the results obtained and
- their clinical impact.

This may be edited to ensure that readers understand the significance and context of the research. Please refer to any of our published articles for an example.

12) For more information: There is space at the end of each article to list relevant web links for further consultation by our readers. Could you identify some relevant ones and provide such information as well? Some examples are patient associations, relevant databases, OMIM/proteins/genes links, author's websites, etc...

13) Author contributions: the contribution of every author must be detailed in a separate section (before the acknowledgments).

14) Conflict of interest: We updated our journal's competing interests policy in January 2022 and request authors to consider both actual and perceived competing interests. Please review the policy https://www.embopress.org/competing-interests and update your competing interests if necessary.

15) Every published paper now includes a 'Synopsis' to further enhance discoverability. Synopses are displayed on the journal webpage and are freely accessible to all readers. They include a short stand first (maximum of 300 characters, including space) as well as 2-5 one-sentences bullet points that summarizes the paper. Please write the bullet points to summarize the key NEW findings. They should be designed to be complementary to the abstract - i.e. not repeat the same text. We encourage inclusion of key acronyms and quantitative information (maximum of 30 words / bullet point). Please use the passive voice. Please attach these in a separate file or send them by email, we will incorporate them accordingly.

Please also suggest a striking image or visual abstract to illustrate your article as a PNG file 550 px wide x 300-600 px high.

16) As part of the EMBO Publications transparent editorial process initiative (see our Editorial at http://embomolmed.embopress.org/content/2/9/329), EMBO Molecular Medicine will publish online a Review Process File (RPF) to accompany accepted manuscripts.
In the event of acceptance, this file will be published in conjunction with your paper and will include the anonymous referee reports, your point-by-point response and all pertinent correspondence relating to the manuscript. Let us know whether you agree with the publication of the RPF and as here, if you want to remove or not any figures from it prior to publication.
Please note that the Authors checklist will be published at the end of the RPF.

EMBO Molecular Medicine has a "scooping protection" policy, whereby similar findings that are published by others during review or revision are not a criterion for rejection. Should you decide to submit a revised version, I do ask that you get in touch after three months if you have not completed it, to update us on the status.

I look forward to receiving your revised manuscript.

Yours sincerely,

Lise Roth

Lise Roth, PhD
Senior Editor
EMBO Molecular Medicine

***** Reviewer's comments *****

Referee #1 (Remarks for Author):

Zhang and co-workers identified SLF2 as a DDR pathway regulator by modulating ATR-CLSPN-CHK1 axis, which is associated with MYC-driven B cell lymphoma. The authors elegantly showed several lines of evidence of SLF2 as a tumor suppressor in association with DLBCL. The authors claimed that a loss of SLF2 associated with a high level of DNA damage could activate sumoylation pathway as a safeguard. Therefore, they proposed co-targeting of the sumoylation and DDR pathways is a promising strategy for the treatment of aggressive lymphoma. Several reports have demonstrated sumoylation pathway is a synthetic lethal partner for oncogenes such as MYC and RAS. Previous studies have also shown SLF2 as a player of the DDR. Therefore, co-targeting of the DDR and sumoylation for the treatment of MYC-driven lymphoma is not conceptually advanced. The main novelty of this study is the identification of SLF2 as a biomarker for B-cell lymphoma and SLF2 in regulating the DDR pathway via CLSPN. While the current format of this manuscript showed the biological significance of SLF2 in the DDR pathway, the mechanistic insight as to how SLF2 modulates CLSPN levels and how SLF2 deficiency leads to an aberrant sumoylation signature (globally or mainly the key factors associated with DDR pathway) should be addressed to increase the cohesiveness and novelty of this study. Several suggestions to improve the quality of this manuscript are as follows:
Major

1. The current data provided association of SLF2 in DNA repair, MYC-driven B cell lymphomagenesis, and requirement for ATR-CLSPN-CHK1 axis. It is not clear how SLF2 alters CLSPN levels. The mechanistic study should be included to enhance the novelty of this study.
2. The authors claimed that NSMCE2 is a SUMO E3 ligase able to form complexes with SLF2 and NSMCE2 deficient cells also showed impairment of CHK1 activation. Does NSCMCE2 also affect CLSPN levels? Is SLF2 deficiency-elicited any sumoylation change due to NSMCE2? Likewise, does NSMCE2 deficiency alter sumoylation associated with the DDR pathway? Since NSMCE2 could suppress cancer independently of its SUMO ligase activity (EMBO J 2015, 34(21):2604-2619), it should be clarified the issue of NSMCE2 in sumoylation regulation and the interplay among SLF2, NSCMCE2, and sumoylation change. Whether such sumoylation alteration is associated with SLF2 deficiency-elicited aberrant sumoylation signature should be addressed.

Minor
1. Dysregulated genes involved in lymphomas and wild-type B cells should be presented by heatmap in Fig. 1a and 1b.
2. The biological effect of the other candidates (Fig. 1e) should be demonstrated as Fig 1h.
3. In Fig. 1h, the DAPI images in control set need to be fixed (upside down).
4. In Fig. 4d, the authors claimed that CHK2 activation was not altered by SLF2 depletion. However, the Western blot showed a slight reduction of phospho-CHK2 in SLF-KO cells. Quantification of these band intensities may help to resolve this issue.
5. Why SLF2 exhibited two bands in Western blots?
6. What is the correlation between SLF2 levels, cell proliferation / DNA damage foci number, and CLSPN levels among the patient-derived DLBCL cell lines (Figure 4b)?
7. Fig. 4f: this is not a quantification mass result.
8. In Fig. 5d-i, the authors didn't indicate the specific concentration of inhibitors.
9. SU-DHL-5 control and SLF2 KO cells should be included in Fig 5h.
10. You should define this abbreviation the first time and then only use the abbreviated. For instance, the abbreviation "DLBCL" was used in the first paragraph of the Introduction without definition.

Referee #2 (Remarks for Author):

In this manuscript, Schick and colleagues identify SLF2 as a tumor suppressor gene in Eμ-myc lymphomas. They also show that SLF2 is suppressed and associated with a worse prognosis in human DLBCL. Given previous reports linking SLF2 to the DNA

damage response (DDR), the Authors investigated whether loss of SLF2 leads to (i) increased genome instability and (ii) alterations in DDR signaling. Among the most salient findings, they show that loss of SLF2 impairs CHK1 activation. They also show that loss of SLF2 sensitizes cells to an inhibitor of sumoylation.

The manuscript has the potential merit of having identified a new tumor suppressor gene in DLBCLs (and possibly other non-Hodgkin lymphomas), provides clues concerning SLF2 molecular function/mechanism, and shows evidence for the selective preclinical targeting of lymphomas characterized by low expression of SLF2.

For the most part, experiments are well structured and executed, and the data looks solid.
Yet, some key questions are left unaddressed, and some mechanistic aspects have not been explored with sufficient detail.

The manuscript lacks insight into the role of SLF2 in the DDR: the Authors show that a conspicuous number of chromatin-associated proteins is lost upon deletion of SLF2. Among these claspin, which may account for the less effective activation of CHK1. Yet there is little follow-up of this observation; thus, it remains unaddressed whether the reduction of claspin phenocopies SLF2 loss or whether there is a "pleiotropic" effect (multiple aspects of the DDR are altered). For instance, apart from the impairment of the ATR/CHK1 signaling, are SLF2 null cells defective in other processes linked to DNA damage, such as DNA repair or cell cycle checkpoint activation? Is p53 involved?

The Authors also provided omics data concerning mRNA expression and chromatin-associated proteins that show that "DNA damage" factors are altered in either expression or chromatin association. While these observations suggest a link between SLF2 loss and DNA damage/the DNA damage response, they don't explain whether such alterations are direct or indirect consequences. In addition, it is unclear whether genomic instability is a common feature observed in SLF2 silenced/ko cells or whether this is only observed in U2OS cells.

It is also unclear whether loss of SLF2 will sensitize any cell to any DNA damaging agent/DDR inhibitor. Is there any selectivity for cancer cells? Is there any selectivity in the synthetic lethality? Is there any particular reason why the authors focused on SUMOi instead of other compounds targeting other activities relevant for DNA damage signaling/repair?

Further analyses along these lines will certainly strengthen the novelty and impact of this manuscript.

Apart from the concerns expressed above, following are some detailed requests.

Major points.

1. From the abstract: "SLF2- deficiency leads to loss of DNA repair factors including CLSPN". Yet, in the manuscript the Authors show that there is less chromatin-associated CLSPN in SLF2-KO cells. Please clarify whether SLF2 controls both CLSPN expression (at what level?) and CLSPN association to chromatin.

2. The authors use DRB to induce DNA damage/breaks and analyze the DNA-damage response. Since the authors are interested in analyzing ATR/CHK1 activation, a treatment leading to preponderant single-strand breaks will be more appropriate and informative.

3. Page 4. Auth. write: "We link SLF2 deficiency to defective DNA repair ...". Please modify this since, unless it escaped my notice, in the paper, no data shows that SLF2 modulates DNA repair.

4. Loss of SLF2 in Eμ-Myc HSPCs accelerates tumor growth. Is this due to defective ATR/CHK1 signaling? Do these tumors show increased genome instability or altered DDR signaling compared to the "control" tumors? This evidence will be required to claim that loss of tumor suppression is due to reduced CHK1 activity in SLF-KO cells.

5. Fig1 shows higher gH2Ax (DNA damage) in SLF2-KO U2OS, implying that loss of SLF2 increases genome stability. Is this also seen in the other cell lines/cells in which SLF was silenced/knocked out?

6. Why does the loss of SLF2 affect the expression of DDR genes? Are these genes directly controlled by SLF2, or are these alterations a consequence of unbalances in cell cycle control/checkpoint activation? To this end, the Authors should perform the cell cycle analysis by FACS (w/ BrdU or EdU pulse labeling) upon SLF2-loss, both in unchallenged conditions and upon DRB treatment.

7. DRB triggers ATR/CHK1 activation, which is lower in cells with reduced SLF2. Is this associated with any sensitization to DRB?

8. Why is SUMOi synthetic lethal with the loss of SLF2? Is this because SUMOi is inducing the ATR/CHK1 pathway? If so, are ATR/CHK1 inhibitors SL with SUMOi as well? Please, provide a detailed analysis of the DDR and some of its key components to clarify these points.

9. Figure 4 b shows the analysis of the level of SLF2 in a panel of DLBCL lines, which show variable SLF2 expression levels. These cell lines should be tested to confirm that SLF2 low cells are sensitized to SUMOi.

Minor points.
1. Please, report MWs on all western-blot.
2. Page 3. "DNA damage activates the two key DNA damage signaling-related protein kinases ataxia telangiectasia mutated (ATM) and ATM and Rad3-related (ATR) ". Please specify the type of DNA damage that activates ATM and ATR.
3. Page 5. Concerning the incipit of the paragraph "SLF2 is a tumor suppressor of B-cell lymphomagenesis,". Please rewrite since, from the text, it is not clear that the Authors are referring to the published study cited in the figure legend.
4. What is the expression level of Slf2 in pre-tumoral and tumoral b-cells derived from Eμ-Myc mice?
5. Figure 2d. Please report the % of GFP positive cells, both at injection and in B-cells collected at the end of the experiment.
6. fig 2e. Please, indicate the total number of mice for each curve
7. Fig 3b. Please report the IC50 for the two curves

Referee #3 (Remarks for Author):

Summary
The authors present work on the SMC5-SMC6 Complex Localization Factor 2 (SLF2) to demonstrate a potential role for SLF2 in the DNA damage response (DDR). SFL2 acts as a biomarker for B-cell lymphoma (BCL) patients with poor prognosis. SLF2 deficiency leads to loss of the DDR component CLSPN, impairing CHK1 activation. Moreover, SLF2 deficiency drives lymphomagenesis in mice. Subsequently, the authors include SUMOylation in their study, although this is less well connected to the main part of the manuscript and they show synthetic lethality between SLF2 deficiency and inhibition of SUMOylation. They subsequently demonstrate synthetic lethality between CHK inhibitors and inhibition of SUMOylation, which is not well connected to SLF2, the main topic of the study. Although interesting, several points need to be addressed to strengthen the manuscript.

Major comments

1. The paper by Räschle et al. 2015 Science (reference 25 as cited in the manuscript) links SLF1 and SLF2 to RAD18 and ubiquitin signaling. Moreover, ubiquitin signaling plays a very prominent role in the DNA damage response. Therefore, SLF2 deficiency and inhibition of ubiquitin signaling could provide much stronger synergism compared to SLF2 deficiency and inhibition of SUMO signaling. I would therefore ask the authors to compare the synergy between SLF2 deficiency and either SUMO signaling or ubiquitin signaling side by side.

2. SLF1 and SLF2 are both prominent proteins in the cited paper by Räschle et al. 2015 Science. Here the authors only study SLF2 and ignore SLF1. The authors should compare SLF1 and SLF2 side by side and test whether they have overlapping or unique functions in the context of B-cell lymphomagenesis.

3. The authors claim activation of the post-translational modification SUMOylation by SLF2 deficiency. This claim is poorly substantiated. It would be important to strengthen this claim by demonstrating enhanced SUMOylation for a selected set of SUMO targets in the absence of SLF2.

4. The synergy between SLF2 deficiency and inhibition of sumoylation in figure 5 is interesting, but potential synergy with inhibition of ubiquitination could be even more interesting as mentioned in point 1.

5. The authors demonstrate synergy between SUMOi and either AZD6672 or rabusertib, which is poorly connected to SLF2 as the main topic of the study. Whereas the authors show increased polyploidy in cells that are deficient for SLF2 and are treated with SUMOi, the experiments with SUMOi and either AZD6672 or rabusertib only study apoptosis. The authors need to verify whether SUMOi and either AZD6672 or rabusertib also enhance polyploidy.

6. Subasumstat is associated with activation of the immune system (reference 38), this could be mentioned in the paper. Since Subasumstat in vivo primarily acts via the immune system, it is doubtful whether the uncovered drug synergy can be confirmed in a syngeneic mouse B-cell lymphoma model. Convincing proof that the drug synergy is also efficient in a mouse B-cell lymphoma model would strengthen the manuscript considerably.

7. The entire manuscript deals with B cell lymphoma. It would therefore be important to replace experiments that are carried out with non B cell lymphoma cells for experiments with B cell lymphoma cells.

8. For many experiments, it is unclear how many times they have been performed. Indicating reproducibility of all experiments is

therefore vital.

Minor comments:

9. The Western blots are very tightly cropped. Please crop less tightly and provide relevant size markers.

10. Adding a mechanistic cartoon in the final figure would be helpful.

Zhang et al. "Actionable loss of SLF2 drives B-cell lymphomagenesis and impairs the DNA damage response"

Point-by-point responses to the Reviewers' comments

We would like to thank the Reviewers for their valuable comments and suggestions. Accordingly, we have performed a large set of new experiments and analyses, which allowed us to substantiate the findings of our study. We are convinced that the questions raised by the reviewers and the consecutively performed experiments have substantially improved the manuscript.

Several new datasets have been included in previous figures/tables. In addition, we now provide additional data in **3 new main figures**, **11 new appendix figures,** and **2 new supplementary tables**.

Major aspects added are:

1. We performed **CRISPR/Cas9-mediated gene editing of *CLSPN*,** to generate a CLSPN-deficient cell line and to test the impact of these alterations to further explain the biology observed in SLF2-deficient lymphoma cells.

2. We performed **two additional *in vivo* experiments**, which we included in the point-by-point response to the Reviewers´ comments and to the manuscript:
   - We transplanted **primary *Eμ-Myc* lymphoma cells** into syngeneic recipient mice and treated them with SUMOi and/or rabusertib to investigate the synergism of SUMOi and rabusertib *in vivo* in an immune-competent model.
   - We generated **xenografts of the human DLBCL cell line OCI-Ly1** in NOD SCID mice and treated them with SUMOi and/or rabusertib to show the synergism of SUMOi and rabusertib *in vivo* in an immune-compromised model.

3. We performed **low-coverage whole genome sequencing** to investigate genomic instability in primary Slf2-sgRNA *Eμ-Myc* lymphomas from the in vivo validation experiments, which we provide within our response to the Reviewers´ comments.

4. We performed transcriptomic profiling by **RNA sequencing of isogenic OCI-Ly19 *SLF2^{KO}* and parental cells** to further substantiate our initial findings of an impaired DDR associated with SLF2 loss.

5. We performed transcriptomic profiling by **RNA sequencing of a doxorubicin and doxorubicin in combination with SUMOi-treated** DLBCL cell line to further substantiate the role of SUMOylation during DNA damage response.

6. We have further amended the study of the synergism of SUMOi and CHK1 and now provide data showing the synergism in **seven independent murine and human cell lines and murine primary cells**.

Please find below our point-by-point responses to the Reviewers' comments and suggestions.

Referee #1 (Remarks for Author):

Zhang and co-workers identified SLF2 as a DDR pathway regulator by modulating ATR-CLSPN-CHK1 axis, which is associated with MYC-driven B cell lymphoma. The authors elegantly showed several lines of evidence of SLF2 as a tumor suppressor in association with DLBCL. The authors claimed that a loss of SLF2 associated with a high level of DNA damage could activate sumoylation pathway as a safeguard. Therefore, they proposed co-targeting of the sumoylation and DDR pathways is a promising strategy for the treatment of aggressive lymphoma. Several reports have demonstrated sumoylation pathway is a synthetic lethal partner for oncogenes such as MYC and RAS. Previous studies have also shown SLF2 as a player of the DDR. Therefore, co-targeting of the DDR and sumoylation for the treatment of MYC-driven lymphoma is not conceptually advanced. The main novelty of this study is the identification of SLF2 as a biomarker for B-cell lymphoma and SLF2 in regulating the DDR pathway via CLSPN. While the current format of this manuscript showed the biological significance of SLF2 in the DDR pathway, the mechanistic insight as to how SLF2 modulates CLSPN levels and how SLF2 deficiency leads to an aberrant sumoylation signature (globally or mainly the key factors associated with DDR pathway) should be addressed to increase the cohesiveness and novelty of this study. Several suggestions to improve the quality of this manuscript are as follows:

We thank Reviewer #1 for the positive reception of our manuscript and appreciation of the study's novelty regarding the identification of SLF2 as a biomarker for B-cell lymphoma and SLF2 in regulating the DDR pathway via CLSPN.

Major

1. The current data provided association of SLF2 in DNA repair, MYC-driven B cell lymphomagenesis, and requirement for ATR-CLSPN-CHK1 axis. It is not clear how SLF2 alters CLSPN levels. The mechanistic study should be included to enhance the novelty of this study.

We thank Reviewer #1 for bringing this important point to our attention. We have performed a set of experiments to address this question. Of note, neither proteasome inhibition (MG132) nor inhibition of ubiquitination (Ub E1 inhibitor) could rescue the depletion of CLSPN in SLF2-deficient SU-DHL-5 cells. In addition, we investigated the effect of SLF2 on the protein half-life of CLPSN by global inhibition of protein biosynthesis using cycloheximide. We treated SU-DHL-5 parental and SLF2-deficient cells with 20 µg/ml of cycloheximide for 2 and 4 hours and quantified the western blots to calculate the half-life of SLF2 in these two cell lines. There was no appreciable effect here either and we therefore exclude an effect at the translational level while suspecting regulation of CLSPN at the transcriptional level. Of note, we observed significantly lower CLSPN mRNA levels in SLF2-deficient cells, revealing transcriptional regulation of CLSPN. We included these data as Figure 5D and Appendix Figure S9.

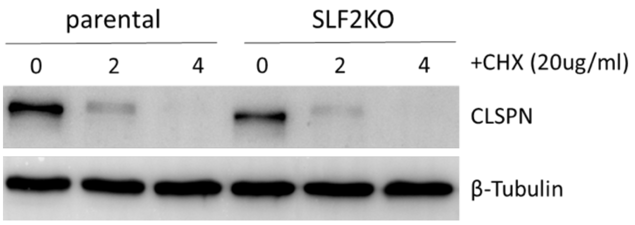

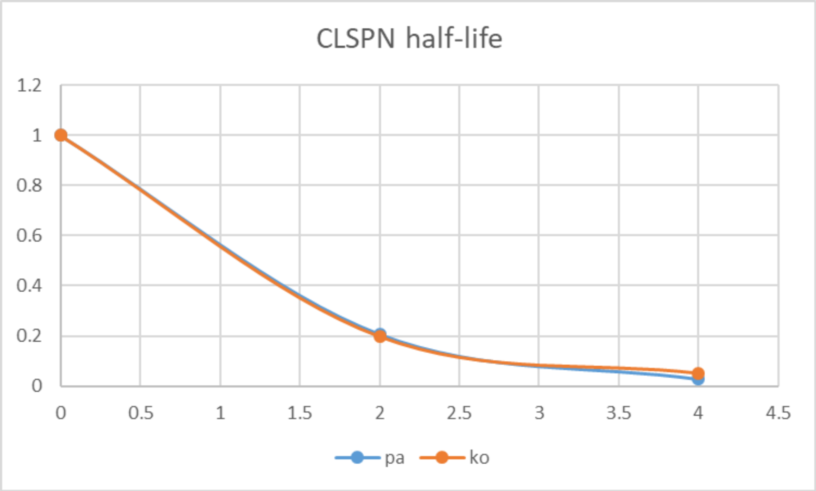

2. The authors claimed that NSMCE2 is a SUMO E3 ligase able to form complexes with SLF2 and NSMCE2 deficient cells also showed impairment of CHK1 activation. Does NSCMCE2 also affect CLSPN levels? Is SLF2 deficiency-elicited any sumoylation change due to NSMCE2? Likewise, does NSMCE2 deficiency alter sumoylation associated with the DDR pathway? Since NSMCE2 could suppress cancer independently of its SUMO ligase activity (EMBO J 2015, 34(21):2604-2619), it should be clarified the issue of NSMCE2 in sumoylation regulation and the interplay among SLF2, NSCMCE2, and sumoylation change.

We thank Reviewer #1 for raising this point. We would like to underscore that we did not focus our mechanistic studies on NSCME2. We have performed substantial unbiased mass-spec-based experiments to characterize the proteome of cells in the context of SLF2 loss and, moreover, specifically of DNA-bound proteins following SLF2 loss (Figure 5A and Appendix Figure S7). Based on this unbiased experimental approach, we have identified a set of proteins that could explain the biology we observed in SLF2-deficient cells and validated them (Figure 5). Out of these validated candidate proteins, we have further studied CLSPN, which is a direct regulator of CHK1 activation. We have now also generated CLSPN-deficient cells lines by CRISPR/Cas9 based gene editing (Figure 7A). Of note, CLSPN loss lead to similar effects like we observed following SLF2 loss. Moreover, we have studied the level of CLSPN regulation following SLF2 loss (Figure 5B-D, F and Appendix Figure S7).

We specifically included the NSMCE2-related data in appreciation of the literature, which has described NSMCE2 as a SUMO E3 ligase forming complexes with SLF2 via the SMC5/6 complex[1]. Therefore, we tested the relevance of this axis for B-cell lymphoma biology. We included the respective reference and additional data in our manuscript and Appendix Figure S12. NSCME2 loss caused similar effects on CHK1 activation, sensitivity to SUMOi and CLSPN levels as SLF2 loss (Appendix Figure S12), however, we could not observe any effect on NSCME2 or SMC5 expression following SLF2 loss (New Figure 5F and Table EV2, 4). Based on this we concluded that the effect of SLF2 loss could not be explained via impaired NSMCE2. This has been clarified in the manuscript.

An even more detailed analysis of the NSMCE2-SLF2 axis would require complex and further extensive analyses which is clearly beyond the scope of this manuscript on the biology of SLF2 in the lymphoma context.

Whether such sumoylation alteration is associated with SLF2 deficiency-elicited aberrant sumoylation signature should be addressed.

We thank Reviewer #1 for raising this important question. Importantly, the SUMOylation pathway is regulated on various layers from SUMO conjugation controlled by E1 (SAE1/SAE2), E2 (UBE2I) and E3 ligases to de-SUMOylation, which is tightly controlled by

SUMO isopeptidases. Therefore, high SUMO pathway activity does not automatically reflect in a high level of SUMOylated proteins as this pathway is heavily counteracted by SUMO isopeptidases. The SUMO pathway inhibitor TAK-981 directly targets the E1 complex. To directly link the activity of this part of the SUMO pathway with SLF2 loss, we investigated the expression levels of SAE1 and SAE2 in SLF2-deficient DLBCL cells. We found significantly higher SAE1 expression in SLF2-deficient DLBCL cells, thus directly linking SLF2 loss to SUMO conjugation pathway activation. Beyond that, we also observed significant activation of SAE1 expression in CLSPN-deficient DLBCL cell lines, again similar to the effects of SLF2 loss. We included the data in Figures 6C, D and Figures 7D, E.

Minor

1. Dysregulated genes involved in lymphomas and wild-type B cells should be presented by heatmap in Fig. 1a and 1b.

According to the Reviewer's suggestion we have included heatmaps depicting all dysregulated genes corresponding to the running sum plots of Figures 1A and 1B as Appendix Figures S1 and S2.

2. The biological effect of the other candidates (Fig. 1e) should be demonstrated as Fig 1h.

Using an unbiased screen, we were able to identify several interesting and little-studied tumor suppressor candidate genes. By further filtering, we were able to decimate these candidates to six and subsequently focused our work on SLF2 for various reasons already outlined in the manuscript. We agree with the reviewer that the other five candidates are also highly interesting and worthy of further analysis. We therefore further investigated the relevance of these candidates in the context of DLBCL. In particular, we were able to associate CNOT2 and NUCKS1 mRNA expression with adverse survival. These data are now presented in the new Appendix Figure S4.

3. In Fig. 1h, the DAPI images in control set need to be fixed (upside down).

We thank the Reviewer for pointing us toward this important issue. We fixed the DAPI images in the control set accordingly.

4. In Fig. 4d, the authors claimed that CHK2 activation was not altered by SLF2 depletion. However, the Western blot showed a slight reduction of phospho-CHK2 in SLF-KO cells. Quantification of these band intensities may help to resolve this issue.

We quantified p-CHK2 relative to CHK2 and relative to β-Actin. Despite observing a trend, we did not observe a significant reduction of CHK2 activation. We included the data in Appendix Figure S5.

5. Why SLF2 exhibited two bands in Western blots?

We interpret the second band as an unspecific band. Following additional optimization of the immunoblotting protocol, we could not observe these bands anymore. Importantly, we validated the antibody-based on gene-editing (SLF2 knockout) and ectopic expression of SLF2.

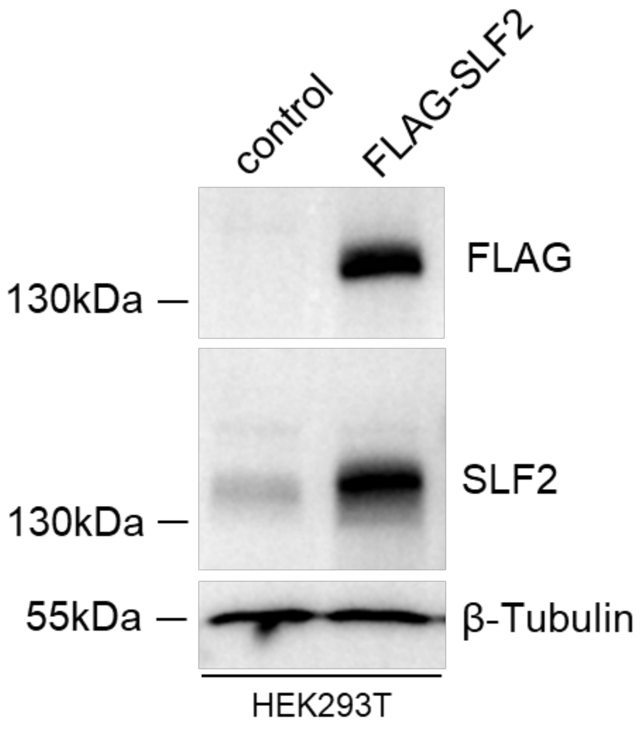

6. What is the correlation between SLF2 levels, cell proliferation / DNA damage foci number, and CLSPN levels among the patient-derived DLBCL cell lines (Figure 4b)?

Considering the substantial genetic and non-genetic heterogeneity of patient-derived human cancer cell lines, we entirely rely on cell lines with genetically altered SLF2 status. To address this question, we performed transcriptomic profiling of isogeneic SU-DHL-5 SLF2- deficient and -proficient cells. Beyond that, we generated an additional isogeneic cell line with defined SLF2 status (OCI-Ly19 *SLF2*^{KO}) and again performed transcriptome profiling. In both experiments, we observed significant alterations in the cell cycle and DNA repair pathways. This finding was further corroborated by a BrdU cell cycle analysis of SU-DHL-5 control and *SLF2*^{KO} cells, which showed significant proliferation defects in SLF2-deficient cells. We included these data in Table EV1 and Appendix Figure S8 and S11A.

7. Fig. 4f: this is not a quantification mass result.

We revised the figure legends accordingly to "~~*Comparative*~~ *TMT-based MS results of chromatin fractions from SU-DHL-5 control and SU-DHL-5 SLF2*^{KO} *cells. Volcano plot depicting depleted chromatin-associated proteins. Significant hits are shown by red dots (depleted in SLF2*^{KO} *cells).*"

8. In Fig. 5d-i, the authors didn't indicate the specific concentration of inhibitors.

We revised the manuscript accordingly.

9. SU-DHL-5 control and SLF2 KO cells should be included in Fig 5h.

We thank the Reviewer for raising this question. We generally propose that compromised DNA damage response acts synergistically with the inhibition of SUMOylation. This compromised DNA damage response, specifically compromised CHK1 activation can be either

caused by loss of SLF2 or pharmacological inhibition of CHK1 (rabusertib). We, therefore, did not include the SU-DHL-5 control and *SLF2*^{KO} cells in the figure highlighting the synergism for SUMOi and pharmacological inhibition of CHK1 (new Figure 8). To address the Reviewer´s question, we included the data in the Reviewer´s only section. While SUMOi and rabusertib generally drive synergistic cell death in SU-DHL-5 cells (left panel), we did not observe significantly more cell death in SU-DHL-5 *SLF2*^{KO} cells treated with the combination when compared to SUMOi alone (middle and right panel). Please note that we here show data with a low SUMOi concentration, which did just start to induce cell death in the *SLF2*^{KO} cells, but not in the less sensitive control cells. This concentration showed the biggest trend in the difference between SUMOi and SUMOi + rabusertib treatment, however, still no significant difference (*P-value* determined with ANOVA, Tukey`s post hoc test).

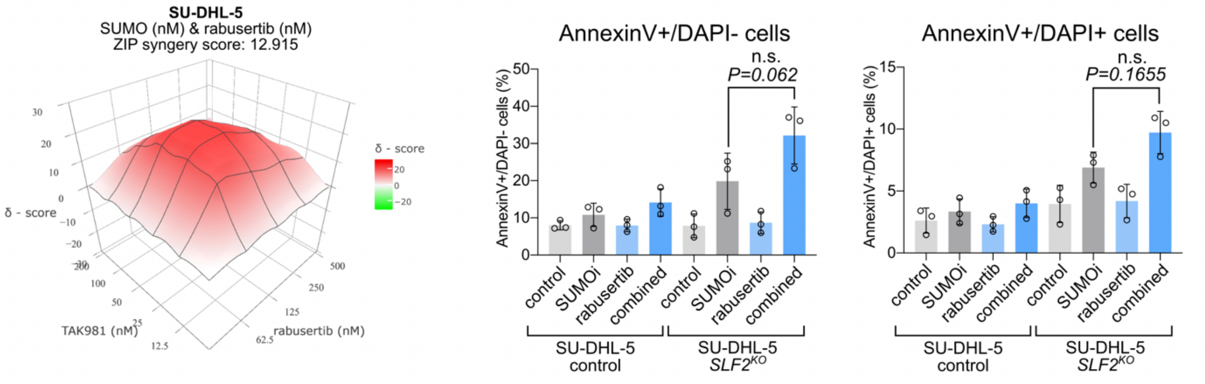

10. You should define this abbreviation the first time and then only use the abbreviated. For instance, the abbreviation "DLBCL" was used in the first paragraph of the Introduction without definition.

We revised the manuscript accordingly.

Referee #2 (Remarks for Author):

In this manuscript, Schick and colleagues identify SLF2 as a tumor suppressor gene in Eμ-myc lymphomas. They also show that SLF2 is suppressed and associated with a worse prognosis in human DLBCL. Given previous reports linking SLF2 to the DNA damage response (DDR), the Authors investigated whether loss of SLF2 leads to (i) increased genome instability and (ii) alterations in DDR signaling. Among the most salient findings, they show that loss of SLF2 impairs CHK1 activation. They also show that loss of SLF2 sensitizes cells to an inhibitor of sumoylation.

We thank Reviewer #2 for the positive reception of our manuscript and for highlighting the novelty of our work.

The manuscript has the potential merit of having identified a new tumor suppressor gene in DLBCLs (and possibly other non-Hodgkin lymphomas), provides clues concerning SLF2 molecular function/mechanism, and shows evidence for the selective preclinical targeting of lymphomas characterized by low expression of SLF2. For the most part, experiments are well structured and executed, and the data looks solid. Yet, some key questions are left unaddressed, and some mechanistic aspects have not been explored with sufficient detail. The manuscript lacks insight into the role of SLF2 in the DDR: the Authors show that a conspicuous number of chromatin-associated proteins is lost upon deletion of SLF2. Among these claspin, which may account for the less effective activation of CHK1. Yet there is little

follow-up of this observation; thus, it remains unaddressed whether the reduction of claspin phenocopies SLF2 loss or whether there is a "pleiotropic" effect (multiple aspects of the DDR are altered). For instance, apart from the impairment of the ATR/CHK1 signaling, are SLF2 null cells defective in other processes linked to DNA damage, such as DNA repair or cell cycle checkpoint activation? Is p53 involved?

We thank Reviewer #2 for raising these important points. To address these, we have generated a CLSPN-deficient lymphoma cell line by CRIPSR/Cas9 (SU-DHL-5 *CLSPN*^{KO}). As expected, CLSPN-deficient cells showed compromised CHK1 phosphorylation. Beyond this, similar to the effects of SLF2 loss, CLSPN loss conferred synthetic lethality to SUMOi and less sensitivity to mafosphamide. Based on this, we conclude that CLSPN is sufficient to mediate similar effects like SLF2 loss. We included the data in new Figure 7.

We also investigated the potential involvement of p53 in mediating the effects of SLF2 loss. First, we triggered the p53 axis in SU-DHL-5 control and *SLF2*^{KO} cells and analyzed p53 and p-p53 expression. There was no significant effect on p53 phosphorylation/activation in SLF2-deficient DLBCL cells. However, we found a trend towards impaired p53 activation and thus generated shRNA-mediated p53 knockdowns to investigate if loss of p53 could explain the biology we observed following SLF2 loss.

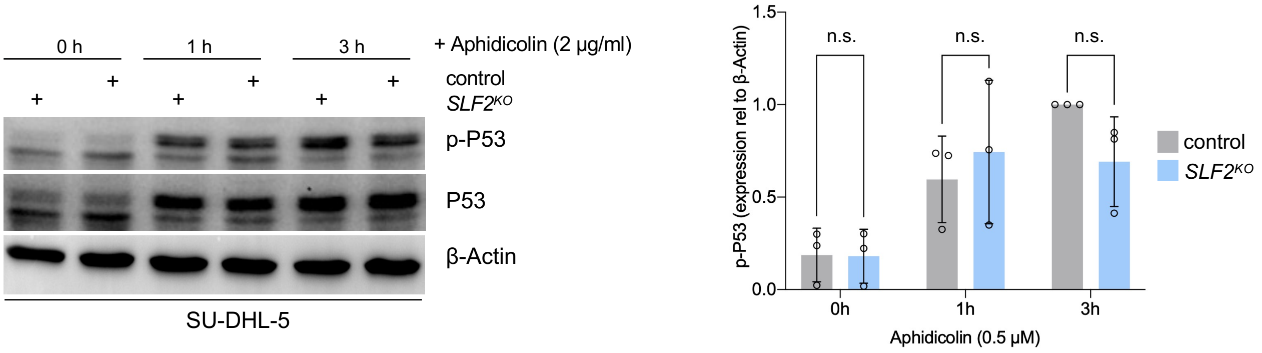

Both shRNAs targeting TP53 lead to substantial depletion of p-P53. We then treated the control and p53-deficient cells with increasing concentrations of SUMOi to derive dose-response curves. Of note, while SLF2 loss conferred synthetic lethality to SUMOi, we observed reduced sensitivity to SUMOi in p53-deficient cells. In summary, based on these experiments, we could exclude that p53 plays a role in mediating the effects of SLF2 loss.

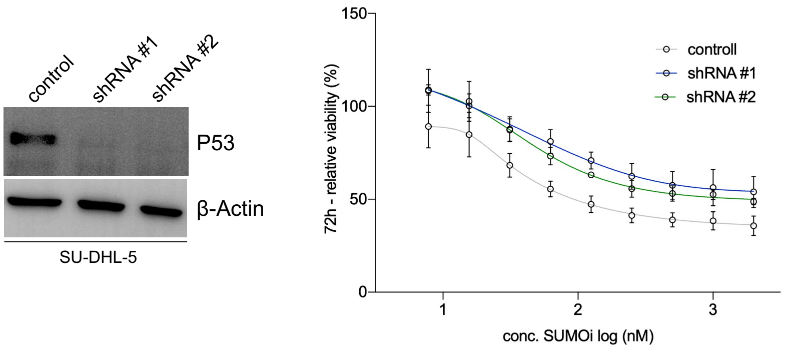

Moreover, we performed an experiment to characterize the cell cycle of an SLF2-deficient and proficient DLBCL cell line without and with DNA damage stimulus, which we describe below in our response to major point 6 (Reviewer #2).

The Authors also provided omics data concerning mRNA expression and chromatin-associated proteins that show that "DNA damage" factors are altered in either expression or chromatin association. While these observations suggest a link between SLF2 loss and DNA damage/the DNA damage response, they don't explain whether such alterations are direct or indirect consequences.

We address this question in our response to major point 6 (Reviewer #2).

In addition, it is unclear whether genomic instability is a common feature observed in SLF2 silenced/ko cells or whether this is only observed in U2OS cells.

We address this question in our response to major point 5 (Reviewer #2).

It is also unclear whether loss of SLF2 will sensitize any cell to any DNA damaging agent/DDR inhibitor. Is there any selectivity for cancer cells? Is there any selectivity in the synthetic lethality? Is there any particular reason why the authors focused on SUMOi instead of other compounds targeting other activities relevant for DNA damage signaling/repair? Further analyses along these lines will certainly strengthen the novelty and impact of this manuscript.

We thank Reviewer #2 for raising these important questions. Based on the Reviewer´s comments, we have performed a set of additional analyses and experiments to explain how we identified this actionable molecular vulnerability defined by SLF2 loss.

We now included a pathway analysis we have performed based on the unbiased mass-spec-based characterization of the chromatin proteome of SLF2-deficient DLBCL cells. In this pathway analysis, we identified the pathway *"SUMOylation of DNA replication proteins"*, which prompted us to further investigate this pathway in the context of the SLF2 status. From there on we investigated the SUMO conjugation pathway, specifically one of the rate-limiting factors, the E1 ligase complex SAE1/SAE2 in control and SLF2-deficient DLBCL cells. We found significantly higher SAE1 expression in SLF2-deficient lymphoma cells. Of note, we found a similar increase in SAE1 expression in CLSPN-deficient lymphoma cells, which prompted us to test the SUMOylation inhibitor in the context of SLF2 deficiency. We included these data in Figures 6C, 6D, 7D, 7E and Table EV3.

Moreover, we wanted to highlight that the literature was heavily investigating and describing the mechanistic link of SUMOylation and DNA repair/DNA damage response over the last years, e.g. discussed in "Principles of ubiquitin and SUMO modifications in DNA repair"[2], which we also have included and discuss in our manuscript.

To test if SLF2 depletion also sensitizes non-cancer cells, we used a siRNA to deplete SLF2 in human HEK293T cells and murine NIH3T3 cells. Despite these cell lines being immortalized, they do not originate from cancer. Importantly, in neither of the two cell lines, SLF2 loss sensitized to SUMOi treatment and we concluded that the effect is selective for cancer cells. We included the data in Appendix Figure S10.

To test if SLF2 loss would sensitize to other DNA damaging agents, we also treated control and SLF2-deficient DLBLC cells with doxorubicin (response to major point 7), mafosphamide (Figure 3E), hydroxyurea and cisplatin. SLF2-deficient cells were not sensitized against any of the four tested DNA-damaging agents but showed increased resistance against mafosphamide and cisplatin.

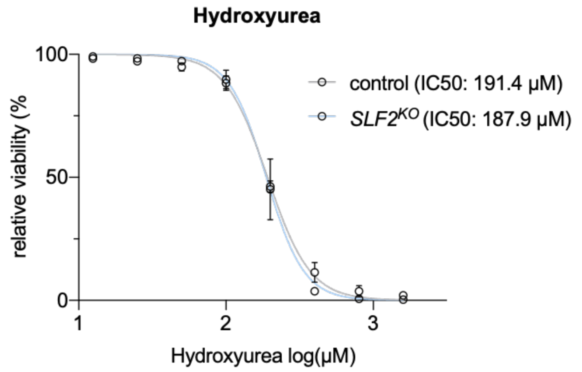

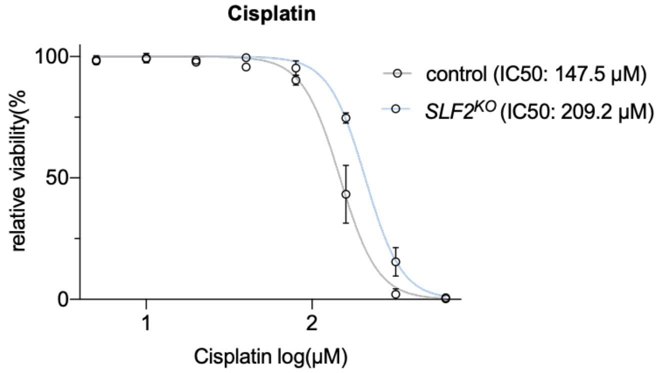

Moreover, we also tested the DDR inhibitors Olaparib and AZD6738 targeting PARP and ATR respectively. SLF2-deficient lymphoma cells were not sensitized toward either of these inhibitors.

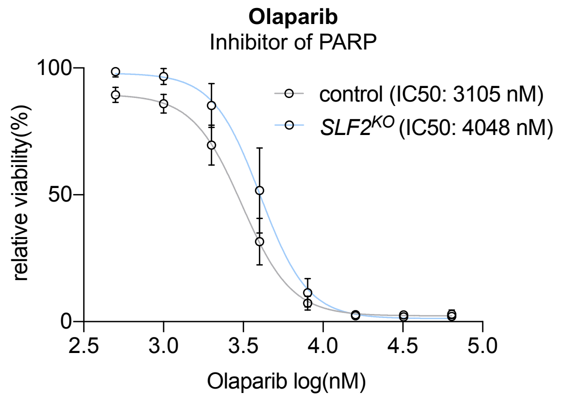

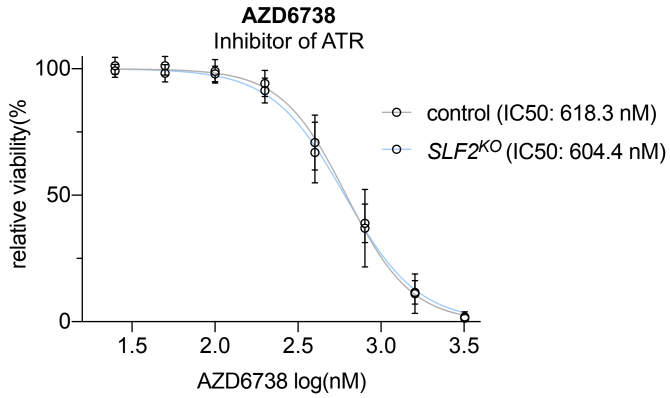

Beyond that, we also tested the impact of inhibiting the Ubiquitin proteoasome system and treated SLF2-deficient cells with bortezomib (proteasome inhibitor) and TAK-243 (Ub E1 inhibitor; also discussed in our answer to major point 1, Reviewer #3). SLF2-deficient cells were not sensitized toward either of the inhibitors.

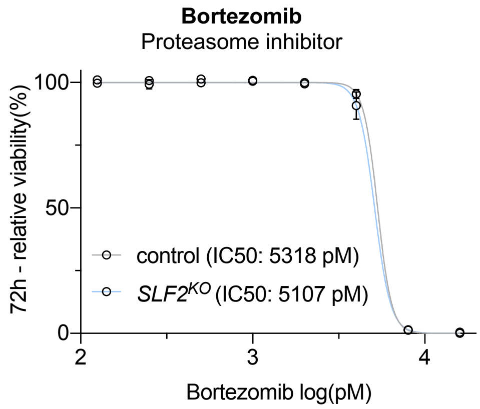

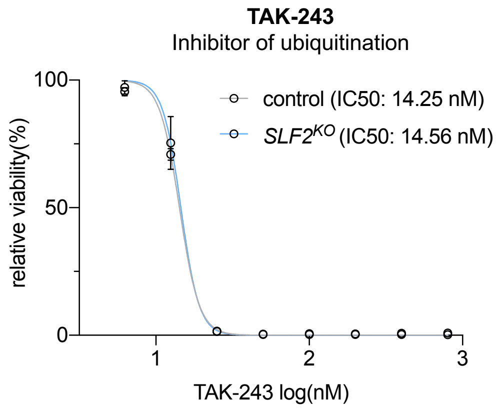

Based on these additionally performed experiments, we conclude that SLF2 loss does not sensitize cancer cells to any type of DNA damage or any DDR inhibitor.

Apart from the concerns expressed above, following are some detailed requests.

Major points.

1. From the abstract: "SLF2- deficiency leads to loss of DNA repair factors including CLSPN". Yet, in the manuscript the Authors show that there is less chromatin-associated CLSPN in SLF2-KO cells. Please clarify whether SLF2 controls both CLSPN expression (at what level?) and CLSPN association to chromatin.

We thank the Reviewer for pointing us to this. We observed depletion of CLSPN in both whole cell lysates and chromatin fractions. Based on this we conclude that SLF2 controls overall CLSPN expression, leading to reduced CLSPN levels at chromatin (Figure 5A, F and Appendix Figure S7). This conclusion is further strengthened by the suppression of CLSPN mRNA levels in SLF2-deficient lymphoma cells (Figure 5D). Please see also our response to the related questions raised by Reviewer 1.

2. The authors use DRB to induce DNA damage/breaks and analyze the DNA-damage response. Since the authors are interested in analyzing ATR/CHK1 activation, a treatment leading to preponderant single-strand breaks will be more appropriate and informative.

We thank Reviewer #2 for raising this important point. We have amended our experiments and now also used hydroxyurea (HU) and aphidicolin to promote CHK1 activation. HU treatment causesa lack of deoxynucleotide triphosphates (dNTPs) and consequent stalling of replication forks[3,4]. At the stalled replication fork the single-stranded DNA generates a signal that activates the ATR checkpoint and prevents the cell cycle from transitioning from S into G2 phase until replication is completed[5]. Similarly, aphidicolin blocks dNTP binding to DNA polymerase alpha, generating stalled replication forks, and inducing single-strand DNA damage signaling[6,7]. With both stimuli, we observed similar defects in CHK1 activation as we observed with DRB. We included the data in Appendix Figure S6.

3. Page 4. Auth. write: "We link SLF2 deficiency to defective DNA repair ...". Please modify this since, unless it escaped my notice, in the paper, no data shows that SLF2 modulates DNA repair.

We revised the manuscript accordingly and thank Reviewer #2 for raising this important point. To further investigate the DDR and potentially DNA repair, we performed a time-course experiment. To this end, we treated SU-DHL-5 control and *SLF2*^{KO} cells for 30 minutes with DRB, washed out DRB and cultured the cells for defined timepoints before harvest for immunoblot analysis. We found a substantially higher level of phospho-DNA-PKcs indicating the presence of a high level of DNA damage in SLF2-deficient lymphoma cells, which is further underscored by the high levels of phopho-RPA32 and γ-H2A.X we observed. We included the data in Appendix Figure S5F. We agree that the statement should be revised and changed it accordingly in the manuscript.

4. Loss of SLF2 in Eμ-Myc HSPCs accelerates tumor growth. Is this due to defective ATR/CHK1 signaling? Do these tumors show increased genome instability or altered DDR signaling compared to the "control" tumors? This evidence will be required to claim that loss of tumor suppression is due to reduced CHK1 activity in SLF-KO cells.

We thank the Reviewer for raising this important question.

To investigate the impact of SLF2 loss on CHK1 signaling in murine *Eμ-Myc* lymphomas, we depleted SLF2 with an shRNA specific for *Slf2* in a cell line derived from a primary *Eμ-Myc* lymphoma. This allowed us to generate larger cell numbers and perform a set of short-term treatment experiments, which would have been challenging with the primary lymphoma cell

cultures we used for the transcriptome profiling experiment (only short-term culture, transduction and then RNA isolation feasible). SLF2 expression was significantly lower in lymphoma cells transduced with the *Slf2* shRNA and SLF2 loss led to compromised CHK1 activation in this *Eμ-Myc* lymphoma cell line. From this we conclude, that SLF2 loss leads to compromised CHK1 activation in SLF2-deficient *Eμ-Myc* lymphomas. The data are shown in Figure 4G-I.

Beyond this and to further investigate genome stability in primary *Eμ-Myc* lymphomas from the *in vivo* validation experiments, we isolated DNA from snap frozen tissue and performed low-coverage whole genome sequencing as described in Lange et al.[7] This approach allowed us to specifically determine copy number alterations in these primary control and Slf2-sgRNA lymphomas. We did not observe a significantly different frequency of copy number alterations in control and SLF2-deficient murine lymphomas.

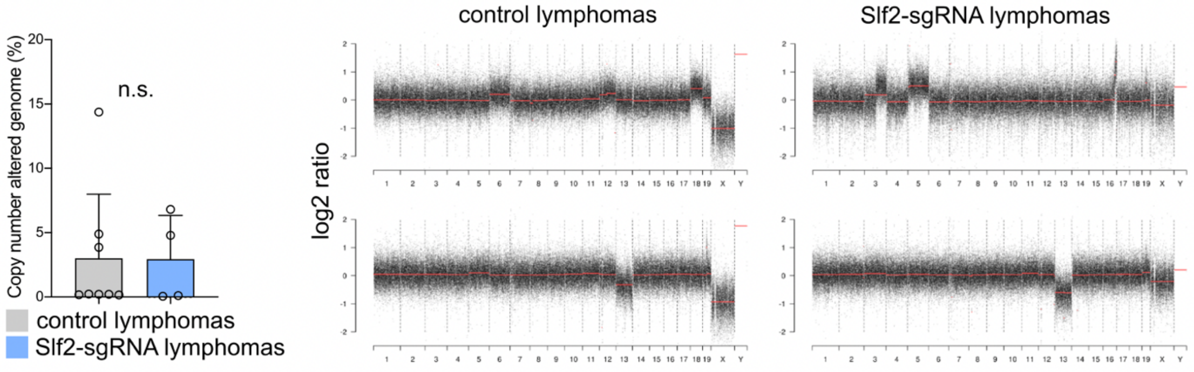

This observation prompted us to investigate genome stability in human aggressive B-cell lymphoma patients. To this end, we investigated the association of *SLF2* mRNA expression with the number of copy number alterations and mutations in the publicly available TCGA DLBCL cohort (analyzed with https://www.cbioportal.org). Of note, while we did not observe an association of *SLF2* expression with copy number alterations (in line with our analysis of murine lymphomas), we found a significant inverse association of *SLF2* expression with the number of mutations, thus linking a low level of *SLF2* expression with a higher number of mutations and genomic instability in aggressive human DLBCL. We included the correlation of mutation count and SLF2 mRNA expression in Appendix Figure S5G.

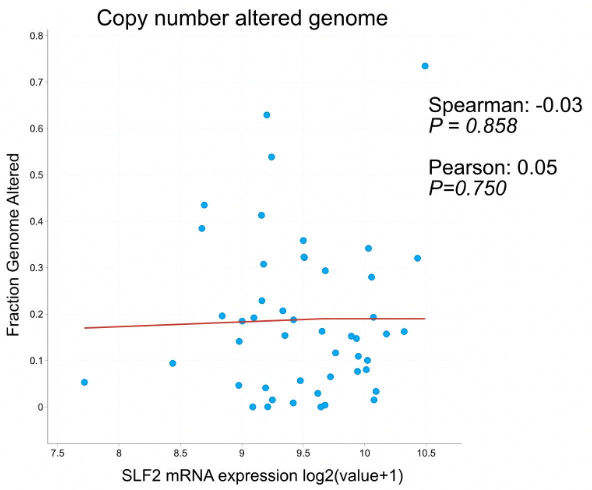

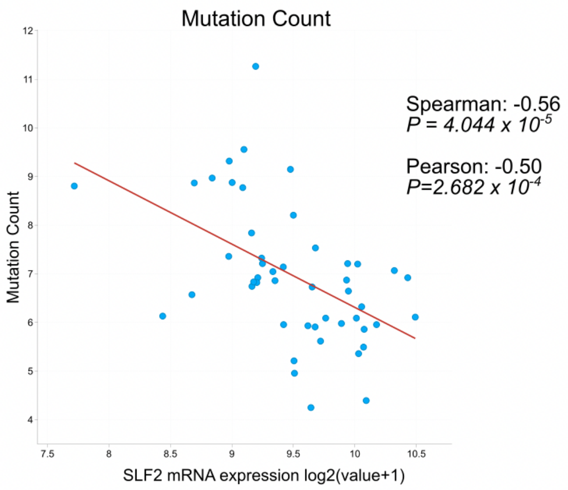

Moreover, we would like to highlight that the finding that regulators of the CHK1 axis often act as tumor suppressors is in line with previous studies described in the literature[8], and CHK1 itself has been described as having properties of a tumor suppressor, e.g. accelerated tumor development upon heterozygous deletion of CHK1[9].

5. Fig1 shows higher gH2Ax (DNA damage) in SLF2-KO U2OS, implying that loss of SLF2 increases genome stability. Is this also seen in the other cell lines/cells in which SLF was silenced/knocked out?

We thank Reviewer #2 for raising this question. We also observed a higher level of γ-H2A.X in SLF2-deficient lymphoma cells (SU-DHL-5 *SLF2*^{KO}). We included the data in Appendix Figure S5F.

6. Why does the loss of SLF2 affect the expression of DDR genes? Are these genes directly controlled by SLF2, or are these alterations a consequence of unbalances in cell cycle control/checkpoint activation? To this end, the Authors should perform the cell cycle analysis by FACS (w/ BrdU or EdU pulse labeling) upon SLF2-loss, both in unchallenged conditions and upon DRB treatment.

We thank Reviewer #2 for this important suggestion. To address this point, we performed cell cycle analysis by FACS in SU-DHL-5 control and *SLF2*^{KO} cells in unchallenged and DRB-challenged conditions. In unchallenged conditions, we observed a significantly higher fraction of cells in G0/G1 phase, while we observed less cells in S and G2M phases. Notably, while a significantly higher fraction of SU-DHL-5 control cells were arrested in G2M upon DRB challenge, *SLF2*^{KO} failed to arrest in G2M phase, which is in line with the defective CHK1 axis we describe in SLF2 deficient cells. We conclude that the alterations in transcriptome profiles are mostly a indirect effect of altered cell cycle (reflected in altered cell cycle pathways, Table EV1) and an indirect effect of DDR signaling. We included this data in Appendix Figure S8.

7. DRB triggers ATR/CHK1 activation, which is lower in cells with reduced SLF2. Is this associated with any sensitization to DRB?

We thank Reviewer #2 for raising this important point. We treated SU-DHL-5 control and *SLF2*^{KO} cells with increasing concentrations of DRB to generate dose-response curves. We did not observe any sensitization of SLF2-deficient cells to DRB.

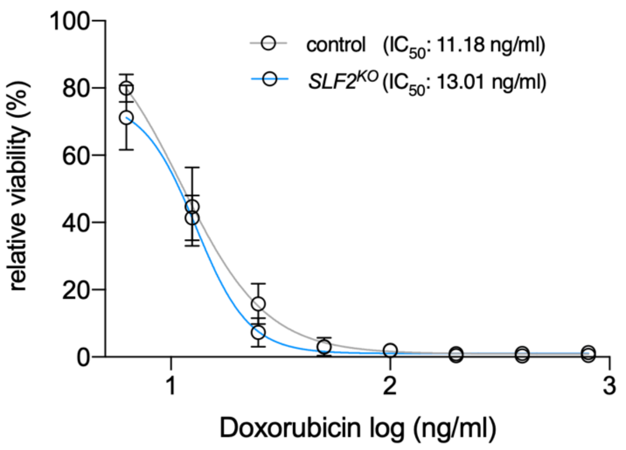

8. Why is SUMOi synthetic lethal with the loss of SLF2? Is this because SUMOi is inducing the ATR/CHK1 pathway? If so, are ATR/CHK1 inhibitors SL with SUMOi as well? Please, provide a detailed analysis of the DDR and some of its key components to clarify these points.

We thank Reviewer #2 for suggesting these important experiments. To address this point, we have pre-treated a DLBCL cell line with SUMOi and subsequently with DRB for 3 h to promote activation of the CHK1 axis. Of note, SUMOi led to compromised CHK1 activation and we conclude that SUMOi is exploiting the reduced capability for CHK1 activation in SLF2-deficient lymphoma cell. We included the data in Appendix Figure S15.

We have also combined SUMOi with CHK1 inhibitor. Indeed the two inhibitors acted highly synergistic and we could establish this synergism in seven different cell lines across species and tumor types, revealing a highly conserved vulnerability. The data is depicted in Figure 8 and Appendix Figure S13. Moreover, the combination of SUMOi and CHK1i induced a similar polyploidy effect as we observed for SUMOi in SLF2-deficient lymphoma cells. We included this data in Figure 8D and Appendix Figure S14.

9. Figure 4b shows the analysis of the level of SLF2 in a panel of DLBCL lines, which show variable SLF2 expression levels. These cell lines should be tested to confirm that SLF2 low cells are sensitized to SUMOi.

We thank Reviewer #2 for raising this important point. Considering the substantial genetic and non-genetic heterogeneity, we did, on purpose, not compare the different DLBCL cell lines among each other. Here, we are convinced that one entirely relies on cell lines with genetically edited and defined SLF2 status, thus resembling a pair of control and *SLF2*^{KO} cell lines solely differing in this one factor. To strengthen our claim, we have generated an additional SLF2 knockout cell line (OCI-Ly19). Of note, *SLF2*^{KO} cells were again more sensitive to SUMOi treatment than the control cell lines. Beyond that, we performed global transcriptome profiling and could again link SLF2 deficiency to defective DDR. We included this data in Appendix Figure S11 A, B and Table EV1.

Minor points.
1. Please, report MWs on all western-blot.

We included all MWs on western bots in the manuscript and provide all full and uncropped scans of membranes including MWs as additional information.

2. Page 3. "DNA damage activates the two key DNA damage signaling-related protein kinases ataxia telangiectasia mutated (ATM) and ATM and Rad3-related (ATR) ". Please specify the type of DNA damage that activates ATM and ATR.

We specified the type of DNA damage that activates ATM and ATR and included this sentence to the introduction: *"While ATM primarily senses double strand breaks (DSBs), ATR primarily senses single strand DNA (ssDNA)."*

3. Page 5. Concerning the incipit of the paragraph "SLF2 is a tumor suppressor of B-cell lymphomagenesis,". Please rewrite since, from the text, it is not clear that the Authors are referring to the published study cited in the figure legend.

We thank Reviewer #2 for pointing towards this. With the paragraph *"SLF2 is a tumor suppressor of B-cell lymphomagenesis"*, we are indeed not referring to the published work cited. While the cited work has found *Slf2* in a global *in vivo* screening as a candidate tumor suppressor gene (among with 957 other genes; Supplementary table in the cited work), we have performed in vivo CRISPR/Cas9 based gene editing to genetically prove its function as a tumor suppressor gene B-cell lymphomagenesis (Figure 2E). This strong and novel genetic evidence is not part of any published work.

4. What is the expression level of Slf2 in pre-tumoral and tumoral b-cells derived from Eμ-Myc mice?

To address this question, we analyzed *Slf2* mRNA levels by qPCR in pre-tumoral *Eµ-Myc* B-cells (mice sacrificed at 4 weeks of age; CD19-positive B-cells purified) and Eµ-Myc lymphomas. *Slf2* mRNA expression was significantly lower in *Eµ-Myc* lymphomas,
(Reviewers only)

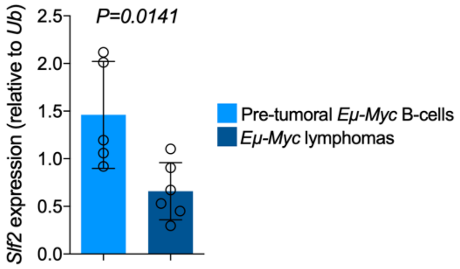

5. Figure 2d. Please report the % of GFP positive cells, both at injection and in B-cells collected at the end of the experiment.

We included a figure depicting the % of GFP positive cells, both at injection and in B-cells collected at the end of the experiment in Appendix Figure S3A.

6. fig 2e. Please, indicate the total number of mice for each curve.

We thank the Reviewer for raising this issue. We now indicate the total number of mice for each curve in the Figure legend of Figure 2.

7. Fig 3b. Please report the IC50 for the two curves.

We now report the IC50 values for all dose-response curves throughout the manuscript.

Referee #3 (Remarks for Author):

Summary
The authors present work on the SMC5-SMC6 Complex Localization Factor 2 (SLF2) to demonstrate a potential role for SLF2 in the DNA damage response (DDR). SFL2 acts as a biomarker for B-cell lymphoma (BCL) patients with poor prognosis. SLF2 deficiency leads to loss of the DDR component CLSPN, impairing CHK1 activation. Moreover, SLF2 deficiency drives lymphomagenesis in mice. Subsequently, the authors include SUMOylation in their study, although this is less well connected to the main part of the manuscript and they show synthetic lethality between SLF2 deficiency and inhibition of SUMOylation. They subsequently demonstrate synthetic lethality between CHK inhibitors and inhibition of SUMOylation, which is not well connected to SLF2, the main topic of the study. Although interesting, several points need to be addressed to strengthen the manuscript.

We thank Reviewer #3 for her/his review of our study and the positive reception of our manuscript.

Based on the Reviewer´s comments, we have performed a set of additional analyses and experiments to explain how we identified SUMOylation as an actionable molecular vulnerability following SLF2 loss.

We now included a pathway analysis we had performed based on the unbiased mass-spec-based characterization of the chromatin proteome of SLF2-deficient DLBCL cells. In this pathway analysis, we identified the pathway *“SUMOylation of DNA replication proteins”*,

which prompted us to further investigate this pathway in the context of the SLF2 status. From there on we investigated the SUMO conjugation pathway, specifically one of the rate-limiting factors, the E1 ligase complex SAE1/SAE2 in control and SLF2-deficient DLBCL cells. We found significantly higher SAE1 expression in SLF2-deficient lymphoma cells. Of note, we found a similar increase in SAE1 expression in CLSPN-deficient lymphoma cells, which prompted us to test the SUMOylation inhibitor in the context of SLF2 deficiency. We included these data in Figures 6C, 6D, 7D, 7E and Table EV3.

Major comments

1. The paper by Räschle et al. 2015 Science (reference 25 as cited in the manuscript) links SLF1 and SLF2 to RAD18 and ubiquitin signaling. Moreover, ubiquitin signaling plays a very prominent role in the DNA damage response. Therefore, SLF2 deficiency and inhibition of ubiquitin signaling could provide much stronger synergism compared to SLF2 deficiency and inhibition of SUMO signaling. I would therefore ask the authors to compare the synergy between SLF2 deficiency and either SUMO signaling or ubiquitin signaling side by side.

We thank the Reviewer for raising this important question. To address this point, we treated SU-DHL-5 control and *SLF2*^{KO} cell lines with TAK-243, a potent small-molecule inhibitor of the ubiquitin-activating enzyme (UAE), and assessed the viability following 72 h of TAK-243 treatment via flow cytometry. We did not observe different sensitivity of control and SLF2-deficient cell lines to TAK-243. We included the data in Appendix Figure S11C.

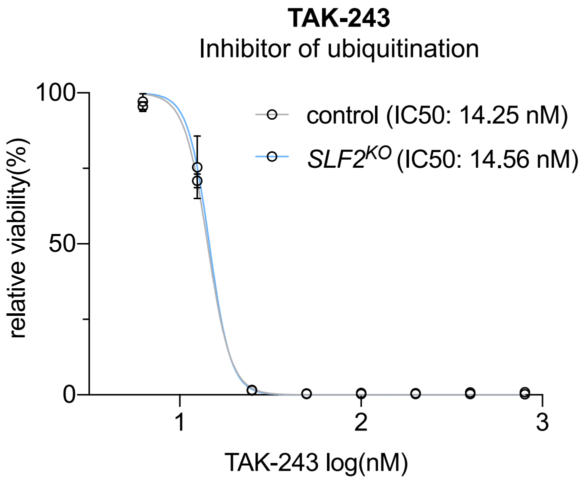

2. SLF1 and SLF2 are both prominent proteins in the cited paper by Räschle et al. 2015 Science. Here the authors only study SLF2 and ignore SLF1. The authors should compare SLF1 and SLF2 side by side and test whether they have overlapping or unique functions in the context of B-cell lymphomagenesis.

We thank Reviewer #3 for raising this question and would like to highlight that we specifically used the recently published *in vivo* transposon mutagenesis screening[10] to pre-filter for driver alterations with relevance during B-cell lymphomagenesis. Importantly, such screenings are providing a first layer of evidence for functional relevance in an in vivo setting. In this (functional) *in vivo* approach we specifically identified SLF2 but not SLF1 and therefore fully focused our study on SLF2. To further substantiate our approach, we have analyzed a large set of published transposon mutagenesis screenings based on the Sleeping Beauty Cancer Driver Data Base (https://sbcddb.moffitt.org). While *Slf2* seems frequently affected as a putative cancer driver (progression driver) in screens of various tumor indications, *Slf1* has been never found among progression drivers revealing that it is SLF2 that specifically plays a role in tumorigenesis.

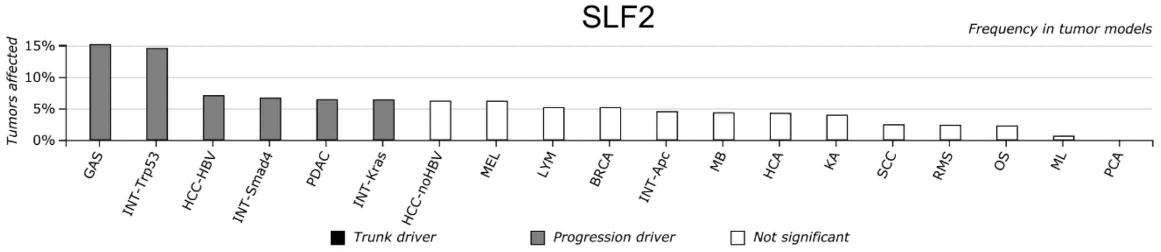

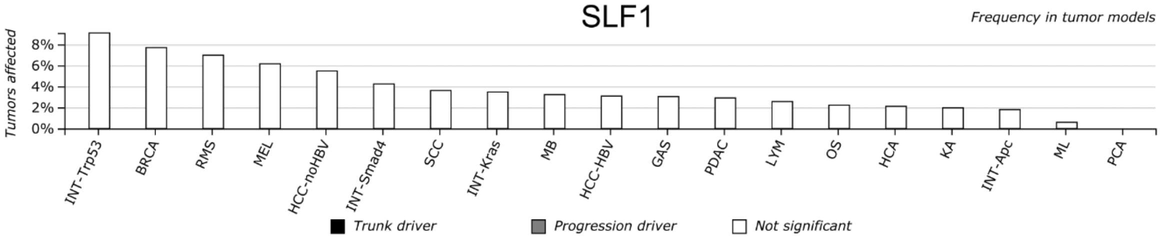

To further corroborate this, we now also analyzed the association of *SLF1* mRNA expression with outcome prognosis in DLBCL patients. While low SLF2 mRNA expression is associated with adverse prognosis in human DLBCL patients, SLF1 mRNA expression is not associated with prognosis in DLBCL patients. We now included these data in new Figure 3C.

Beyond this, while Räschle et al. described RAD18-SLF1-SLF2 as a complex recruiting the SMC5/6 complex to DNA lesions[10], we did not observe any alteration of the chromatin presence of SMC5 in SLF2-deficient B-cell lymphoma/cancer cells (new Figure 5F) and conclude that the mechanism we describe is independent of the one described in Räschle et al.

3. The authors claim activation of the post-translational modification SUMOylation by SLF2 deficiency. This claim is poorly substantiated. It would be important to strengthen this claim by demonstrating enhanced SUMOylation for a selected set of SUMO targets in the absence of SLF2.

We thank Reviewer #3 for raising this important question. Please see also our response to Reviewer #1:

Importantly, the SUMOylation pathway is regulated on various layers from SUMO conjugation controlled by E1 (SAE1/SAE2), E2 (UBE2I) and E3 ligases to de-SUMOylation, which is tightly controlled by SUMO isopeptidase. Therefore, high SUMO pathway activity does not automatically reflect in a high level of SUMOylated proteins as this pathway is heavily counteracted by SUMO isopeptidases. The SUMO pathway inhibitor TAK-981 directly targets the E1 complex. To directly link the activity of this part of the SUMO pathway with SLF2 loss, we investigated the expression levels of SAE1 and SAE2 in SLF2-deficient DLBCL cells. We found significantly higher SAE1 expression in SLF2 deficient DLBCL cells, thus directly linking SLF2 loss to SUMO conjugation pathway activation. Beyond that, we also observe significant activation of SAE1 expression in CLSPN-deficient DLBCL cell lines, again copying the effects of SLF2 loss. We included the data in Figure 6 and 7.

4. The synergy between SLF2 deficiency and inhibition of sumoylation in figure 5 is interesting, but potential synergy with inhibition of ubiquitination could be even more interesting as mentioned in point 1.

We thank Reviewer #3 for raising this important point, which we addressed experimentally. Please see the response to point 1 for a detailed discussion and data presentation.

5. The authors demonstrate synergy between SUMOi and either AZD6672 or rabusertib, which is poorly connected to SLF2 as the main topic of the study. Whereas the authors show increased polyploidy in cells that are deficient for SLF2 and are treated with SUMOi, the experiments with SUMOi and either AZD6672 or rabusertib only study apoptosis. The authors need to verify whether SUMOi and either AZD6672 or rabusertib also enhance polyploidy.

We thank Reviewer #3 for pointing us to this valuable experiment. With regard to this issue, we treated both SU-DHL-4 and OCI-Ly1 cell lines with SUMOi in combination with rabusertib and AZD7762 and performed PI cell cycle analysis to assess polyploidy. Importantly, both combinations enhanced the polyploidy of both SU-DHL4 and OCI-Ly1 cells with a similar effect as we observed in SUMOi treated SLF2-deficient cell lines. We included the data in Figure 8D and Appendix Figure S14.

6. Subasumstat is associated with activation of the immune system (reference 38), this could be mentioned in the paper. Since Subasumstat in vivo primarily acts via the immune system, it is doubtful whether the uncovered drug synergy can be confirmed in a syngeneic mouse B-cell lymphoma model. Convincing proof that the drug synergy is also efficient in a mouse B-cell lymphoma model would strengthen the manuscript considerably.

We thank Reviewer #3 for raising this important point. However, we would like to underscore that we can not agree with the notion that the SUMOylation inhibitor subasumstat acts in vivo primarily via the immune system. While subasumstat clearly affects the immunes system and alters immune responses this[11-14] (two of these publications are from our lab), we and others have published the tumor-intrinsic effects on cell death by SUMOi *in vitro* and in immune-compromised mouse models like in Biederstädt et al.[9] (ML-93 is a corresponding pre-clinical version of the molecule TAK-981 (subasumstat) or Heynen et al.[15]. We mention this now specifically in the discussion section of our manuscript.

In summary, the current state regarding the mode-of-action of subasumstat is characterized by various axis ultimately inducing cancer cell death: 1) tumor-intrinsic induction of cell death; 2) activation of the anti-tumor immune response by directly activating immune cells like T-cells and NK-cells and 3) activation of MHC-I based antigen-presentation on tumor cells and thus activating CD8-positive cytotoxic T-cells.

Therefore, we are convinced that *in vitro* systems are highly relevant tools which are essential to dissect the mode-of-action of subasumstat. Further, as use of complex *in vivo* systems will not allow deducting if the observed efficacy is due to e.g. activation of the immune system by both SUMOi and potentially also inhibitors of the DNA damage response or by tumor cell death induced by the synergism of the drug combination.

To address the Reviewer's question, we perfomed two sets of in vivo experiments.

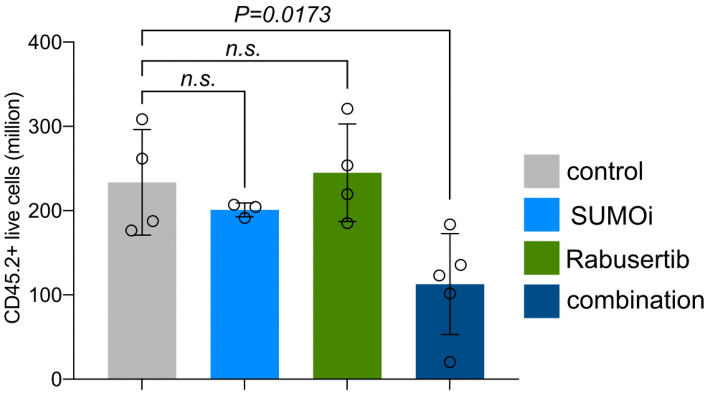

First, we transplanted primary *Eμ-Myc* lymphomas (CD45.2) into syngeneic CD45.1 recipient mice and treated the mice with either carrier control, SUMOi, rabusertib, or the combination treatment. Subsequent to the treatment (48 hours) we analyzed the absolute numbers of lymphoma cells from the spleens by FACS and indeed observed a significant reduction of lymphoma cell numbers only in mice treated with the drug combination, while not in the mice treated with either SUMOi or rabusertib alone. However, based on the concerns discussed and raised above, we did not include this experiment in the manuscript.

Secondly, and more important since not affecting the specific immune response, and to prove the synergism for tumor cell killing by a tumor-intrinsic mechanism *in vivo*, we generated xenografts of the OCI-Ly1 DLBCL cell line and treated the mice with carrier control, the SUMOi subasumstat, rabusertib (CHK1i), or the combination treatment and monitored tumor growth. Of note, the combination treatment led to significantly impaired tumor growth in mice treated with the combination treatment when compared to either SUMOi or rabusertib alone. These data are shown in the revised version of the manuscript as new Figure 8I and 8J.

7. The entire manuscript deals with B cell lymphoma. It would therefore be important to replace experiments that are carried out with non B cell lymphoma cells for experiments with B cell lymphoma cells.

We thank the Reviewer for pointing this out. We now carried out all crucial experiments based on B-cell lymphoma cells and revised the manuscript accordingly. We also suggest to keep the crucial non B-cell lymphoma cell-based experiments within the manuscript since in our opinion this data strengthen the relevance of the described mechanism across tumor indications, thus also broadening potential translation.

8. For many experiments, it is unclear how many times they have been performed. Indicating reproducibility of all experiments is therefore vital.

We have revised the manuscript accordingly and indicated the number of independent experiments for every experiment in the respective figure legend.

Minor comments:

9. The Western blots are very tightly cropped. Please crop less tightly and provide relevant size markers.

We revised the manuscript and cropping of the western blot accordingly. Moreover, we provide all fully uncropped scans as source data accompanying the revised manuscript.

10. Adding a mechanistic cartoon in the final figure would be helpful.

We thank the Reviewer for this valuable suggestion. We included a mechanistic cartoon as Appendix Figure S11D, which will also be used as graphical abstract of the manuscript.

1. Dupont, L., *et al.* The SMC5/6 complex compacts and silences unintegrated HIV-1 DNA and is antagonized by Vpr. *Cell Host Microbe* **29**, 792-805 e796 (2021).
2. Bergink, S. & Jentsch, S. Principles of ubiquitin and SUMO modifications in DNA repair. *Nature* **458**, 461-467 (2009).
3. Sogo, J.M., Lopes, M. & Foiani, M. Fork reversal and ssDNA accumulation at stalled replication forks owing to checkpoint defects. *Science* **297**, 599-602 (2002).
4. Liao, H., Ji, F., Helleday, T. & Ying, S. Mechanisms for stalled replication fork stabilization: new targets for synthetic lethality strategies in cancer treatments. *EMBO Rep* **19**(2018).
5. Zou, L. Single- and double-stranded DNA: building a trigger of ATR-mediated DNA damage response. *Genes Dev* **21**, 879-885 (2007).
6. Glover, T.W., Berger, C., Coyle, J. & Echo, B. DNA polymerase alpha inhibition by aphidicolin induces gaps and breaks at common fragile sites in human chromosomes. *Hum Genet* **67**, 136-142 (1984).
7. Somyajit, K., Saxena, S., Babu, S., Mishra, A. & Nagaraju, G. Mammalian RAD51 paralogs protect nascent DNA at stalled forks and mediate replication restart. *Nucleic Acids Res* **43**, 9835-9855 (2015).
8. Bric, A., *et al.* Functional identification of tumor-suppressor genes through an in vivo RNA interference screen in a mouse lymphoma model. *Cancer Cell* **16**, 324-335 (2009).
9. Liu, Q., *et al.* Chk1 is an essential kinase that is regulated by Atr and required for the G(2)/M DNA damage checkpoint. *Genes Dev* **14**, 1448-1459 (2000).
10. Schick, M., *et al.* Genetic alterations of the SUMO isopeptidase SENP6 drive lymphomagenesis and genetic instability in diffuse large B-cell lymphoma. *Nat Commun* **13**, 281 (2022).
11. Demel, U.M., *et al.* Activated SUMOylation restricts MHC class I antigen presentation to confer immune evasion in cancer. *J Clin Invest* **132**(2022).
12. Kumar, S., *et al.* Targeting pancreatic cancer by TAK-981: a SUMOylation inhibitor that activates the immune system and blocks cancer cell cycle progression in a preclinical model. *Gut* **71**, 2266-2283 (2022).
13. Lightcap, E.S., *et al.* A small-molecule SUMOylation inhibitor activates antitumor immune responses and potentiates immune therapies in preclinical models. *Sci Transl Med* **13**, eaba7791 (2021).
14. Demel, U.M., *et al.* Small-molecule SUMO inhibition for biomarker-informed B-cell lymphoma therapy. *Haematologica* **108**, 555-567 (2023).
15. Heynen, G., *et al.* SUMOylation inhibition overcomes proteasome inhibitor resistance in multiple myeloma. *Blood Adv* **7**, 469-481 (2023).

9th Jun 2023

Dear Dr. Schick,

Thank you for the submission of your revised manuscript to EMBO Molecular Medicine. We have now received the reports from the 3 referees. As you will see, they are supportive of publication pending minor revisions, and we will therefore be able to accept your manuscript once the following points will be addressed:

1/ Referees' comments: please address the remaining concerns from referees #1 and #3, if possible experimentally when needed. Alternatively, please address each point in writing.

2/ Main manuscript text:
- Please remove the red text, accept all changes, and only keep in track changes mode any new modification.
- Please remove "Summary" from the "Paper Explained" headline.
- Materials and methods:
o Animal experiments: please indicate the housing and husbandry conditions for the mice (night/light cycle, diet).
o Cell culture: please indicate the origin of the cells.
o Antibodies: please indicate the dilutions/concentrations used.
o Statistical analysis: please include a statement about randomization, blinding and exclusion/inclusion criteria, and adjust the checklist accordingly.
- Data Availability Section: Thank you for providing links with reviewer tokens. Please note that the data must be public before acceptance of the manuscript.

3/ Figures and Appendix:
- Appendix: Kindly note that you have the possibility to make some of the Appendix figures "Expanded View Figures" that are collapsible/expandable online. A maximum of 5 EV Figures can be typeset. EV Figures should be cited as 'Figure EV1, Figure EV2" etc... in the text and their respective legends should be included in the main text after the legends of regular figures.
- Figure legends should mention (parts of) figures that are used in different figures: Blots from Appendix fig S12i is reused from 5B, not 5D, please correct. Appendix Fig. S12E also reuses blot from Appendix Fig S12G, please mention it in the legend.

4/ Synopsis: I slightly edited your text to fit our style and format, please let me know if you agree with the following or amend as you see fit:

SLF2 was identified as a tumor suppressor of B-cell lymphomagenesis and a crucial regulator of CHK1, and its deficiency was associated with defective DNA damage response and synthetic lethality to SUMOylation inhibition.

- SLF2 is a functionally relevant tumor suppressor in murine and human B-cell lymphoma
- Loss of SLF2 results in CHK1 impairment, transcriptional repression of Claspin, and genomic instability
- SLF2 deficiency drives alteration of the SUMO pathway and confers synthetic lethality to pharmacological SUMOylation inhibition
- Impaired DNA damage responses, e.g. caused by SLF2 loss or pharmacological inhibition of CHK1, confer synthetic lethality to SUMO inhibition

Thank you for providing a nice synopsis picture. Please upload it as a PNG/JPEG/TIFF file 550 px wide x 300-600 px high.

5/ As part of the EMBO Publications transparent editorial process initiative (see our Editorial at http://embomolmed.embopress.org/content/2/9/329), EMBO Molecular Medicine will publish online a Review Process File (RPF) to accompany accepted manuscripts.
This file will be published in conjunction with your paper and will include the anonymous referee reports, your point-by-point response and all pertinent correspondence relating to the manuscript. Let us know whether you agree with the publication of the RPF and as here, if you want to remove or not any figures from it prior to publication.
Please note that the Authors checklist will be published at the end of the RPF.

I look forward to receiving your revised manuscript.

Yours sincerely,

Lise Roth

Lise Roth, PhD

Senior Editor
EMBO Molecular Medicine

The system will prompt you to fill in your funding and payment information. This will allow Wiley to send you a quote for the article processing charge (APC) in case of acceptance. This quote takes into account any reduction or fee waivers that you may be eligible for. Authors do not need to pay any fees before their manuscript is accepted and transferred to our publisher.

***** Reviewer's comments *****

Referee #1 (Remarks for Author):

Zhang and co-workers identified SLF2 as a tumor suppressor for MYC-driven B-cell lymphoma. The authors claimed that a loss of SLF2 associated with a high level of DNA damage could activate sumoylation pathway in part via SAE1 upregulation. They proposed co-targeting of the sumoylation and DDR pathways is a promising strategy for the treatment of aggressive lymphoma. In this revised manuscript, the authors provided evidence that SLF2 loss resulted in a reduced expression of CLSPN, Aurora-A and -B, PTTG-1 and FANCD2, etc. Then, the authors further pointed out a reduction of CLSPN mRNA in SLF2 knockout cells and claimed the regulation is transcriptional. The authors speculated a reduction of CLSPN is the main cause of SLF2 loss-elicited defective CHK1 phosphorylation. Several studies have demonstrated that Aurora-A and -B, ATR, CHK1 and SUMO machinery SAE1 and SAE2 are synthetic partners for Myc (a review EMBO Report 17: 1516-1531 (2016)). Thus, the novelty of these findings is as to how SLF2 interplays with these synthetic partners with a clear storyline. However, the current format is still quite fragmented. Several suggestions to improve the quality of this manuscript are as following:

1. The alteration of CLSPN mRNA levels by SLF2 could be transcriptional and post-transcriptional. Why did the authors claim the regulation is transcriptional?
2. If SLF2 loss caused a decrease of CHK1 phosphorylation is due to a reduction of CLSPN, Whether re-expression of CLSPN in SLF2 knockout cell can restore the CHK1 phosphorylation and also regain the cell phenotype.
3. Because SLF2 loss resulted in the alteration of several Myc synthetic partners including mitotic stress (such as SUMOylation), DNA damage, and replication stress (such as ATR and CHK1), it is not surprising that the usage of two inhibitors has synergistic effect in treating BCL.
4. Whether SAE1 downregulation could attenuate the effect caused by SLF2 loss. How does SLF2 affect SAE1 expression? Please show SLF2 loss in altering endogenous sumoylation levels by Western analysis.
5. Please delete NSMCE2 data since the authors claimed that SLF2 did not directly impact on NSMCE2, which makes the storyline more clean.

Minor
1. Since SLF2 is not in the list of Fig. S1 and S2. I do not see the point to include these two figures in the appendix
2. In Fig. 5 C and D, please show the entire Y axis from zero.
3. No loading control in Fig. S9

Referee #2 (Remarks for Author):

The Authors have addressed all my concerns with extensive experimental work. I send them my compliment for the impressive effort which led to a considerable improvement of the manuscript.

Referee #3 (Remarks for Author):

The authors have done an excellent job revising their manuscript. Almost all concerns are properly addressed. Two points require attention:

Firstly, the SUMO pathway connection in the project is still suboptimal. The authors identify a modest but significant increase in SAE1, but not SAE2 upon SLF2 ko and CLSPN ko (Figures 6C, 6D, 7D and 7E). This is interesting and helpful. However, since the SUMO E1 is dimeric, consisting of SAE1 and SAE2, an increase in one of these components, but not in the other component would not necessarily represent activation of the pathway. To address whether overall SUMO conjugation levels are increased upon SLF2 ko and CLSPN ko, the authors need to verify in total lysates by immunoblotting whether SUMO1 and/or SUMO2/3 conjugates are increased modestly but significantly or not at all. If there is no increase in overall sumoylation, then it would be important to check protein levels and activity of the SUMO proteases as well to properly investigate the enzymatic

components of the SUMO pathway in these cells.

Secondly, the authors have performed an interesting experiment in a syngeneic mouse model, showing synergy between Rabusertib and subasumstat. This is a relevant experiment that needs to be included in the manuscript, not only in the rebuttal.

Point-by-point response to the Reviewer´s comments

Referee #1 (Remarks for Author):

Zhang and co-workers identified SLF2 as a tumor suppressor for MYC-driven B-cell lymphoma. The authors claimed that a loss of SLF2 associated with a high level of DNA damage could activate sumoylation pathway in part via SAE1 upregulation. They proposed co-targeting of the sumoylation and DDR pathways is a promising strategy for the treatment of aggressive lymphoma. In this revised manuscript, the authors provided evidence that SLF2 loss resulted in a reduced expression of CLSPN, Aurora-A and -B, PTTG-1 and FANCD2, etc. Then, the authors further pointed out a reduction of CLSPN mRNA in SLF2 knockout cells and claimed the regulation is transcriptional. The authors speculated a reduction of CLSPN is the main cause of SLF2 loss-elicited defective CHK1 phosphorylation. Several studies have demonstrated that Aurora-A and -B, ATR, CHK1 and SUMO machinery SAE1 and SAE2 are synthetic partners for Myc (a review EMBO Report 17: 1516-1531 (2016)). Thus, the novelty of these findings is as to how SLF2 interplays with these synthetic partners with a clear storyline. However, the current format is still quite fragmented. Several suggestions to improve the quality of this manuscript are as following:

We thank Reviewer #1 for the positive reception of our manuscript and for appreciating the clear storyline.

1. The alteration of CLSPN mRNA levels by SLF2 could be transcriptional and post-transcriptional. Why did the authors claim the regulation is transcriptional?

We thank the reviewer for his/her comment. Accordingly, we rephrased the original sentence and included “transcriptional or post-transcriptional” in the revised manuscript.

2. If SLF2 loss caused a decrease of CHK1 phosphorylation is due to a reduction of CLSPN. Whether re-expression of CLSPN in SLF2 knockout cell can restore the CHK1 phosphorylation and also regain the cell phenotype.

The role of CLSPN in promoting CHK1 phosphorylation is fully established and extensively described in the literature (Reviewed for example here: (Smits *et al*, 2019)). Despite of that, we generated genetic knockouts of *CLSPN* to further corroborate the role of CLSPN for CHK1 phosphorylation in B-cell lymphoma. Moreover, we show that *CLSPN* deficiency confers sensitivity to SUMO inhibition loss and alterations of the SUMO pathway like *SLF2* loss. We conclude that the depletion of *CLSPN* is sufficient to lead to similar biology as caused by *SLF2* loss (Data shown in Figure 7).

Importantly, we still cannot exclude that also other proteins dysregulated following *SLF2* loss may have an impact on CHK1 phosphorylation and included this sentence to the discussion: “We identified a critical role for CLSPN in the phosphorylation of CHK1 in the SLF2 context. At this point, we cannot exclude the possibility that other factors may also contribute to CHK1 phosphorylation.”

3. Because SLF2 loss resulted in the alteration of several Myc synthetic partners including mitotic stress (such as SUMOylation), DNA damage, and replication stress (such as ATR and CHK1), it is not surprising that the usage of two inhibitors has synergistic effect in treating BCL.

Revealing synergy between SUMOi and CHK1i is a novel finding with potential clinical implications. We agree with the Reviewer that this might have been conceivable based on our findings described throughout the manuscript and provide in vitro/in vivo experimental proof based on our findings.

4. Whether SAE1 downregulation could attenuate the effect caused by SLF2 loss. How does SLF2 affect SAE1 expression? Please show SLF2 loss in altering endogenous sumoylation levels by Western analysis.

Although we consider this as a potentially interesting experiment, the technical feasibility is limited as SAE1 is a common essential gene with 1084 out of 1085 profiled cancer cell lines showing dependency (https://depmap.org/portal/gene/SAE1?tab=overview). Importantly, our data show that

SLF2-deficient cell lines even show a higher dependency of the SUMOylation pathway (SAE1 is among the rate-limiting and critical enzymes of the SUMOylation pathway and the SUMOylation inhibitor we use directly affects the SAE1/SAE2 complex), which makes this experiment in our opinion not feasible.

A systematic elucidation of the SUMO core signaling pathway in the context of SLF2 would need to be worked on using different inducible genetic and proteolytic methodologies, as loss of individual SUMO core components leads to complete loss of viability. We believe that such an approach needs to be tested in different cell lines, tumor entities and possibly in cell-free systems. In order to answer this question satisfactorily, we believe that very extensive experiments would have to be performed, which are beyond the scope of the present manuscript.

We however provide evidence that SLF2 loss leads to an actionable molecular vulnerability conferring sensitivity to inhibition of SUMOylation in two independent cell lines (both with genetically defined SLF2 status by CRISPR/Cas9 gene editing). Considering that hundreds of proteins can be SUMOylated and that typically only a small proportion of the protein pool of a given protein is SUMOylated at a given time, we do not consider western blot analysis a conclusive approach to determine the activity of the SUMO pathway. Moreover, the SUMO pathway is heavily counter-regulated by various SUMO iso-peptidases (Nayak & Muller, 2014; Seeler & Dejean, 2017).

We would also like to note that we included this paragraph in the discussion of our manuscript: “Importantly, the SUMOylation pathway is regulated on various layers from SUMO conjugation controlled by E1 (SAE1/SAE2), E2 (UBE2I) and E3 ligases to de-SUMOylation, which is tightly controlled by SUMO isopeptidases (Kunz et al, 2018; Seeler & Dejean, 2017). The increase in SAE1 expression and potentially SUMO pathway activity may not automatically result in a high level of SUMOylated target proteins as this pathway is heavily counteracted by SUMO isopeptidases (Kunz et al., 2018; Schick et al., 2022).”

5. Please delete NSMCE2 data since the authors claimed that SLF2 did not directly impact on NSMCE2, which makes the storyline more clean.

We revised the manuscript according to the Reviewer´s comment and removed the NSCMCE2 data (Appendix Figure S12) and corresponding text from the manuscript.

Minor
1. Since SLF2 is not in the list of Fig. S1 and S2. I do not see the point to include these two figures in the appendix

Following the previous Reviewer suggestions, we decided to keep Appendix Figure S1 and Appendix Figure S2 in the manuscript appendix.

2. In Fig. 5 C and D, please show the entire Y axis from zero.

We revised Figures 5C and 5D accordingly and show the entire Y axis from zero.

3. No loading control in Fig. S9

We show Ponceau S staining of the whole membrane as a loading control.

Referee #2 (Remarks for Author):

The Authors have addressed all my concerns with extensive experimental work. I send them my compliment for the impressive effort which led to a considerable improvement of the manuscript.

We thank Reviewer #2 for the very positive reception of our work.

Referee #3 (Remarks for Author):

The authors have done an excellent job revising their manuscript. Almost all concerns are properly addressed. Two points require attention:

We thank Reviewer #3 and are pleased by the positive feedback for our work.

Firstly, the SUMO pathway connection in the project is still suboptimal. The authors identify a modest but significant increase in SAE1, but not SAE2 upon SLF2 ko and CLSPN ko (Figures 6C, 6D, 7D and 7E). This is interesting and helpful. However, since the SUMO E1 is dimeric, consisting of SAE1 and SAE2, an increase in one of these components, but not in the other component would not necessarily represent activation of the pathway. To address whether overall SUMO conjugation levels are increased upon SLF2 ko and CLSPN ko, the authors need to verify in total lysates by immunoblotting whether SUMO1 and/or SUMO2/3 conjugates are increased modestly but significantly or not at all. If there is no increase in overall sumoylation, then it would be important to check protein levels and activity of the SUMO proteases as well to properly investigate the enzymatic components of the SUMO pathway in these cells.

We thank the reviewer for this comment. We would like to point out that we have already included substantial global proteome and transcriptome analyses in the manuscript to specifically investigate the biology mediating the effects of SLF2 loss. By this approach, we have identified CLSPN and validated that loss of CLSPN leads to similar effects like SLF2 loss. In combination with the pathway analysis performed based on our global analysis and the literature, we agree with Reviewer 1 that our findings clearly suggested this co-dependency.

As discussed in our response to Reviewer 1 (point 4), a systematic elucidation of the SUMO core signaling pathway in the context of SLF2 would require very extensive experiments, which we believe are beyond the scope of the present manuscript.

As noted in our response to Reviewer 1, we included this paragraph to the discussion of our manuscript: “Importantly, the SUMOylation pathway is regulated on various layers from SUMO conjugation controlled by E1 (SAE1/SAE2), E2 (UBE2I) and E3 ligases to de-SUMOylation, which is tightly controlled by SUMO isopeptidases (Kunz et al, 2018; Seeler & Dejean, 2017). The increase in SAE1 expression and potentially SUMO pathway activity may not automatically result in a high level of SUMOylated target proteins as this pathway is heavily counteracted by SUMO isopeptidases (Kunz et al., 2018; Schick et al., 2022).”

Secondly, the authors have performed an interesting experiment in a syngeneic mouse model, showing synergy between Rabusertib and subasumstat. This is a relevant experiment that needs to be included in the manuscript, not only in the rebuttal.

We included the data in the manuscript according to the Reviewers´ suggestions.

REFERENCES

Nayak A, Muller S (2014) SUMO-specific proteases/isopeptidases: SENPs and beyond. *Genome Biol* 15: 422

Seeler JS, Dejean A (2017) SUMO and the robustness of cancer. *Nat Rev Cancer* 17: 184-197

Smits VAJ, Cabrera E, Freire R, Gillespie DA (2019) Claspin - checkpoint adaptor and DNA replication factor. *FEBS J* 286: 441-455

6th Jul 2023

Dear Dr. Schick,

Thank you for submitting your revised files. I am pleased to inform you that your manuscript is accepted for publication and is now being sent to our publisher to be included in the next available issue of EMBO Molecular Medicine!

Please read below for additional IMPORTANT information regarding your article, its publication and the production process.

Congratulations on your interesting work!

With kind regards,

Lise Roth

Lise Roth, Ph.D
Senior Editor
EMBO Molecular Medicine

Follow us on Twitter @EmboMolMed
Sign up for eTOCs at embopress.org/alertsfeeds

*** *** *** IMPORTANT INFORMATION *** *** ***

SPEED OF PUBLICATION
The journal aims for rapid publication of papers, using using the advance online publication "Early View" to expedite the process: A properly copy-edited and formatted version will be published as "Early View" after the proofs have been corrected. Please help the Editors and publisher avoid delays by providing e-mail address(es), telephone and fax numbers at which author(s) can be contacted.

Should you be planning a Press Release on your article, please get in contact with embomolmed@wiley.com as early as possible, in order to coordinate publication and release dates.

LICENSE AND PAYMENT:

All articles published in EMBO Molecular Medicine are fully open access: immediately and freely available to read, download and share.

EMBO Molecular Medicine charges an article processing charge (APC) to cover the publication costs. You, as the corresponding author for this manuscript, should have already received a quote with the article processing fee separately. Please let us know in case this quote has not been received.

Once your article is at Wiley for editorial production you will receive an email from Wiley's Author Services system, which will ask you to log in and will present you with the publication license form for completion. Within the same system the publication fee can be paid by credit card, an invoice, pro forma invoice or purchase order can be requested.

Payment of the publication charge and the signed Open Access Agreement form must be received before the article can be published online.

PROOFS

You will receive the proofs by e-mail approximately 2 weeks after all relevant files have been sent o our Production Office. Please return them within 48 hours and if there should be any problems, please contact the production office at embopressproduction@wiley.com.

Please inform us if there is likely to be any difficulty in reaching you at the above address at that time. Failure to meet our deadlines may result in a delay of publication.

All further communications concerning your paper proofs should quote reference number EMM-2022-16431-V3 and be directed to the production office at embopressproduction@wiley.com.

Corresponding Author Name: Markus Schick, Ulrich Keller	
Journal Submitted to: EMBO Molecular Medicine	
Manuscript Number: #EMM-2022-16431-V2	

USEFUL LINKS FOR COMPLETING THIS FORM
The EMBO Journal - Author Guidelines
EMBO Reports - Author Guidelines
Molecular Systems Biology - Author Guidelines
EMBO Molecular Medicine - Author Guidelines

Reporting Checklist for Life Science Articles (updated January
This checklist is adapted from Materials Design Analysis Reporting (MDAR) Checklist for Authors. MDAR establishes a minimum set of requirements in transparent reporting in the life sciences (see Statement of Task: 10.31222/osf.io/9sm4x). Please follow the journal's guidelines in preparing your manuscript.
Please note that a copy of this checklist will be published alongside your article.

Abridged guidelines for figures
1. Data
The data shown in figures should satisfy the following conditions:
- the data were obtained and processed according to the field's best practice and are presented to reflect the results of the experiments in an accurate and unbiased manner.
- ideally, figure panels should include only measurements that are directly comparable to each other and obtained with the same assay.
- plots include clearly labeled error bars for independent experiments and sample sizes. Unless justified, error bars should not be shown for technical
- if n<5, the individual data points from each experiment should be plotted. Any statistical test employed should be justified.
- Source Data should be included to report the data underlying figures according to the guidelines set out in the authorship guidelines on Data

2. Captions
Each figure caption should contain the following information, for each panel where they are relevant:
- a specification of the experimental system investigated (eg cell line, species name).
- the assay(s) and method(s) used to carry out the reported observations and measurements.
- an explicit mention of the biological and chemical entity(ies) that are being measured.
- an explicit mention of the biological and chemical entity(ies) that are altered/varied/perturbed in a controlled manner.
- the exact sample size (n) for each experimental group/condition, given as a number, not a range;
- a description of the sample collection allowing the reader to understand whether the samples represent technical or biological replicates (including how many animals, litters, cultures, etc.).
- a statement of how many times the experiment shown was independently replicated in the laboratory.
- definitions of statistical methods and measures:
  - common tests, such as t-test (please specify whether paired vs. unpaired), simple χ2 tests, Wilcoxon and Mann-Whitney tests, can be unambiguously identified by name only, but more complex techniques should be described in the methods section;
  - are tests one-sided or two-sided?
  - are there adjustments for multiple comparisons?
  - exact statistical test results, e.g., P values = x but not P values < x;
  - definition of 'center values' as median or average;
  - definition of error bars as s.d. or s.e.m.

Please complete ALL of the questions below.
Select "Not Applicable" only when the requested information is not relevant for your study.

Materials

Newly Created Materials	Information included in the manuscript?	In which section is the information available? (Reagents and Tools Table, Materials and Methods, Figures, Data Availability Section)
New materials and reagents need to be available; do any restrictions apply?	Not Applicable	

Antibodies	Information included in the manuscript?	In which section is the information available? (Reagents and Tools Table, Materials and Methods, Figures, Data Availability Section)
For **antibodies** provide the following information:<br>- Commercial antibodies: RRID (if possible) or supplier name, catalogue number and or/clone number<br>- Non-commercial: RRID or citation	Yes	Material and Methods

DNA and RNA sequences	Information included in the manuscript?	In which section is the information available? (Reagents and Tools Table, Materials and Methods, Figures, Data Availability Section)
**Short novel DNA or RNA including primers, probes**: provide the sequences.	Yes	Material and Methods

Cell materials	Information included in the manuscript?	In which section is the information available? (Reagents and Tools Table, Materials and Methods, Figures, Data Availability Section)
**Cell lines:** Provide species information, strain. Provide accession number in repository **OR** supplier name, catalog number, clone number, and/**OR** RRID.	Yes	Material and Methods
**Primary cultures:** Provide species, strain, sex of origin, genetic modification status.	Yes	Materials and Methods
Report if the cell lines were recently **authenticated** (e.g., by STR profiling) and tested for mycoplasma contamination.	Yes	Materials and Methods

Experimental animals	Information included in the manuscript?	In which section is the information available? (Reagents and Tools Table, Materials and Methods, Figures, Data Availability Section)
**Laboratory animals or Model organisms:** Provide species, strain, sex, age, genetic modification status. Provide accession number in repository **OR** supplier name, catalog number, clone number, **OR** RRID.	Yes	Material and Methods
**Animal observed in or captured from the field:** Provide species, sex, and age where possible.	Yes	Material and Methods
Please detail **housing and husbandry conditions**.	Yes	Material and Methods

Plants and microbes	Information included in the manuscript?	In which section is the information available? (Reagents and Tools Table, Materials and Methods, Figures, Data Availability Section)
**Plants:** provide species and strain, ecotype and cultivar where relevant, unique accession number if available, and source (including location for collected wild specimens).	Not Applicable	
**Microbes:** provide species and strain, unique accession number if available, and source.	Not Applicable	

Human research participants	Information included in the manuscript?	In which section is the information available? (Reagents and Tools Table, Materials and Methods, Figures, Data Availability Section)
If collected and within the bounds of privacy constraints report on age, sex and gender or ethnicity for all study participants.	Not Applicable	

Core facilities	Information included in the manuscript?	In which section is the information available? (Reagents and Tools Table, Materials and Methods, Figures, Data Availability Section)
If your work benefited from core facilities, was their service mentioned in the acknowledgments section?	Not Applicable	Novogene?

Design

Study protocol	Information included in the manuscript?	In which section is the information available? (Reagents and Tools Table, Materials and Methods, Figures, Data Availability Section)

If study protocol has been **pre-registered, provide DOI in the manuscript**. For clinical trials, provide the trial registration number **OR** cite DOI.	Not Applicable	
Report the **clinical trial registration number** (at ClinicalTrials.gov or equivalent), where applicable.	Not Applicable	

Laboratory protocol	Information included in the manuscript?	In which section is the information available? (Reagents and Tools Table, Materials and Methods, Figures, Data Availability Section)
Provide DOI OR other citation details if **external detailed step-by-step protocols** are available.	Not Applicable	

Experimental study design and statistics	Information included in the manuscript?	In which section is the information available? (Reagents and Tools Table, Materials and Methods, Figures, Data Availability Section)
Include a statement about **sample size** estimate even if no statistical methods were used.	Not Applicable	
Were any steps taken to minimize the effects of subjective bias when allocating animals/samples to treatment (e.g. **randomization procedure**)? If yes, have they been described?	Yes	Material and Methods
Include a statement about **blinding** even if no blinding was done.	Yes	Material and Methods
Describe **inclusion/exclusion criteria** if samples or animals were excluded from the analysis. Were the criteria pre-established?<br><br>If sample or data points were omitted from analysis, report if this was due to attrition or intentional exclusion and provide justification.	Yes	Material and Methods
For every figure, are **statistical tests** justified as appropriate? Do the data meet the assumptions of the tests (e.g., normal distribution)? Describe any methods used to assess it. Is there an estimate of variation within each group of data? Is the variance similar between the groups that are being statistically compared?	Yes	Figure legends

Sample definition and in-laboratory replication	Information included in the manuscript?	In which section is the information available? (Reagents and Tools Table, Materials and Methods, Figures, Data Availability Section)
In the figure legends: state number of times the experiment was **replicated** in laboratory.	Yes	Figure legends
In the figure legends: define whether data describe **technical or biological replicates**.	Yes	Figure legends

Ethics

Ethics	Information included in the manuscript?	In which section is the information available? (Reagents and Tools Table, Materials and Methods, Figures, Data Availability Section)
Studies involving **human participants**: State details of **authority granting ethics approval** (IRB or equivalent committee(s), provide reference number for approval.	Not Applicable	
Studies involving **human participants**: Include a statement confirming that **informed consent** was obtained from all subjects and that the experiments conformed to the principles set out in the WMA Declaration of Helsinki and the Department of Health and Human Services Belmont Report.	Not Applicable	
Studies involving **human participants:** For publication of **patient photos**, include a statement confirming that consent to publish was obtained.	Not Applicable	
Studies involving experimental **animals**: State details of **authority granting ethics approval** (IRB or equivalent committee(s), provide reference number for approval. Include a statement of compliance with ethical regulations.	Yes	Material and Methods
Studies involving **specimen and field samples:** State if relevant **permits** obtained, provide details of authority approving study; if none were required, explain why.	Not Applicable	

Dual Use Research of Concern (DURC)	Information included in the manuscript?	In which section is the information available? (Reagents and Tools Table, Materials and Methods, Figures, Data Availability Section)
Could your study fall under dual use research restrictions? Please check biosecurity documents and list of **select agents and toxins** (CDC): https://www.selectagents.gov/sat/list.htm	Not Applicable	
If you used a select agent, is the security level of the lab appropriate and reported in the manuscript?	Not Applicable	
If a study is subject to dual use research of concern regulations, is the name of the **authority granting approval and reference number** for the regulatory approval provided in the manuscript?	Not Applicable	

Reporting

The MDAR framework recommends adoption of discipline-specific guidelines, established and endorsed through community initiatives. Journals have their own policy about requiring specific guidelines and recommendations to complement MDAR.

Adherence to community standards	Information included in the manuscript?	In which section is the information available? (Reagents and Tools Table, Materials and Methods, Figures, Data Availability Section)
State if relevant guidelines or checklists (e.g., **ICMJE, MIBBI, ARRIVE, PRISMA**) have been followed or provided.	Not Applicable	
For **tumor marker prognostic studies,** we recommend that you follow the **REMARK** reporting guidelines (see link list at top right). See author guidelines, under 'Reporting Guidelines'. Please confirm you have followed these guidelines.	Not Applicable	
For **phase II and III randomized controlled trials**, please refer to the **CONSORT** flow diagram (see link list at top right) and submit the CONSORT checklist (see link list at top right) with your submission. See author guidelines, under 'Reporting Guidelines'. Please confirm you have submitted this list.	Not Applicable	

Data Availability

Data availability	Information included in the manuscript?	In which section is the information available? (Reagents and Tools Table, Materials and Methods, Figures, Data Availability Section)
Have **primary datasets** been deposited according to the journal's guidelines (see 'Data Deposition' section) and the respective accession numbers provided in the Data Availability Section?	Yes	Data Availability Section
Were **human clinical and genomic datasets** deposited in a public access-controlled repository in accordance to ethical obligations to the patients and to the applicable consent agreement?	Not Applicable	
Are **computational models** that are central and integral to a study available without restrictions in a machine-readable form? Were the relevant accession numbers or links provided?	Not Applicable	
If publicly available data were reused, provide the respective **data citations in the reference list**.	Yes	Material and Methods, Acknowledgements and Reference List
